# Cytoplasmic TP53INP2 acts as an apoptosis partner in TRAIL treatment: the synergistic effect of TRAIL with venetoclax in TP53INP2-positive acute myeloid leukemia

**DOI:** 10.1186/s13046-024-03100-0

**Published:** 2024-06-22

**Authors:** Jun Ren, Junpeng Huang, Zailin Yang, Minghui Sun, Jing Yang, Can Lin, Fangfang Jin, Yongcan Liu, Lisha Tang, Jiayuan Hu, Xingyu Wei, Xinyi Chen, Zihao Yuan, Zesong Yang, Yanmeng Chen, Ling Zhang

**Affiliations:** 1 Key Laboratory of Laboratory Medical Diagnostics, Ministry of Education, College of Laboratory Medicine, Chongqing Medical University, Chongqing, 400016 China; 2 Department of Hematology-Oncology, Chongqing Key Laboratory of Translational Research for Cancer Metastasis and Individualized Treatment, Chongqing University Cancer Hospital, Chongqing, 400030 China; 3 Department of Hematology, The First Affiliated Hospital of Chongqing Medical University, Chongqing, 400016 China

**Keywords:** Acute myeloid leukemia, TRAIL, TP53INP2, Venetoclax, Nucleophosmin 1

## Abstract

**Background:**

Acute myeloid leukemia (AML) is a hematopoietic malignancy with poor outcomes, especially in older AML patients. Tumor necrosis factor-related apoptosis-inducing ligand (TRAIL) is considered a promising anticancer drug because it selectively induces the extrinsic apoptosis of tumor cells without affecting normal cells. However, clinical trials have shown that the responses of patients to TRAIL are significantly heterogeneous. It is necessary to explore predictable biomarkers for the preselection of AML patients with better responsiveness to TRAIL. Here, we investigated the critical role of tumor protein p53 inducible nuclear protein 2 (TP53INP2) in the AML cell response to TRAIL treatment.

**Methods:**

First, the relationship between TP53INP2 and the sensitivity of AML cells to TRAIL was determined by bioinformatics analysis of Cancer Cell Line Encyclopedia datasets, Cell Counting Kit-8 assays, flow cytometry (FCM) and cell line-derived xenograft (CDX) mouse models. Second, the mechanisms by which TP53INP2 participates in the response to TRAIL were analyzed by Western blot, ubiquitination, coimmunoprecipitation and immunofluorescence assays. Finally, the effect of TRAIL alone or in combination with the BCL-2 inhibitor venetoclax (VEN) on cell survival was explored using colony formation and FCM assays, and the effect on leukemogenesis was further investigated in a patient-derived xenograft (PDX) mouse model.

**Results:**

AML cells with high TP53INP2 expression were more sensitive to TRAIL in vitro and in vivo. Gain- and loss-of-function studies demonstrated that TP53INP2 significantly enhanced TRAIL-induced apoptosis, especially in AML cells with nucleophosmin 1 (NPM1) mutations. Mechanistically, cytoplasmic TP53INP2 maintained by mutant NPM1 functions as a scaffold bridging the ubiquitin ligase TRAF6 to caspase-8 (CASP 8), thereby promoting the ubiquitination and activation of the CASP 8 pathway. More importantly, simultaneously stimulating extrinsic and intrinsic apoptosis signaling pathways with TRAIL and VEN showed strong synergistic antileukemic activity in AML cells with high levels of TP53INP2.

**Conclusion:**

Our findings revealed that TP53INP2 is a predictor of responsiveness to TRAIL treatment and supported a potentially individualized therapeutic strategy for TP53INP2-positive AML patients.

**Supplementary Information:**

The online version contains supplementary material available at 10.1186/s13046-024-03100-0.

## Background

Acute myeloid leukemia (AML) is a heterogeneous disease characterized by genetic abnormalities and epigenetic changes [1]. The identification of recurrent genetic alterations, such as FLT3-ITD, NPM1 and CEBPA, has helped refine individual prognosis and guide management [2–4]. Despite advances in supportive care, the primary treatment for AML remains a combination of cytarabine and anthracycline followed by allogeneic stem cell transplantation [5]. Notably, most elderly patients are often unable to tolerate such strategies, and their prognoses are particularly poor [6, 7]. Thus, it is important to optimize treatment regimens to improve the outcomes for this population.

Tumor necrosis factor-related apoptosis-inducing ligand (TRAIL) is a member of the tumor necrosis factor (TNF) family of death ligands, and it binds to death receptor 4/5 (DR4/5) to selectively initiate caspase-8 (CASP 8)-dependent extrinsic apoptosis in tumor cells without affecting normal cells [8, 9]. Currently, TRAIL is being tested in clinical trials as a novel drug for cancer treatment [10]. However, the outcomes of using TRAIL therapy have been disappointing due to the development of resistance to TRAIL in certain AML cells [11–13]. To address the clinical dilemma of TRAIL treatment, many sensitization strategies have been developed, including combination with traditional chemotherapy drugs, microRNA regulation or targeted small molecules such as the BCL-2 inhibitor venetoclax (VEN) [14–16]. Notably, the sensitivity of cancer cells to TRAIL-induced apoptosis may vary among different types of cells. AML patients exhibit high biological and clinical heterogeneity [17]. Therefore, identifying suitable biomarkers for the preselection of patients responsive to TRAIL appears to be an attractive option for the precise treatment of AML.

A rapidly increasing number of studies have focused on the mechanisms involved in the regulation of TRAIL-mediated cell death pathways. Roberts et al. [18] reported that the SCF^Skp2^ ubiquitin ligase complex modulates the response to TRAIL by promoting the ubiquitination and degradation of FLICE-like inhibitory protein in colon cancer cells. In another study, the downregulation of choline kinase alpha (CHKA) was shown to overcome TRAIL resistance in ovarian cancer cells by specifically increasing the expression of DR5 [19]. Recently, tumor protein p53-inducible nuclear protein 2 (TP53INP2), an autophagic factor, was reported to be correlated with TRAIL-induced apoptosis in breast cancer [20]. Of note, our previous findings revealed that TP53INP2 plays a pivotal oncogenic role in AML [21]. Thus, whether TP53INP2 affects TRAIL-induced cell apoptosis in AML remains to be defined.

In this study, we identified TP53INP2 as a crucial regulator of the response of AML cells to TRAIL treatment. Mechanistically, cytoplasmic TP53INP2 modulated by mutant NPM1 functions as a cooperating partner for TRAIL-mediated apoptosis by potentiating the activation of CASP 8 ubiquitination in a tumor necrosis factor (TNF) receptor-associated factor 6 (TRAF6)-dependent manner. In addition, TRAIL showed synergistic antileukemic effects with VEN in AML cells with high levels of TP53INP2 in vitro, a result that was further confirmed using an AML patient-derived xenograft (PDX) model. Our findings reveal that TP53INP2 may serve as a potential biomarker for the selection of AML patients who respond better to TRAIL alone or in combination with VEN therapy, which provides a potentially individualized therapeutic strategy for TP53INP2-positive AML patients.

## Methods

### Cell culture

The human myeloid leukemia cell lines OCI-AML2, OCI-AML3 and OCI-AML5 were obtained from Deutsche Sammlung von Mikroorganismen und Zellkulturen GmbH (Braunschweig, NI, Germany). The human myeloid leukemia cell lines KG-1, NB4, THP-1, HL-60, and U937 and the human embryonic kidney cell line HEK293T were obtained from the American Type Culture Collection (Manassas, VA, USA). The AML cell lines used in this study were authenticated using short tandem repeat profiling. These myeloid leukemia cells were cultured in RPMI-1640 medium (Gibco, USA) supplemented with 10% fetal bovine serum (Gibco, USA), 100 μg/mL streptomycin and 100 U/mL penicillin (Beyotime, China). HEK293T cells were maintained in Dulbecco’s modified Eagle’s medium (Gibco, USA) supplemented with 10% fetal bovine serum, 100 μg/mL streptomycin and 100 U/mL penicillin. All the cells were incubated at 37 °C in a humidified incubator supplemented with 5% CO_2_.

The cells were cultured in medium supplemented with recombinant human TRAIL (Peprotech, NJ, USA), the proteasome inhibitor MG-132 (Topscience, Shanghai, China), or the BCL-2 inhibitor venetoclax (MCE, Monmouth Junction, NJ, USA) and then collected for analysis.

### siRNA, plasmids, and cell transfection

The sequences of the lentivirus-based short hairpin RNAs (shRNAs) used were as follows: shTP53INP2#1, 5’-CCGGTCCAAGAACCAGAGCAG-3’; shTP53INP2#2, 5’-CGCCTTCGTGTCGGAGGAGGA-3’; shNPM1#1, 5’-GCCGACAAAGATTATCACTTT-3’; shNPM1#2: 5’-AGCAAGGTTCCACAGAAAA-3’; and shTRAF6: 5’-GCGCTGTGCAAACTATATATC-3’. Scramble shRNA was used as negative control (shNC), shRNAs were synthesized by Genechem Biotech Company (Shanghai, China). Flag-NPM1-wt and Flag-NPM1-mA plasmids were kindly provided by Dr. C.J. Sherr (Genetics and Tumor Cell Biology, St., Jude Children’s Research Hospital, Memphis, TN, USA). Plasmids encoding His-Ub-K63/48 and Flag-TRAF6 were obtained from Dr. H.K. Lin (Department of Molecular and Cellular Oncology, The University of Texas MD Anderson Cancer Center, Houston, TX, USA). pcDNA3.1-TP53INP2-HA, pcDNA3.1-CASP 8-Flag, and the empty vector pcDNA3.1 were constructed by Genecreate Biotech Company (Wuhan, China). For transfection, cells were seeded in a 6-well plate and transfected with the corresponding plasmids using Lipofectamine™ 2000 (Invitrogen, Carlsbad, CA, USA) according to the manufacturer’s instructions.

### Clinical samples

Bone marrow samples were collected from 20 AML patients at the First Affiliated Hospital of Chongqing Medical University and the Chongqing University Cancer Hospital. The clinical characteristics of the AML patients are shown in Additional file 1: Table S1. Approval was obtained from the ethics committee of Chongqing Medical University. This study was performed in compliance with the Declaration of Helsinki. Mononuclear cells were extracted by Ficoll Lymphocyte Separation Solution (TBD Science, Tianjin, China) according to the manufacturer’s instructions. Briefly, the samples were diluted with equal volumes of phosphate-buffered saline (PBS). The blood suspensions were mixed with Ficoll Lymphocyte Separation Solution and centrifuged to form a discrete layer. The mononuclear cell layers were collected and washed twice with PBS. Finally, total RNA from the mononuclear cells was isolated for analysis.

### Bioinformatics analysis

Gene profiling data for AML cells were downloaded from the Cancer Cell Line Encyclopedia (https://sites.broadinstitute.org/ccle) database. AML cells were divided into TRAIL-sensitive (Sen) and TRAIL-resistant (Res) groups according to the IC_50_ against TRAIL. Differentially expressed genes (DEGs) were selected with |log2(fold-change)|> 1, *p* < 0.05 and other default parameters using the R package limma (version 3.60.2). Gene expression volcano plot and heatmap was plotted by a website (https://www.bioinformatics.com.cn), an online platform for visualization. Gene profiling data from 461 AML patients and 33 healthy donors were obtained from the Beat AML database (http://vizome.org/additional_figures_BeatAML.html). TP53INP2 mRNA levels were compared between groups.

### Colony formation assay

A methylcellulose clonogenic assay was carried out by seeding 1 × 10^3^ cells per well in triplicate in 24-well plates, and the cells were cultured in medium supplemented with methylcellulose and 20% fetal bovine serum (FBS) at 37 °C for 7 ~ 14 days. Colony-forming units, defined as cell clusters consisting of more than 5 cells, were counted using an inverted microscope (Nikon, Japan).

### Cell proliferation and apoptosis assays

Cell viability was assessed using the Cell Counting Kit-8 (CCK-8) Kit (Solarbio, Beijing, China). Cells were seeded in a 96-well plate at a density of 5 × 10^3^ ~ 1 × 10^4^/well. After adding 10 μL of CCK-8 solution, the cells were incubated for 2 ~ 3 h at 37 °C in the dark. Then, the absorbance was measured at 450 nm using a microplate reader (BioTek, CA, USA). The data are presented as the mean ± standard deviation (SD) of three independent experiments (*n* = 3). The IC_50_ of TRAIL was determined using GraphPad Prism 8.0.2 with log(inhibitor) vs. normalized responses.

Cell apoptosis was assessed using Annexin V FITC-PI staining assays. Briefly, cells were harvested and washed with PBS. Apoptosis staining was performed using an Annexin V FITC-PI Apoptosis Detection Kit (BD Biosciences, Piscataway, NJ, USA) according to the manufacturer’s instructions. The stained cells were analyzed using a FACSCalibur™ flow cytometer (FCM, BD Biosciences, USA) and CytExpert software (version 2.5). Cells that were incubated in drug-free medium and infected with shNC or vector plasmid served as the negative controls. The data are presented as the mean ± SD of three independent experiments (*n* = 3).

### Immunohistochemistry (IHC)

Cells were seeded on slides, treated with 3% H_2_O_2_ (ZSGB-BIO, Beijing, China) for 10 min, and blocked with 5% goat serum (ZSGB-BIO, Beijing, China) for 30 min. Then, the cells were incubated with primary antibodies at 4 °C overnight, treated with the corresponding secondary antibody for 1 h at room temperature, and stained with diaminobenzidine (ZSGB-BIO, Beijing, China). Finally, the cells were visualized by an inverted microscope (Nikon, Japan).

### RNA extraction and quantification

Total RNA was extracted using RNAiso Plus reagent (TaKaRa, Japan). cDNA was synthesized using a PrimeScript™ RT Reagent Kit (TaKaRa, Japan) according to the manufacturer’s protocol. TP53INP2 mRNA was amplified using 2 × SYBR Green Master Mix (TaKaRa, Japan). The levels of TP53INP2 mRNA were calculated by the 2^‐ΔΔCt^ method with β-actin as an internal control. The sequences of primers used were F: 5’-CCTCCCCTTCTCCTCCAGTAAA-3’ and R: 5’-AGCCCAAAATTCAGTCTCACCA-3’.

### Immunofluorescence (IF)

Cells grown on coverslips were fixed with 4% paraformaldehyde (Sigma‒Aldrich, USA) for 20 min, permeabilized with 0.3% Triton X-100 (Beyotime, China) for 15 min, and then blocked with 5% goat serum for 25 min at room temperature. Next, the cells were incubated at 4 °C overnight with primary antibodies, treated with Alexa Fluor 488‐ or 555‐conjugated secondary antibodies (Beyotime, China) for 1 h at room temperature, and treated with 4′,6-diamino-2-phenylindole (Beyotime, China) for nuclear counterstaining. Images were taken with a fluorescence microscope (Nikon, Japan). Quantification of fluorescence co-localization was performed using ImageJ software (version 1.8.0).

### Western blot

The cells were lysed in RIPA lysis buffer (Beyotime, China) containing protease inhibitor cocktails (Bimake, Houston, TX, USA). The proteins were separated by SDS‒PAGE and transferred to a polyvinylidene fluoride (PVDF) membrane (Bio-Rad, USA). The membranes were blocked with 5% nonfat milk for 1 h at room temperature and incubated with the indicated primary antibodies at 4 °C overnight. The following antibodies were used in the study: anti-TP53INP2 (LS-C743322, Lifespan Biosciences, USA), anti-NPM1-mA (PA1-46,356, Thermo Fisher Scientific, USA), anti-Caspase-3 (9662, Cell Signaling Technology, USA), anti-Caspase-8 (4927, Cell Signaling Technology, USA), anti-BID (2002, Cell Signaling Technology, USA), anti-DR4 (42,533, Cell Signaling Technology, USA), anti-DR5 (8074, Cell Signaling Technology, USA), anti-TRAF6 (8028, Cell Signaling Technology, USA), and anti-β-actin (20,536–1-AP, Proteintech, USA). After treatment with a secondary antibody (ZSGB-BIO, Beijing, China) for 1 h, the protein bands were visualized using an enhanced chemiluminescence (ECL) substrate (Bio-Rad, USA). Cells that were incubated in drug-free medium and infected with shNC or vector plasmid served as the negative controls. β-Actin was used as a loading control. Protein bands were quantified using ImageJ software.

### Coimmunoprecipitation (Co-IP)

Total protein from cells was extracted using IP lysis buffer (Beyotime, China), followed by centrifugation at 13,300 rpm at 4 °C for 30 min. Aliquots (1/10 volume) of the supernatant were analyzed by Western blot to assess protein expression using the indicated antibodies. The remaining supernatant was incubated with protein A/G beads (Bimake, USA) for immunoprecipitation with the indicated primary antibodies or normal immunoglobulin G (IgG) at 4 °C overnight. lgG with the same species and subtype as the primary antibody was used as the isotype control. The immunocomplexes were washed with PBS-T three times and boiled in 2 × SDS‒PAGE Sample Loading Buffer (Beyotime, China). The immunoprecipitated proteins were then analyzed by Western blotting.

### Ubiquitination assay

Cells were transfected with the indicated plasmids for 48 h and treated with 20 μM MG-132 for 8 h before harvesting. The cells were lysed on ice for 25 min using IP lysis buffer. The lysates were centrifuged at 13,300 rpm at 4 °C for 30 min. Using a portion of the supernatant, the expression of the indicated proteins was assessed by Western blotting. Then, the remaining supernatant was incubated with protein A/G beads. The mixture and primary antibodies or normal IgG were incubated at 4 °C overnight. lgG was used as the isotype control. The beads were washed three times with PBS-T buffer and boiled in 2 × SDS-PAGE sample loading buffer. The immunoprecipitated proteins were then analyzed by Western blotting with an anti-ubiquitin antibody (3936, Cell Signaling Technology, USA).

### AML xenograft mouse model

For the cell line-derived xenograft (CDX) model, NCG mice (5 to 6 weeks old, 8 individuals per group, female) were injected intravenously with 2 × 10^6^ OCI-AML3 or KG-1 cells carrying green fluorescent protein (GFP) and luciferase (Luc). Three weeks after injection, leukemia induction was confirmed via peripheral blood smear. Then, the mice were randomly divided into two groups and treated with TRAIL (5 mg/kg) or an equal amount of vehicle once every two days from week 4 to week 7 for 14 injections in total.

For patient-derived xenograft (PDX) models, 5 × 10^6^ freshly obtained bone marrow mononuclear cells from AML patient #3 with NPM1 mutation type A (NPM1-mA) or AML patient #2 with NPM1 wild type (wt) were injected into NCG mice through the tail vein to construct P1 PDX mice. After 5 weeks, P2 PDX mice were established by the intravenous injection of 2 × 10^6^ bone marrow mononuclear cells from P1 xenograft mice. Then, the P2 PDX mice were randomly injected with TRAIL (5 mg/kg), VEN (50 mg/kg), the TRAIL/VEN combination or vehicle. Mice in the vehicle group were used as controls. Body weight was measured daily. The drugs were administered once every two days for 4 weeks in total. Some of the mice in each group were sacrificed after the end of the 4th week of treatment; bone marrow, liver and spleen were obtained for flow analysis and H&E staining. The remaining mice in each group were monitored until death to generate Kaplan–Meier (K-M) curves. Human CD45^+^ cells from bone marrow were analyzed using a FACSCalibur™ flow cytometer (BD Biosciences). The number of immature bone marrow cells was evaluated via Wright’s staining (Solarbio, Beijing, China), and the infiltration of leukemic cells into the liver and spleen was analyzed via H&E staining (Solarbio, Beijing, China).

### Statistical analysis

Statistical analysis was conducted using GraphPad Prism (version 8.0.2) or SPSS (version 28.0.1). The normality of the data was determined using the Shapiro–Wilk test. Homogeneity of variance was analyzed using the Levene test. Normally distributed data with homogeneous variances are presented as the mean ± SD. Comparisons between two groups were conducted using unpaired Student’s t tests. Comparisons among multiple groups were evaluated using one-way ANOVA with Tukey’s post hoc test. Data with a nonnormal distribution or heterogeneous variances are presented as the median (interquartile range). Comparisons between two groups were conducted using the Mann–Whitney U test, and comparisons among multiple groups were conducted using the Kruskal–Wallis test. Correlations were examined using Pearson’s correlation coefficient. K-M estimation and the log-rank test were used to compare survival differences. *p* < 0.05 was considered to indicate statistical significance (^*^p < 0.05, ^**^p < 0.01, ^***^p < 0.001).

## Results

### The association between TP53INP2 and the sensitivity of AML cells to TRAIL treatment was identified by high-throughput library screening

**Fig. 1:**
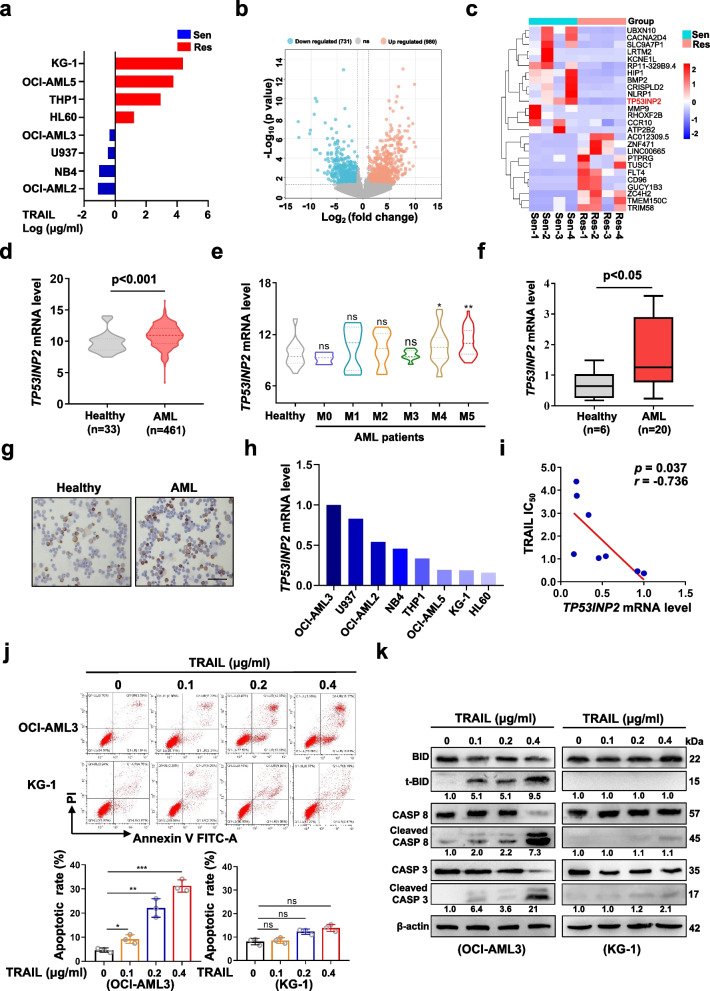
The association between TP53INP2 and the sensitivity of AML cells to TRAIL treatment was identified by high-throughput library screening. **a** The IC_50_ was estimated by CCK-8 with the concentration of TRAIL that resulted in 50% cell viability for 8 AML cells. The AML cells were divided into TRAIL-Sen and TRAIL-Res cells according to the IC_50_ against TRAIL. **b** Volcanic plot of the DEGs between TRAIL-Sen and TRAIL-Res AML cells. **c** Heatmap of the top 20 DEGs between TRAIL-Sen and TRAIL-Res AML cells. **d** Comparison of TP53INP2 mRNA levels in AML patients and healthy donors in Beat AML databases. **e** Comparison of TP53INP2 mRNA levels in various subtypes of AML patients in Beat AML databases. **f-g** qRT-PCR (**f**) and IHC staining (**g**) analyses of TP53INP2 levels in primary AML blasts and healthy donors (Scale bar: 50 μm). The upper, center, and lower line of the boxplot indicates 75%, 50%, and 25% quantile, respectively. Healthy donors served as controls in (d-g). **h** The expression of TP53INP2 in AML cells from the Cancer Cell Line Encyclopedia database. The ratio of TP53INP2/β-actin in OCI-AML3 cells was set to 1.0. **i** Pearson’s correlation analysis of TP53INP2 expression and the IC_50_ against TRAIL in AML cells. **j** FCM analysis (**j**, upper) and quantification (**j**, lower) of apoptotic cells in OCI-AML3 and KG-1 cells treated with 0–0.4 μg/ml TRAIL for 48 h**.** The cells incubated in drug-free medium served as controls. **k** Western blot analysis of the indicated apoptosis-related proteins. β-actin was used as a loading control. The quantification of protein levels was shown below the protein bands. The data are representative of at least three independent experiments. * *p* < 0.05, ** *p* < 0.01, *** *p* < 0.001, ns, not significant

To explore the potential mechanism underlying TRAIL resistance, we first evaluated the viability of AML cells exposed to different doses of TRAIL by CCK-8 assays. TRAIL-Sen and TRAIL-Res cells were divided according to the IC_50_ of TRAIL (Fig. 1a). Moreover, a total of 1,711 DEGs were identified from the Cancer Cell Line Encyclopedia database (Fig. 1b). Among the top 20 DEGs, TP53INP2 was identified as a candidate gene (Fig. 1c), as our previous study revealed that TP53INP2 plays a pivotal oncogenic role in AML [21]. Next, by analyzing datasets involving AML patients and healthy individuals from the Beat AML database, we found that TP53INP2 mRNA expression was considerably higher in AML patients, especially in AML-M4 and AML-M5 patients, than in healthy donors (Fig. 1d-e). The relatively high expression of TP53INP2 in primary AML blasts was subsequently confirmed by qRT-PCR (Fig. 1f), Western blot (Additional file 2: Figure S1a), and IHC (Fig. 1g) analyses. However, the TP53INP2 levels differed among the AML cell lines (Fig. 1h). Pearson’s correlation analysis revealed that TP53INP2 mRNA was negatively correlated with the IC_50_ of TRAIL (*r* = -0.736, *p* < 0.05; Fig. 1i). Next, the colony formation assay showed that TRAIL treatment reduced the number of colonies formed by OCI-AML3 cells but had little effect on the number of colonies formed by KG-1 cells (Additional file 2: Figure S1b). Consistent with these findings, TRAIL treatment significantly increased the cell apoptotic rate and the levels of the cell apoptosis-related proteins cleaved BID (t-BID), cleaved caspase-8 (CASP 8), and cleaved caspase-3 (CASP 3) and decreased the levels of BID, CASP 8 and CASP 3 in a concentration-dependent manner in OCI-AML3 cells, whereas few apoptotic KG-1 cells were observed (Fig. 1j-k). Moreover, there was no significant difference in DR4/5 expression between the two cell lines (Additional file 2: Figure S1c-e). These data suggested that TP53INP2 expression is positively correlated with the sensitivity of AML cells to TRAIL treatment.

### AML cells with high TP53INP2 expression were more sensitive to TRAIL in a CDX mouse model

**Fig. 2:**
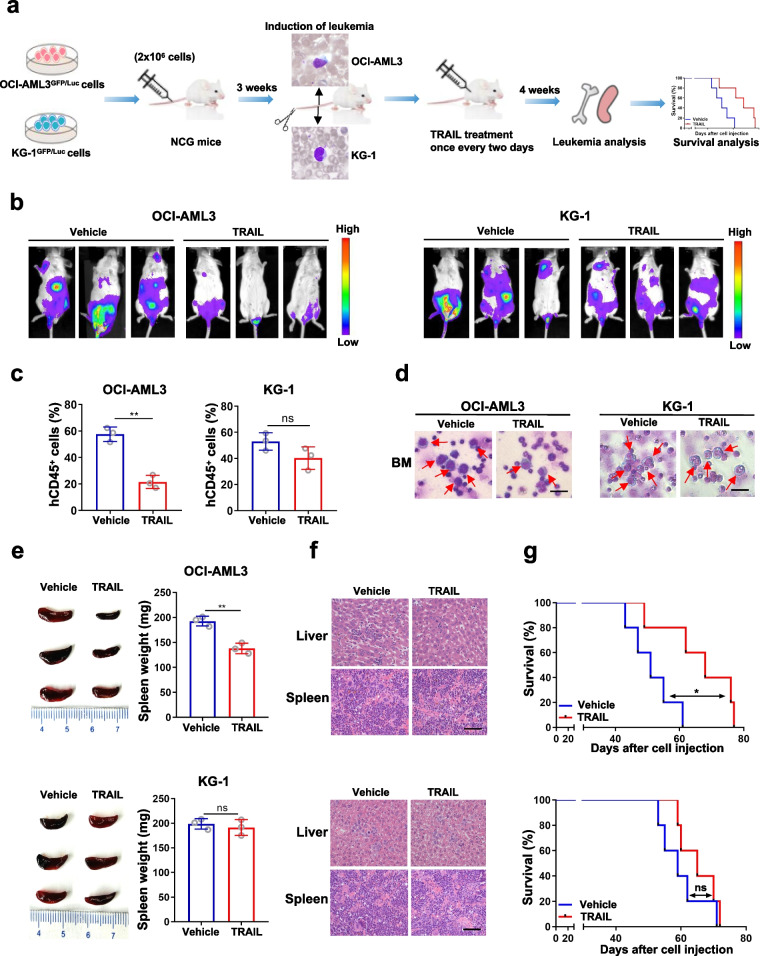
AML cells with high TP53INP2 expression were more sensitive to TRAIL in a CDX mouse model. **a** The schematic depiction of experiments in the CDX mouse models constructed with OCI-AML3 and KG-1 cells. **b** Representative images of bioluminescence in mice constructed with OCI-AML3 cells (**b**, left) and KG-1 cells (**b**, right). **c** FCM analyses (*n *= 3) of hCD45^+^ in OCI-AML3 cells (**c**, left) and KG-1 cells (**c**, right). **d** Wright’s staining of bone marrow immature cells (Scale bar: 100 μm). The red arrow indicates the immature cells. **e** Images of spleen appearance in mice with OCI-AML3 cells (**e**, upper) and KG-1 cells (**e**, lower).** f** H&E staining liver and spleen in mice with OCI-AML3 cells (**f**, upper) and KG-1 cells (**f**, lower) (Scale bar: 50 μm). **g** Kaplan–Meier survival curve of mice with OCI-AML3 cells (**g**, upper) and KG-1 cells (**g**, lower). The mice treated with an equal amount of vehicle were used as controls. The data are representative of at least three independent experiments. BM, bone marrow. * *p* < 0.05, ** p < 0.01, ns, not significant

To further investigate the relationship between TP53INP2 expression and TRAIL sensitivity in AML cells in vivo, a CDX mouse model was generated. Briefly, 2 × 10^6^ OCI-AML3 and KG-1 cells carrying GFP/Luc were injected into NCG mice via the tail vein. After confirmation of leukemia induction by peripheral blood smear, the mice were treated with TRAIL for 4 weeks (Fig. 2a). In mice injected with OCI-AML3 cells, TRAIL treatment significantly reduced the bioluminescence signal (Fig. 2b), the proportion of hCD45^+^ cells (Fig. 2c) and the number of leukemic cells (Fig. 2d) in bone marrow. Furthermore, TRAIL treatment resulted in decreased spleen weight (Fig. 2e). H&E staining revealed that fewer leukemia cells infiltrated the liver and spleen tissues (Fig. 2f). In line with these results, TRAIL treatment substantially prolonged the median survival of the mice (Fig. 2g). As shown in Fig. 2b-g, similar effects were not observed in the mice injected with KG-1 cells followed by TRAIL treatment. Collectively, these data demonstrated that AML cells with high TP53INP2 expression were more sensitive to TRAIL in vivo.

### TP53INP2 is a crucial regulator of the AML cell response to TRAIL treatment

**Fig. 3:**
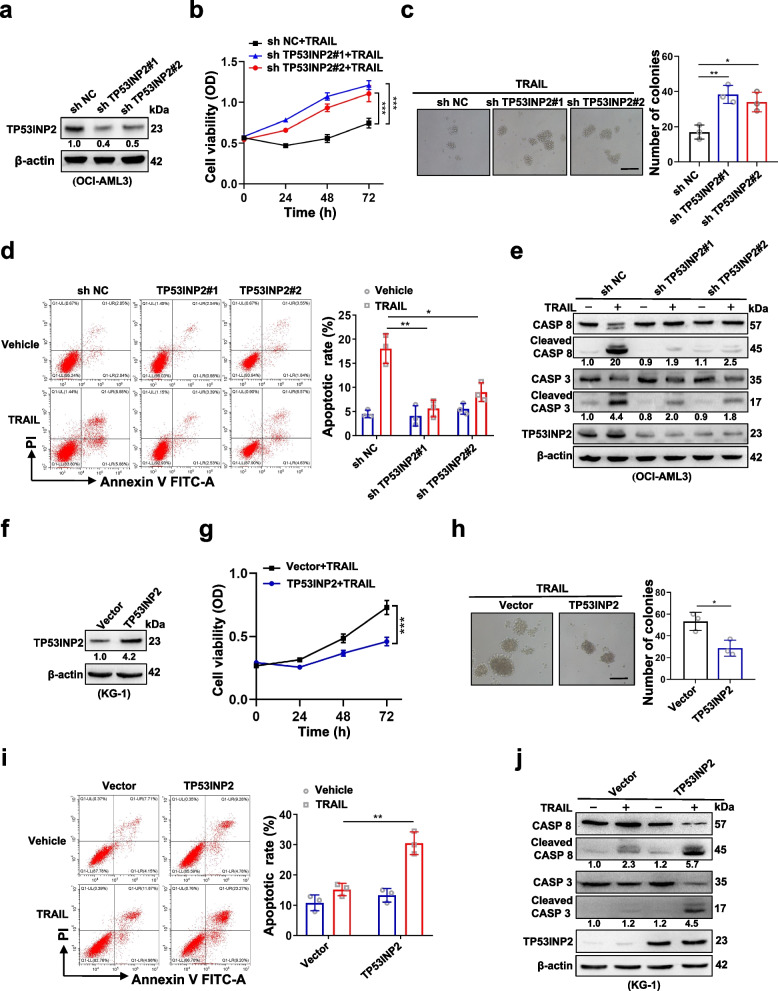
TP53INP2 is a crucial regulator of AML cell response to TRAIL treatment. **a** Western blot analysis of TP53INP2 in OCI-AML3 cells infected with two sh RNAs targeting TP53INP2.** b** CCK-8 analysis (n = 3) of cell viability in the OCI-AML3 cells treated with 100 ng/ml TRAIL for 0–72 h. **c** Colony formation assay was performed in the OCI-AML3 cells (Scale bar: 50 μm). The representative images and quantitative data from three independent experiments were shown in (**c**, left) and (**c**, right), respectively. **d** FCM analysis (**d**, left) and quantification (**d**, right) of apoptotic cells in the OCI-AML3 cells. **e** Western blot analysis of the indicated apoptosis-related proteins in the OCI-AML3 cells. The cells infected with sh NC (scramble shRNA) served as controls in (**a**-**e**). **f** Western blot analysis of TP53INP2 in KG-1 cells transfected with the HA-TP53INP2 plasmids. **g** CCK-8 analysis (*n* = 3) of cell viability in the KG-1 cells treated with 100 ng/ml TRAIL for 0–72 h. **h** Colony formation assay was performed in the KG-1 cells. The representative images and quantitative data from three independent experiments were shown in (**h**, left) and (**h**, right), respectively. (Scale bar: 50 μm). **i** FCM analysis (**i**, left) and quantification (**i**, right) of apoptotic cells in the KG-1 cells. **j** Western blot analysis of the indicated apoptosis-related proteins in the KG-1 cells. The cells transfected with vectors served as controls in (**f**-**j**). In (**a**, **e**,** f**, **j**), β-actin was used as a loading control, and the quantification of protein levels was shown below the protein bands. The data are representative of at least three independent experiments. * p < 0.05, ** *p* < 0.01, *** *p* < 0.001

To address the role of TP53INP2 in regulating the sensitivity of AML cells to TRAIL treatment, endogenous TP53INP2 was knocked down by a shTP53INP2 lentivirus in OCI-AML3 cells (Fig. 3a). TP53INP2 knockdown significantly reduced the vulnerability of the cells to TRAIL, as indicated by increased cell viability (Fig. 3b), increased number of colonies (Fig. 3c), decreased number of apoptotic cells (Fig. 3d) and decreased levels of cleaved CASP 8 and cleaved CASP 3 (Fig. 3e). Subsequently, the TP53INP2 protein was overexpressed in KG-1 cells (Fig. 3f). TP53INP2 overexpression increased the sensitivity of AML cells to TRAIL, decreasing cell viability (Fig. 3g), reducing the number of colonies (Fig. 3h), increasing the number of apoptotic cells (Fig. 3i) and increasing the levels of cleaved CASP 8 and cleaved CASP 3 (Fig. 3j). These findings indicated that TP53INP2 is a crucial regulator of AML cell sensitivity to TRAIL treatment.

### The effect of TP53INP2 on TRAIL sensitivity is regulated by mutant NPM1

**Fig. 4:**
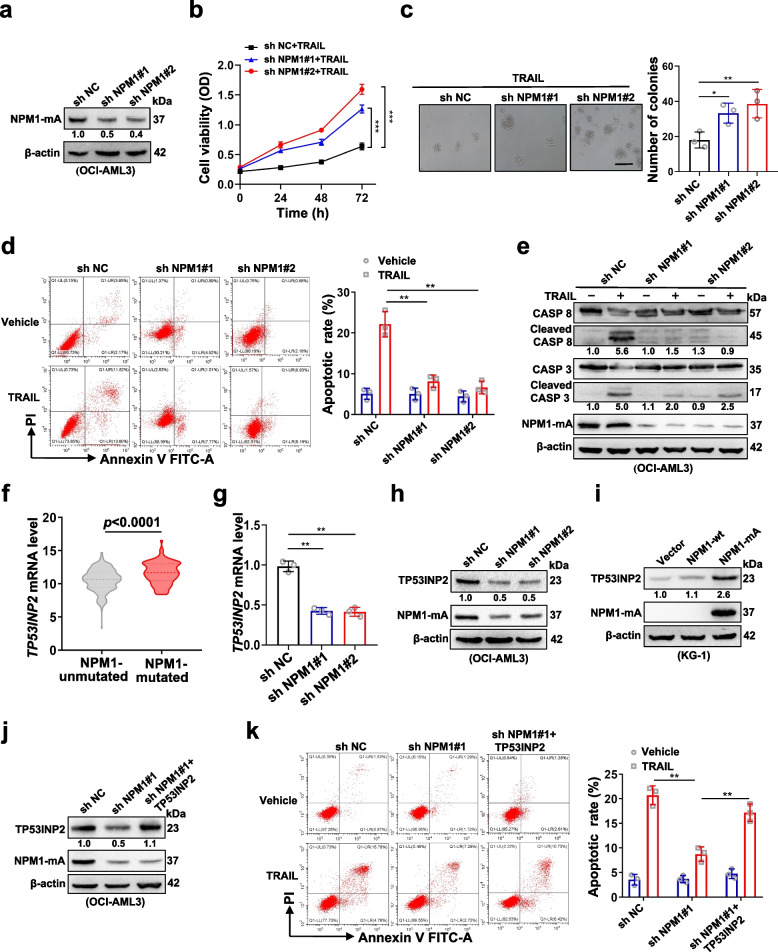
The effect of TP53INP2 on TRAIL sensitivity may be regulated by the mutant NPM1.** a** Western blot analysis of NPM1-mA in OCI-AML3 cells infected with two sh RNAs targeting NPM1.** b** CCK-8 analysis (*n* = 3) of cell viability in the cells treated with 100 ng/ml TRAIL for 0–72 h. **c** Colony formation assay was performed in OCI-AML3 cells. The representative images and quantitative data from three independent experiments were shown in (**c**, left) and (**c**, right), respectively. (Scale bar: 50 μm). **d** FCM analysis (**d**, left) and quantification (**d**, right) of apoptotic cells in the cells. **e** Western blot analysis of the indicated apoptosis-related proteins. **f** TP53INP2 levels in NPM1-mutated AML patients, compared with NPM1-unmutated AML patients, were identified from Beat AML databases.** g** qRT-PCR analysis of *TP53INP2* mRNA levels in NPM1-mA-silenced OCI-AML3. **h** Western blot analysis of TP53INP2 and NPM1-mA in OCI-AML3 cells with two sh NPM1. **i** Western blot analysis of TP53INP2 and NPM1-mA in KG-1 cells with the NPM1-wt and NPM1-mA plasmids. **j** Western blot analysis of TP53INP2 and NPM1-mA. In (**a**,** e**, **h**-**g**), β-actin was used as a loading control, and the quantification of protein levels was shown below the protein bands. **k** FCM analysis (**k**, left) and quantification (**k**, right) of apoptotic cells in OCI-AML3 cells with sh NPM1 before transfection with the HA-TP53INP2 plasmids. The cells transfected with shNC or vectors served as controls. The data are representative of at least three independent experiments. * p < 0.05, ** *p* < 0.01, *** *p* < 0.001

Given that OCI-AML3 cells naturally carrying NPM1-mA exhibit increased TP53INP2 levels and increased sensitivity to TRAIL treatment and that NPM1 mutations are recognized as AML-initiating lesions [22], we investigated whether NPM1-mA is involved in regulating the effect of TP53INP2 on TRAIL sensitivity. OCI-AML3 cells with stable NPM1 knockdown were successfully generated (Fig. 4a). NPM1-mA depletion significantly reduced the vulnerability of the cells to TRAIL (Fig. 4b), increased the number of colonies (Fig. 4c), decreased the number of apoptotic cells (Fig. 4d), and decreased the levels of cleaved CASP 8 and cleaved CASP 3 (Fig. 4e). Subsequently, the NPM1-mA and NPM1-wt plasmids were transfected into KG-1 cells (Additional file 3: Figure S2a). We found that the expression of NPM1-mA, but not that of NPM1-wt, increased the sensitivity of AML cells to TRAIL (Additional file 3: Figure S2b-e). Notably, elevated TP53INP2 was also detected in NPM1-mutated AML samples in the Beat AML datasets (Fig. 4f). The silencing of NPM1-mA reduced the TP53INP2 level (Fig. 4g-h), while NPM1-mA overexpression resulted in the upregulation of TP53INP2 expression (Fig. 4i). Furthermore, a rescue experiment revealed that TP53INP2 overexpression partially reversed the reduction in TRAIL-induced apoptosis caused by NPM1-mA knockdown (Fig. 4j-k). Our results support the notion that mutant NPM1 contributes to the effect of TP53INP2 on TRAIL sensitivity.

### Cytoplasmic TP53INP2 maintained by mutant NPM1 functions as a scaffold linking TRAF6 and CASP 8 to promote the ubiquitination and activation of CASP 8

**Fig. 5:**
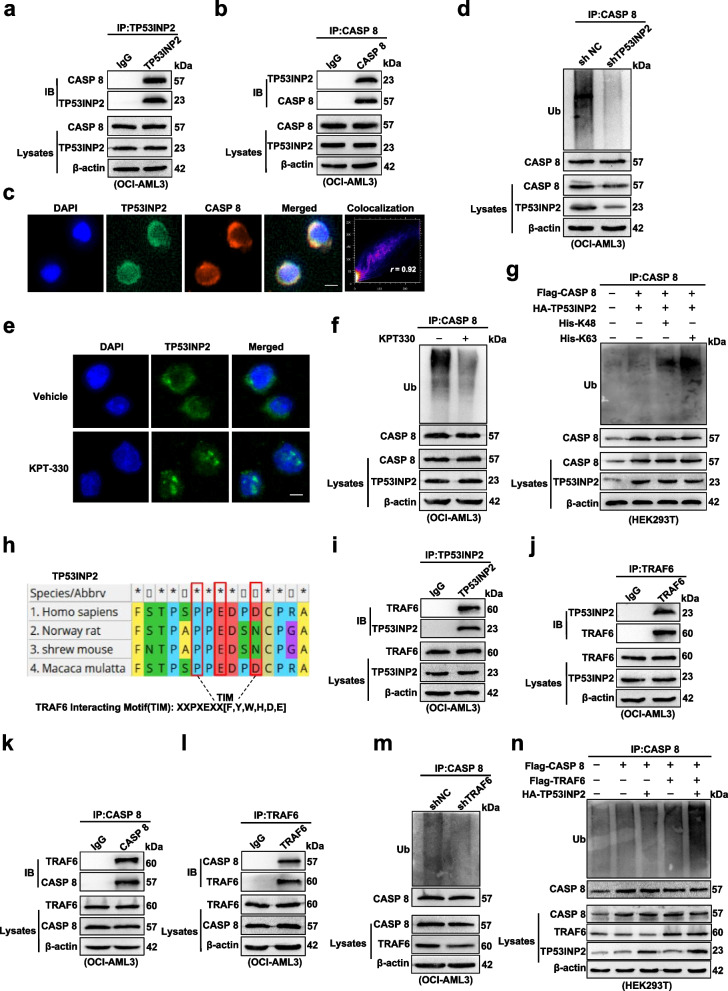
Cytoplasmic TP53INP2 maintained by mutant NPM1 functions as a scaffold linking TRAF6 and CASP 8 to promote the ubiquitination activation of CASP 8. **a-b** Co-IP analysis of CASP 8 and TP53INP2 in OCI-AML3 cells. **c** IF analysis of endogenous TP53INP2 (green) and CASP 8 (red) co-localization in OCI-AML3 cells (Scale bar: 5 µm). Quantification of fluorescence co-localization was performed using ImageJ software. *r* = 0.92 indicated that the two proteins were co-located. **d** Ubiquitination analysis of the ubiquitinated CASP 8 level in the shTP53INP2 OCI-AML3 cells. **e** IF analysis of TP53INP2 localization in OCI-AML3 cells treated with 2 µM KPT-330 for 10 h (Scale bar: 5 µm). **f** Ubiquitination analysis of the ubiquitinated CASP 8 level in OCI-AML3 cells treated with 2 µM KPT-330 for 10 h. **g** Ubiquitination assay of the ubiquitinated CASP 8 level in HEK293T cells co-transfected with HA-TP53INP2, Flag-CASP 8, His-Ub-K48 and His-Ub-K63. **h** Schematic presentation of the TRAF6 interaction motif in TP53INP2 protein. **i-j** Co-IP analysis of TP53INP2 and TRAF6 in OCI-AML3 cells. **k-l** Co-IP assay of CASP 8 and TRAF6 in OCI-AML3 cells. **m** Ubiquitination analysis of the ubiquitinated CASP 8 level in the TRAF6-knockdown OCI-AML3 cells. **n** Ubiquitination assay of the ubiquitinated CASP 8 level in HEK293T cells co-transfected with Flag-CASP 8, Flag-TRAF6 and HA-TP53INP2. IgG was used as an isotype control in (**a**-**b**, **i**-**l**)

Next, we investigated the potential mechanism underlying how ectopic TP53INP2 contributes to TRAIL-induced apoptosis. The ubiquitination-mediated activation of CASP 8 is an initiator of extrinsic apoptosis signaling in cancer cells [23]. We explored whether TP53INP2 participates in the regulation of CASP 8 activation upon TRAIL treatment. First, the interaction between endogenous TP53INP2 and CASP 8 was assessed by co-IP analysis (Fig. 5a-b). The co-localization of TP53INP2 and CASP 8 in the cytoplasm of OCI-AML3 cells was analyzed by IF, and the fluorescence intensity was further quantified using ImageJ software (Fig. 5c, *r *= 0.92, which indicates that the two proteins were colocalized). Second, ubiquitination experiments revealed that TP53INP2 knockdown reduced the ubiquitination of CASP 8 (Fig. 5d). Considering the important role of cytoplasmic TP53INP2 maintained by NPM1-mA in AML cells [21], we further tested whether the subcellular localization of TP53INP2 influences CASP 8 ubiquitination. KPT-330, a selective CRM-1/exportin-1 (XPO-1) inhibitor, was used to relocalize cytoplasmic NPM1-mA and its partner TP53INP2 to the nucleus (Fig. 5e and Additional file 4: Figure S3a), resulting in a decrease in the level of ubiquitinated CASP 8 (Fig. 5f). Ubiquitin chain linkages such as K48 and K63 have been used for CASP 8 ubiquitination [24, 25]. Therefore, we explored whether K48- or K63-linked ubiquitination of CASP 8 occurs via TP53INP2. As shown in Fig. 5g, TP53INP2 increased the K63 but not the K48-linked ubiquitination of CASP 8. Third, we sought to determine the E3 ubiquitin ligase responsible for inducing the ubiquitination of CASP 8 in the presence of TP53INP2. A TRAF6-interacting motif in the TP53INP2 protein was mapped using Molecular Evolutionary Genetics Analysis (MEGA, https://www.megasoftware.net/) (Fig. 5h). The co-IP and IF results confirmed that TP53INP2 interacted with TRAF6 (Fig. 5i-j and Additional file 4: Figure S3b) and that TRAF6 also interacted with CASP 8 (Fig. 5k-l and Additional file 4: Figure S3c) in OCI-AML3 cells, indicating that TP53INP2, CASP 8 and TRAF6 may be located in the same complex (Additional file 4: Figure S3d). TRAF6 deficiency decreased CASP 8 ubiquitination (Fig. 5m). In addition, TRAF6-mediated CASP 8 ubiquitination was enhanced by TP53INP2 expression (Fig. 5n). Taken together, these data indicated that cytoplasmic TP53INP2 acts as a scaffold to promote the ubiquitination and activation of CASP 8 in a TRAF6-dependent manner.

### TRAIL shows synergistic effect in combination with the BCL-2 inhibitor venetoclax in the presence of TP53INP2 in vitro

**Fig. 6:**
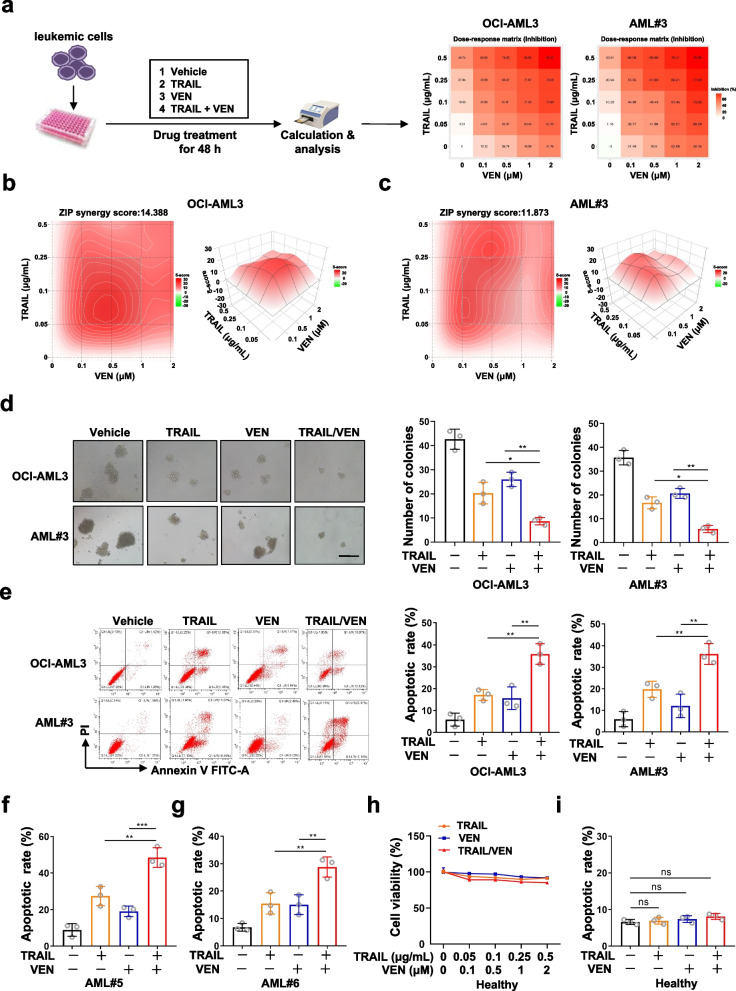
TRAIL shows a synergistic effect in combination with the BCL-2 inhibitor venetoclax in the presence of TP53INP2 in vitro. **a** The schematic depiction of experiments about drug treatment. **b-c** CCK-8 analysis (*n* = 3) of cell viability in the OCI-AML3 cells **(b)** and primary blasts **(c)** treated with TRAIL and VEN, alone or incombination for 48 h. SynergyFinder was used to evaluate the ZIP synergy score.** d** Colony formation assay was performed in the OCI-AML3 and AML#3 cells (Scale bar: 50 μm). The representative images and quantitative data from three independent experiments were shown in (**d**, left) and (**d**, right), respectively. **e** FCM analysis (**e**, left) and quantification (**e**, right) of apoptotic cells in the OCI-AML3 and AML#3 cells treated with TRAIL and VEN, alone or in combination for 48 h. **f**-**g** Quantification of apoptotic cells by FCM (*n* = 3) in the AML#5 cells (**f**) and AML#6 cells (**g**) treated with TRAIL and VEN, alone or in combination for 48 h. **h-i** CCK-8 assay (*n* = 3) of cell viability (**h**) and quantification of apoptotic cells (**i**) in the mononuclear cells from healthy donors. The cells incubated in a drug-free medium served as controls. The data are representative of at least three independent experiments. * *p* < 0.05, ** *p* < 0.01, *** *p* < 0.001, ns, not significant

Based on the evidence of TRAIL-mediated CASP 8-dependent extrinsic apoptosis in AML cells with highTP53INP2 expression, we evaluated whether the addition of the BCL-2 inhibitor venetoclax (VEN) enhanced TRAIL sensitivity in AML cells in vitro*.* First, TRAIL and VEN alone or in combination were added to OCI-AML3 cells and primary blasts with high TP53INP2 expression, and synergistic inhibitory scores for TRAIL in combination with VEN were calculated using a dose–response matrix with SynergyFinder (https://synergyfinder.fimm.fi) (Fig. 6a). Notably, the synergy scores for the combination treatment were calculated by applying the zero interaction potency (ZIP) independence model in SynergyFinder, with a ZIP synergy score of 14.388 for OCI-AML3 cells and 11.873 for primary blasts (Fig. 6b-c, Zip scores less than -10 suggest an antagonistic effect, while scores greater than 10 suggest synergy). Moreover, compared to TRAIL or VEN alone, the TRAIL/VEN combination substantially reduced the number of colonies (Fig. 6d) and increased the number of AML apoptotic cells (Fig. 6e-g), with no significant effect on normal blood cells from healthy donors (Fig. 6h-i). These data indicated that the combination of TRAIL and VEN in the presence of TP53INP2 had a synergistic effect in vitro.

### The TRAIL/venetoclax combination reduces tumor burden and prolongs survival in a PDX mouse model of AML

**Fig. 7:**
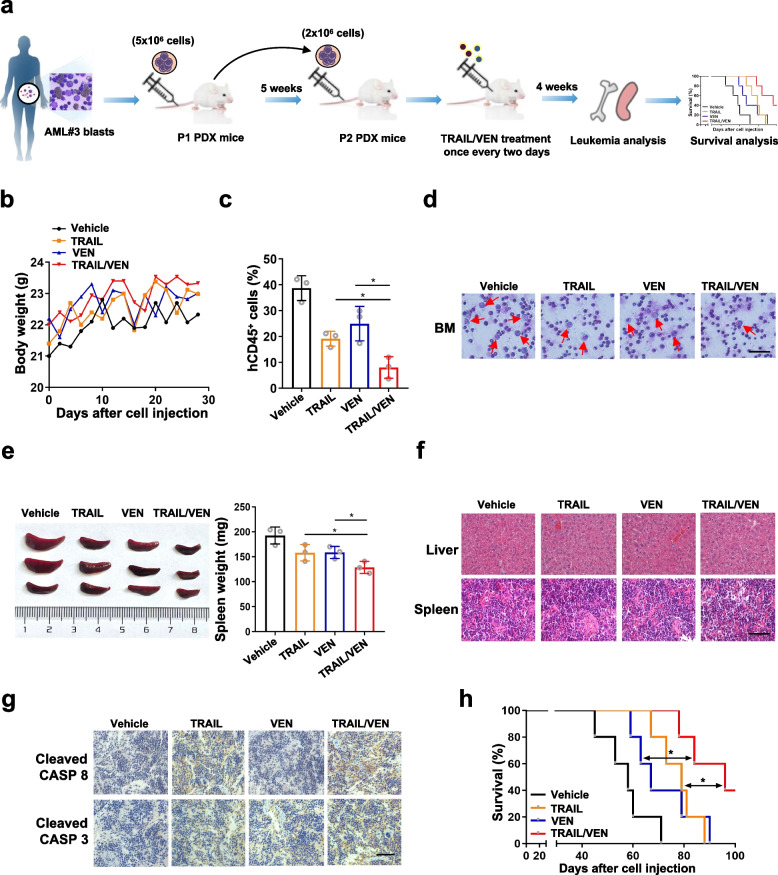
The TRAIL/venetoclax combination reduces tumor burden and prolongs survival in a PDX mouse model of AML. **a** The schematic depiction of experiments about the PDX mouse model constructed with bone marrow leukemic cells from AML#3 patient with NPM1 mutant. **b** The weights of all the mice were recorded during the experiment.** c** Quantification of hCD45.^+^ leukemic cells from PDX mice by FCM (*n* = 3).** d** Wright’s staining of bone marrow immature cells (Scale bar: 100 μm). The red arrow indicates the immature cells. **e** Images of spleen appearance.** f** H&E staining of liver (**f**, upper) and spleen (**f**, lower) from one representative mouse in each group (Scale bar: 50 μm).** g** IHC staining of cleaved CASP 8 (**g**, upper) and cleaved CASP 3 (**g**, lower) expression in the spleen. **h** Kaplan–Meier survival curve of mice in each group. The mice treated with an equal amount of vehicle were used as controls. The data are representative of at least three independent experiments. BM, bone marrow. * p < 0.05

To determine whether TRAIL and VEN alone or in combination are effective against AML cells expressing TP53INP2 in vivo, a PDX mouse model was generated by injecting mice with leukemic cells from AML patient #3 carrying NPM1-mA. After leukemia induction was confirmed by a peripheral blood smear, the mice were treated with TRAIL and/or VEN for 4 weeks (Fig. 7a). The weights of all the mice were measured before and after drug treatment (Fig. 7b). The TRAIL/VEN combination considerably decreased the proportion of hCD45^+^ cells (Fig. 7c) and the number of leukemic blasts in bone marrow (Fig. 7d). In addition, the TRAIL/VEN combination treatment effectively reduced the weight of the spleen (Fig. 7e), and leukemic cells infiltrated the liver and spleen (Fig. 7f). IHC analyses showed that the TRAIL/VEN combination increased the expression levels of cleaved CASP 8 and cleaved CASP 3 (Fig. 7g). Most importantly, mice that received the TRAIL/VEN combination treatment had a longer median survival than did those that received the vehicle or a single drug (Fig. 7h). Notably, similar effects were not observed on a PDX mouse model generated from AML patient #2 with NPM1 wt followed by the TRAIL/VEN combination treatment (Additional file 5: Figure S4a-g). Collectively, these results demonstrated that the TRAIL/VEN combination regimen is highly effective in a NPM1 mutant PDX mouse model.

## Discussion

**Fig. 8:**
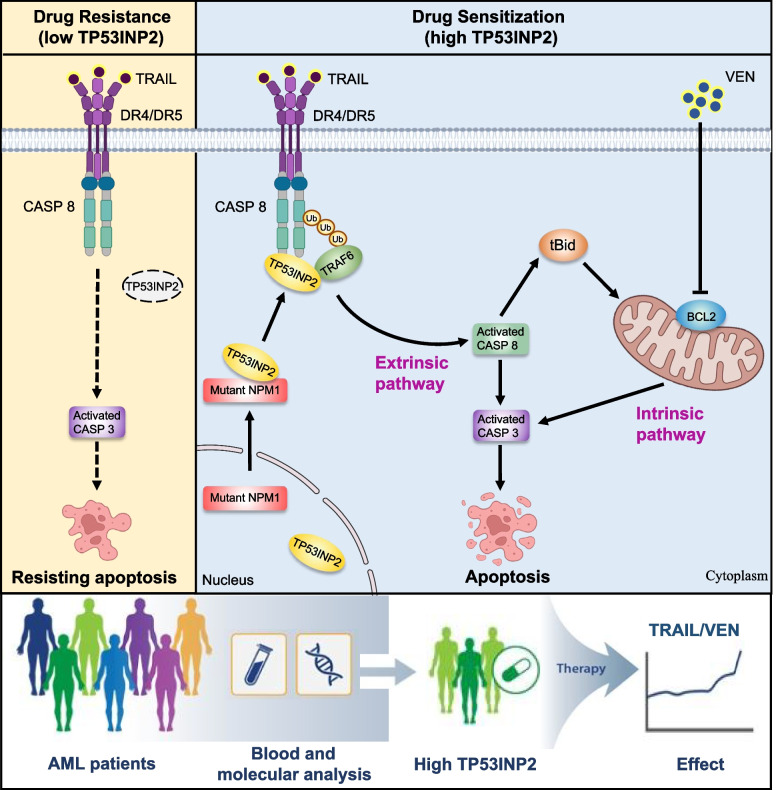
Schematic model illustrating the role of TP53INP2 in potentiating TRAIL-mediated apoptosis. The AML cells with high TP53INP2 expression were more sensitive to TRAIL. Mechanically, mutant NPM1 enhances the cytoplasmic distribution of TP53INP2, TP53INP2 functions as a scaffold bridging the ubiquitin ligase TRAF6 to CASP 8, thereby promoting the ubiquitination activation of CASP 8. Simultaneously stimulating extrinsic and intrinsic apoptosis-signaling pathways with TRAIL and VEN synergistically promotes apoptosis of AML cells. Therefore, TP53INP2 may serve as a potential predictive biomarker for the selection of AML patients for TRAIL alone or in combination with VEN therapy

Although TRAIL induces selective apoptosis in cancer cells, the clinical application of TRAIL has been rather limited [26–28]. Exploring suitable biomarkers for the selection of patients responsive to TRAIL is pivotal for devising rational individualized therapy regimens. Herein, we demonstrated that AML cells with high TP53INP2 expression were more sensitive to TRAIL and that cytoplasmic TP53INP2 modulated by mutant NPM1 functioned as a scaffold linking the ubiquitin ligase TRAF6 and CASP 8 to promote the ubiquitination and activation of CASP 8. On this basis, simultaneously stimulating extrinsic and intrinsic apoptosis signaling pathways with TRAIL and the BCL-2 inhibitor VEN resulted in synergistic antileukemic activity (Fig. 8).

TP53INP2, defined as an autophagic protein, promotes autophagosomal formation and activates autophagic flux in different cell lines [29, 30]. We previously reported that TP53INP2 plays a critical oncogenic role in AML [21]. A recent study showed that high levels of TP53INP2 are correlated with TRAIL-triggered cell death in breast cancer [20]. Consistent with those findings, the results of our study indicated that AML cells with high TP53INP2 expression were more sensitive to TRAIL in vitro and in CDX mouse models and that the suppression of TP53INP2 expression significantly reduced vulnerability to TRAIL. Our study is the first to demonstrate that TP53INP2 has the same effect on regulating the TRAIL response in AML.

Numerous studies have focused on the mechanisms involved in the regulation of TRAIL-mediated cell death pathways. Cho DH and colleagues [31] reported that ATG6 is a substrate for caspases and that the downregulation of ATG6 expression sensitizes HeLa cells to TRAIL-triggered cell death. In another study, X-linked inhibitor of apoptosis protein (XIAP) was highly expressed in various leukemia cells, and the inhibition of XIAP restored TRAIL sensitivity in resistant cells and primary blasts [32]. Herein, we revealed an unexpected function of TP53INP2 as an apoptosis partner in regulating TRAIL efficacy in the treatment of AML. Notably, we observed that OCI-AML3 cells that naturally carried NPM1-mA exhibited greater TP53INP2 levels and greater sensitivity to TRAIL treatment than did other AML cells, prompting us to speculate whether NPM1-mA is involved in regulating the effect of TP53INP2 on TRAIL activity. As anticipated, NPM1-mA depletion reduced TRAIL susceptibility, an effect that was partially reversed by the overexpression of TP53INP2, indicating that NPM1-mA is potentially upstream of TP53INP2 in the regulation of TRAIL activity. However, the association between TP53INP2 and TRAIL activity should be further validated in clinical samples from AML patients with NPM1 mutations.

Next, we focused on the molecular mechanism underlying TP53INP2-regulated TRAIL sensitivity in AML. Ubiquitination and activation of CASP 8 is an initiator of TRAIL-induced extrinsic apoptosis signaling in cancer cells [33]. In the present study, TP53INP2 colocalized with CASP 8 in the cytoplasm and promoted K63 ubiquitination, suggesting that TP53INP2 participates directly in CASP 8 activation in AML cells. Indeed, TP53INP2 is a multifunctional protein whose subcellular localization is a key determinant of its functional switching [34]. A previous study demonstrated that when nutrients are depleted, MTORC1 is the master upstream regulator controlling the distribution of TP53INP2 in the cytoplasm [35]. Similarly, TGF-β2 can facilitate TP53INP2 translocation from the nucleus to the cytoplasm in human lens epithelial cells [36]. Our previous data revealed that NPM1-mA maintains the cytoplasmic localization of TP53INP2 in AML cells. In the present study, KPT-330, a nuclear export inhibitor, was used to relocalize cytoplasmic NPM1-mA and its partner TP53INP2 to the nucleus, resulting in a decrease in the level of ubiquitinated CASP 8. In addition, we further explored the molecular mechanism by which TP53INP2 regulates CASP 8 ubiquitination. Notably, TP53INP2 is not an E3 ubiquitin ligase, which prompted us to subsequently identify which E3 ubiquitin ligase is responsible for the ubiquitination of CASP 8. Given our earlier findings that the E3 ubiquitin ligase TRAF6 mediates the K63 ubiquitination of ULK1 in leukemia cells [37] and the results of MEGA analysis in this work showing a TRAF6-interacting motif in the TP53INP2 protein, we analyzed the potential regulatory effect of TRAF6 on CASP 8 ubiquitination in the presence of TP53INP2. The results of our experiments confirmed that TP53INP2, TRAF6, and CASP 8 interact with each other and that TP53INP2 enhances TRAF6-mediated CASP 8 ubiquitination. These observations suggest that TP53INP2 could act as a scaffold protein bridging TRAF6 to CASP 8 to promote the ubiquitination and activation of CASP 8 in AML cells. This finding was supported by recent evidence showing that cytoplasmic TP53INP2 contributes to autophagosome biogenesis by acting as a scaffold mediating the LC3-ATG7 protein interaction in HeLa cells [38]. In fact, in addition to TRAF6, two more E3 ubiquitin ligases, HECTD3 [39] and cullin-3 [40], have been reported to promote the ubiquitination of CASP 8 during death receptor-induced apoptosis. Thus, further studies are needed to explore the significance of other E3 ubiquitin ligases for CASP 8 ubiquitination in AML.

Apoptosis can be triggered through either an intrinsic or extrinsic pathway [41]. Accumulating evidence has corroborated that concurrently targeting the two apoptosis signaling pathways may lead to more durable antitumor activity [42]. VEN, a selective inhibitor of BCL-2 that effectively induces the intrinsic apoptosis of tumor cells, has attracted considerable attention [43–45]. Recently, VEN-based therapies were approved by the Food and Drug Administration (FDA) for the treatment of AML patients who cannot receive intensive chemotherapy or who are older than 75 years of age [46, 47]. Numerous studies have shown that the antileukemic activity of VEN is limited to combination therapies [48, 49]. In this work, the combinatorial activity of TRAIL and VEN was assessed in AML cell lines and patient samples with high levels of TP53INP2, and this activity was validated in a PDX mouse model. In clinical practice, TP53INP2 is an independent predictor of disease prognosis in AML [21]. Thus, it would be interesting to explore the expression of TP53INP2 in AML cells for the selection of AML patients for TRAIL alone or in combination with VEN therapy. A recent study reported that combination therapy with the TRAIL receptor agonist eftoza and VEN enhanced TRAIL receptor activity in AML cell lines and patient-derived ex vivo/in vivo models [50]. Encouragingly, the eftoza/VEN combination therapy in patients with relapsed/refractory AML showed more-durable antileukemic activity in a phase I clinical trial. In combination cohorts, the overall response rate (ORR) was 30% with median duration of response > 7 months; the objective response rate was 26%, with 17% achieving a complete response (CR) and 9% achieving a CR with incomplete blood count recovery (CRi). As a comparison, venetoclax therapy in a similar setting reported 19% CR/CRi rate with median duration of response < 4 months [51]. In addition, treatment with eftoza/VEN combination therapy was acceptably tolerated in these patients, which aligned with those expected from VEN therapy [50]. In another study, the authors revealed that the TRAIL-mediated extrinsic apoptotic pathway is a target for overcoming VEN resistance [52]. From a clinical point of view, the TRAIL/VEN combination may be potentially efficacious, supporting further clinical evaluations of this combination in TP53INP2-positive AML patients. Notably, the specific mechanisms of this combination need to be verified in future studies.

## Conclusion

In summary, our results revealed an unexpected function of TP53INP2 as an apoptosis partner in the regulation of TRAIL activity in AML, especially in AML cells with NPM1 mutations. Importantly, this finding indicates that TP53INP2 might serve as a biomarker to predict AML patient response to TRAIL therapy and provides a reasonable basis for devising individualized therapy regimens.

### Supplementary Information

Additional file 1: Table S1. Clinical characteristics of primary AML patient samples.

Additional file 2: Figure S1. The expression of TP53INP2 is positively correlated with the sensitivity of AML cells to TRAIL treatment. a Western blot analysis of TP53INP2 levels in primary AML blasts and healthy donors. β-actin was used as a loading control, and the quantification of protein levels was shown below the protein bands. b Colony formation assay was performed in the OCI-AML3 (b, left and upper) and KG-1 cells (b, left and lower) treated with 0-0.4 μg/ml TRAIL for 48 h (Scale bar: 50 μm). The representative images and quantitative data from three independent experiments were shown in (b, middle) and (b, right), respectively. The cells incubated in a drug-free medium served as controls. c-e Western blot (c), FCM (d), and IF (e) analyses of DR4 and DR5 levels in the OCI-AML3 and KG-1 cells. The data are representative of at least three independent experiments. * *p*<0.05, ** *p*<0.01, ns, not significant.

Additional file 3: Figure S2. NPM1-mA expression, but not the NPM1-wt, rendered AML cells more sensitive to TRAIL. a Western blot analysis of NPM1-mA in KG-1 cells transfected with the NPM1-wt and NPM1-mA plasmids. b CCK-8 analysis (*n* =3) of cell viability in the cells treated with 100 ng/ml TRAIL for 0-72 h. c Colony formation assay was performed in KG-1 cells (Scale bar: 50 μm). The representative images and quantitative data from three independent experiments were shown in (c, left) and (c, right), respectively. d FCM analysis (d, left) and quantification (d, right) of apoptotic cells in the cells. The cells transfected with vector plasmid served as controls. e Western blot analysis of the indicated apoptosis-related proteins. In (a, e), β-actin was used as a loading control, and the quantification of protein levels was shown below the protein bands. The data are representative of at least three independent experiments. * *p*<0.05, ** *p*<0.01, ns, not significant.

Additional file 4: Figure S3. Cytoplasmic TP53INP2 functions as a scaffold linking TRAF6 with CASP 8. a Western blot analysis of TP53INP2 and NPM1-mA levels in the cytoplasm and nucleus of OCI-AML3 cells treated with 2 µM KPT-330 for 10 h. GAPDH was used as a loading control in the cytoplasm and Histone was used as a loading control in the nucleus. b IF analysis of endogenous TP53INP2 (green) and TRAF6 (red) co-localization in OCI-AML3 cells (Scale bar: 5 µm). c IF analysis of endogenous CASP 8 (green) and TRAF6 (red) co-localization in OCI-AML3 cells (Scale bar: 5 µm). Quantification of fluorescence co-localization was performed using Image J software, and r of 0.5~1.0 means that the two proteins are co-located in (b-c). d The schematic depiction of TP53INP2 linking TRAF6 and CASP 8 to promote the ubiquitination activation of CASP 8.

Additional file 5: Figure S4. The effect of TRAIL/VEN combination on PDX generated with NMP1 wt AML. a The weights of all the mice were recorded during the experiment. b Quantification of hCD45+ leukemic cells from PDX mice by FCM (*n*=3). c Wright’s staining of bone marrow immature cells (Scale bar: 100 μm). The red arrow indicates the immature cells. d Images of spleen appearance. e H&E staining of liver (e, upper) and spleen (e, lower) from one representative mouse in each group (Scale bar: 50μm). f IHC staining of cleaved CASP 8 (f, upper) and cleaved CASP 3 (f, lower) expression in the spleen. g Kaplan-Meier survival curve of mice in each group. The mice treated with an equal amount of vehicle were used as controls. The data are representative of at least three independent experiments. BM, bone marrow. ns, not significant.

Additional file 6: The STR profile reports for AML cell lines.

Additional file 7: The full (not cropped) Western blot gel figures.

## Data Availability

All data generated or analyzed during this study are included in this published article [and its supplementary information files].

## Abbreviations

AML: Acute myeloid leukemia
CASP 8: Caspase-8
CCK-8: Cell Counting Kit-8
CDX: Cell line-derived xenograft
CHKA: Choline kinase alpha
Co-IP: Co-immunoprecipitation
DEG: Differentially expressed gene
FCM: Flow cytometry
GFP: Green fluorescent protein
hCD45^+^: Human-CD45^+^
H&E: Hematoxylin and eosin
IF: Immunofluorescence
IHC: Immunohistochemistry
Luc: Luciferase
NPM1: Nucleophosmin 1
PBS: Phosphate buffered saline
PDX: Patient-derived xenograft
PVDF: Polyvinylidene fluoride
SD: Standard deviation
sh RNA: Lentivirus-based short hairpin RNA
TNF: Tumor necrosis factor
TP53INP2: Tumor protein p53 inducible nuclear protein 2
TRAF6: TNF receptor associated factor 6
TRAIL: Tumor necrosis factor-related apoptosis-inducing ligand
VEN: Venetoclax
XIAP: X-linked inhibitor of apoptosis protein
