# Supplementary material for: Cytoplasmic TP53INP2 acts as an apoptosis partner in TRAIL treatment: the synergistic effect of TRAIL with venetoclax in TP53INP2-positive acute myeloid leukemia
Source: J Exp Clin Cancer Res. 2024 Jun 22;43:176. doi: 10.1186/s13046-024-03100-0 (PMC11193246; doi:10.1186/s13046-024-03100-0)
Supplement: Supplementary file 1 — Additional file 1: Table S1. Clinical characteristics of primary AML patient samples. [file 13046_2024_3100_MOESM1_ESM.pdf]

Additional file 1: Table S1. Clinical characteristics of primary AML patient samples.

Patient	Gender	Age (year)	Disease status	FAB subtype	Blast purity (%)	Gene mutation
AML#1	Male	81	Newly diagnosed	M5	6.39	CEBPA
AML#2	Female	28	Newly diagnosed	M2	10.44	WT1
AML#3	Female	55	Newly diagnosed	M4	92.5	NPM1, FLT3-ITD
AML#4	Male	66	Newly diagnosed	M2	16.63	c-KIT
AML#5	Female	49	Newly diagnosed	M4	77.1	NPM1,DNMT3A
AML#6	Male	72	Newly diagnosed	M5	62.0	NPM1, WT1
AML#7	Male	19	Newly diagnosed	M3	19.42	FLT3-ITD
AML#8	Male	77	Newly diagnosed	M4	53.3	DNMT3A, NRAS
AML#9	Female	24	Newly diagnosed	M5	14.5	NA
AML#10	Female	69	Newly diagnosed	M5	101.2	CEBPA
AML#11	Male	62	Newly diagnosed	M5	16.0	FLT3-ITD, c-KIT
AML#12	Female	21	Newly diagnosed	M1	44.67	IDH1/IDH2
AML#13	Male	60	Newly diagnosed	M2	9.60	NA
AML#14	Female	70	Newly diagnosed	M5	66.0	NRAS, c-KIT
AML#15	Male	82	Newly diagnosed	M5	79.5	NPM1, IDH1/IDH2
AML#16	Male	37	Newly diagnosed	M4	4.71	CEBPA
AML#17	Female	49	Newly diagnosed	M4	22.03	FLT3-ITD
AML#18	Female	78	Newly diagnosed	M1	2.83	NA
AML#19	Male	49	Newly diagnosed	M4	36.99	NPM1,CEBPA
AML#20	Male	67	Newly diagnosed	M2	13.4	WT1, CEBPA

NA: not available
