# Supplementary figures and images for: Cytoplasmic TP53INP2 acts as an apoptosis partner in TRAIL treatment: the synergistic effect of TRAIL with venetoclax in TP53INP2-positive acute myeloid leukemia
Source: J Exp Clin Cancer Res. 2024 Jun 22;43:176. doi: 10.1186/s13046-024-03100-0 (PMC11193246; doi:10.1186/s13046-024-03100-0)

Additional file 2: Figure S1

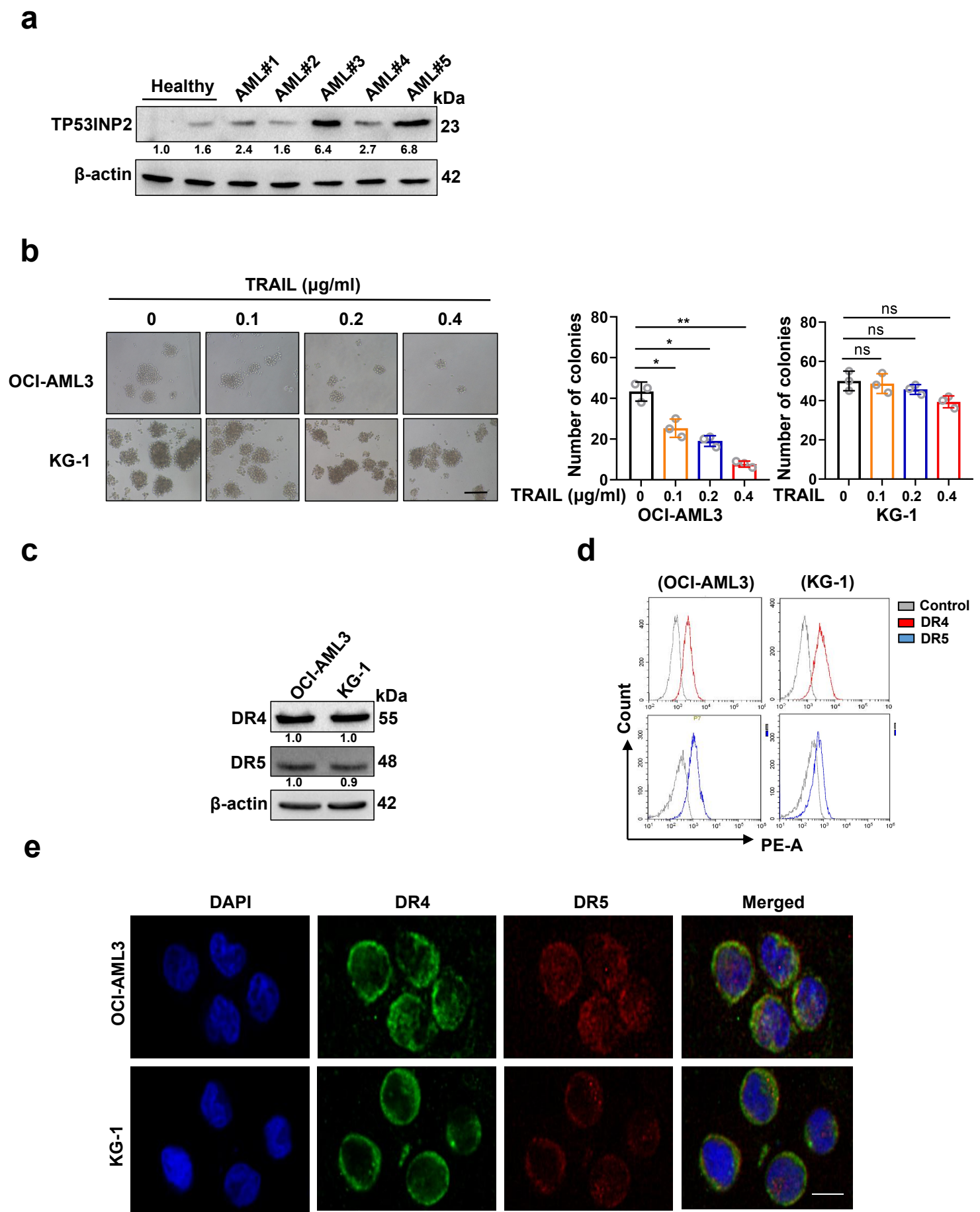

Supplement: Supplementary file 2 — Additional file 2: Figure S1. The expression of TP53INP2 is positively correlated with the sensitivity of AML cells to TRAIL treatment. a Western blot analysis of TP53INP2 levels in primary AML blasts and healthy donors. β-actin was used as a loading control, and the quantification of protein levels was shown below the protein bands. b Colony formation assay was performed in the OCI-AML3 (b, left and upper) and KG-1 cells (b, left and lower) treated with 0-0.4 μg/ml TRAIL for 48 h (Scale bar: 50 μm). The representative images and quantitative data from three independent experiments were shown in (b, middle) and (b, right), respectively. The cells incubated in a drug-free medium served as controls. c-e Western blot (c), FCM (d), and IF (e) analyses of DR4 and DR5 levels in the OCI-AML3 and KG-1 cells. The data are representative of at least three independent experiments. * p<0.05, ** p<0.01, ns, not significant. [file 13046_2024_3100_MOESM2_ESM.pdf]

Additional file 3: Figure S2

a

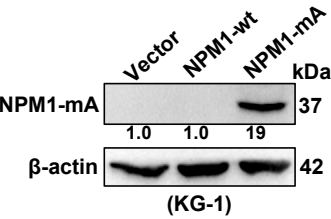

b

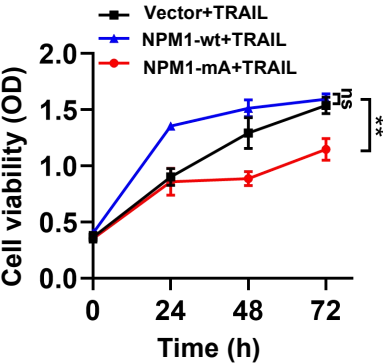

c

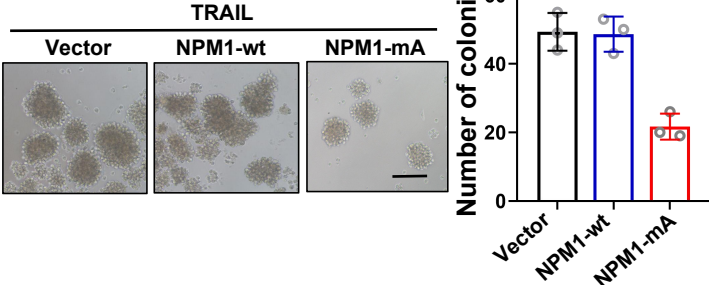

d

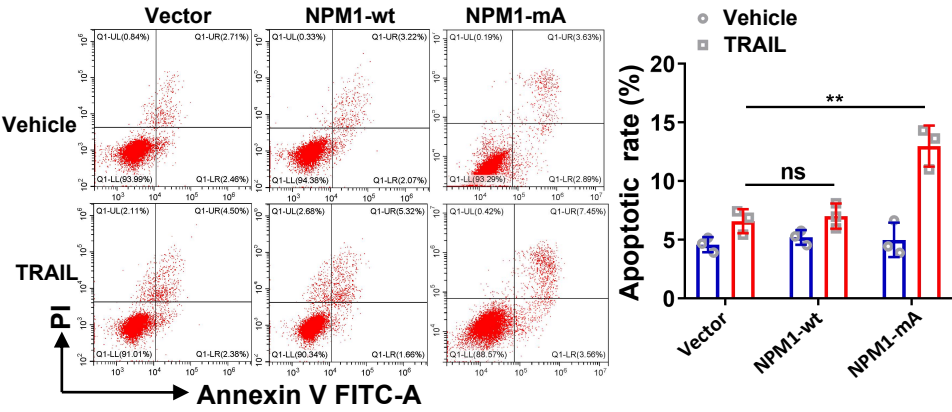

e

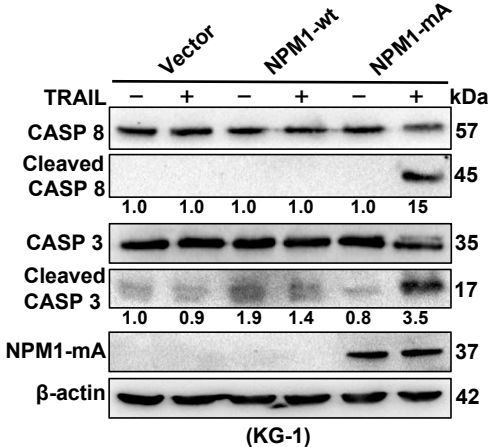

Supplement: Supplementary file 3 — Additional file 3: Figure S2. NPM1-mA expression, but not the NPM1-wt, rendered AML cells more sensitive to TRAIL. a Western blot analysis of NPM1-mA in KG-1 cells transfected with the NPM1-wt and NPM1-mA plasmids. b CCK-8 analysis (n =3) of cell viability in the cells treated with 100 ng/ml TRAIL for 0-72 h. c Colony formation assay was performed in KG-1 cells (Scale bar: 50 μm). The representative images and quantitative data from three independent experiments were shown in (c, left) and (c, right), respectively. d FCM analysis (d, left) and quantification (d, right) of apoptotic cells in the cells. The cells transfected with vector plasmid served as controls. e Western blot analysis of the indicated apoptosis-related proteins. In (a, e), β-actin was used as a loading control, and the quantification of protein levels was shown below the protein bands. The data are representative of at least three independent experiments. * p<0.05, ** p<0.01, ns, not significant. [file 13046_2024_3100_MOESM3_ESM.pdf]

Additional file 4: Figure S3

a

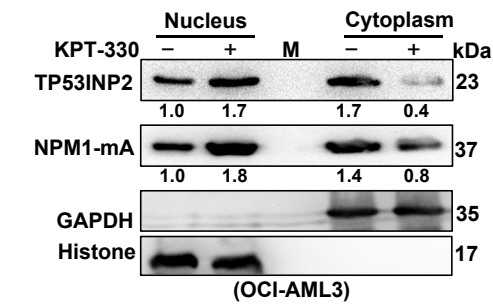

b

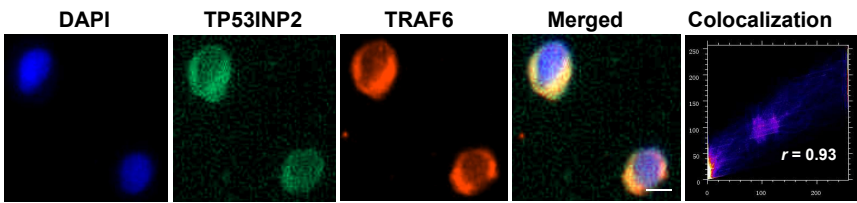

c

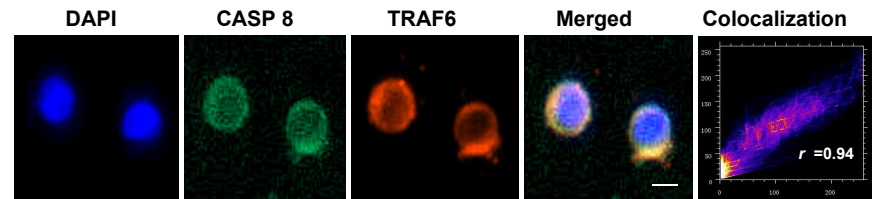

d

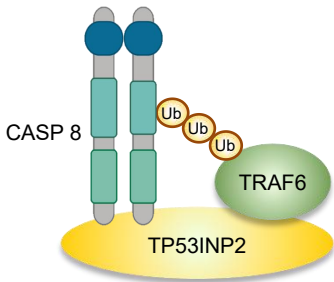

Supplement: Supplementary file 4 — Additional file 4: Figure S3. Cytoplasmic TP53INP2 functions as a scaffold linking TRAF6 with CASP 8. a Western blot analysis of TP53INP2 and NPM1-mA levels in the cytoplasm and nucleus of OCI-AML3 cells treated with 2 µM KPT-330 for 10 h. GAPDH was used as a loading control in the cytoplasm and Histone was used as a loading control in the nucleus. b IF analysis of endogenous TP53INP2 (green) and TRAF6 (red) co-localization in OCI-AML3 cells (Scale bar: 5 µm). c IF analysis of endogenous CASP 8 (green) and TRAF6 (red) co-localization in OCI-AML3 cells (Scale bar: 5 µm). Quantification of fluorescence co-localization was performed using Image J software, and r of 0.5~1.0 means that the two proteins are co-located in (b-c). d The schematic depiction of TP53INP2 linking TRAF6 and CASP 8 to promote the ubiquitination activation of CASP 8. [file 13046_2024_3100_MOESM4_ESM.pdf]

Additional file 5: Figure S4

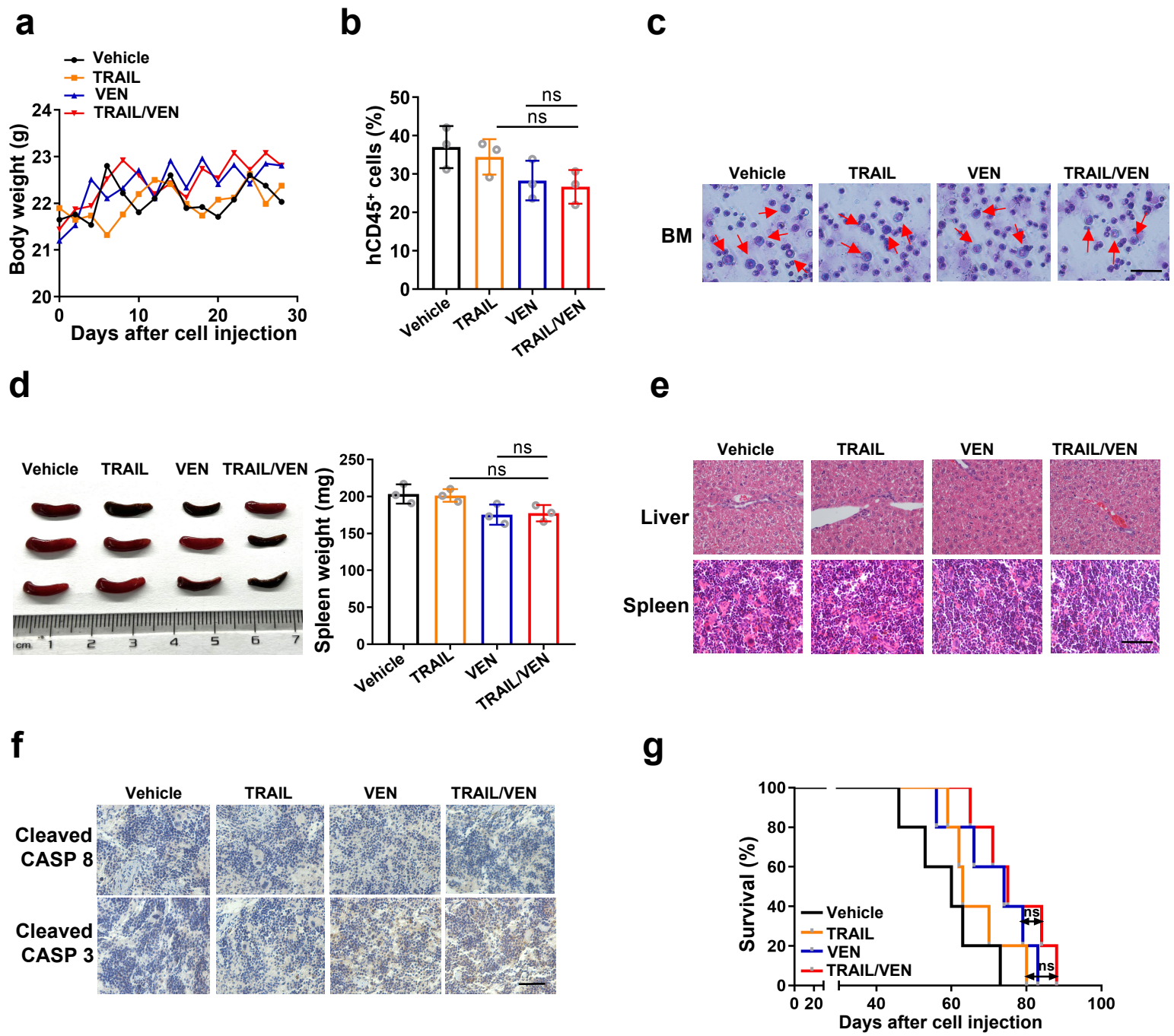

Supplement: Supplementary file 5 — Additional file 5: Figure S4. The effect of TRAIL/VEN combination on PDX generated with NMP1 wt AML. a The weights of all the mice were recorded during the experiment. b Quantification of hCD45+ leukemic cells from PDX mice by FCM (n=3). c Wright’s staining of bone marrow immature cells (Scale bar: 100 μm). The red arrow indicates the immature cells. d Images of spleen appearance. e H&E staining of liver (e, upper) and spleen (e, lower) from one representative mouse in each group (Scale bar: 50μm). f IHC staining of cleaved CASP 8 (f, upper) and cleaved CASP 3 (f, lower) expression in the spleen. g Kaplan-Meier survival curve of mice in each group. The mice treated with an equal amount of vehicle were used as controls. The data are representative of at least three independent experiments. BM, bone marrow. ns, not significant. [file 13046_2024_3100_MOESM5_ESM.pdf]
