# Supplementary material for: Cytoplasmic TP53INP2 acts as an apoptosis partner in TRAIL treatment: the synergistic effect of TRAIL with venetoclax in TP53INP2-positive acute myeloid leukemia
Source: J Exp Clin Cancer Res. 2024 Jun 22;43:176. doi: 10.1186/s13046-024-03100-0 (PMC11193246; doi:10.1186/s13046-024-03100-0)
Supplement: Supplementary file 6 — Additional file 6: The STR profile reports for AML cell lines. [file 13046_2024_3100_MOESM6_ESM.pdf]

Short Tandem Repeat（STR）Report

1. Sample info.

Sample Label：**OCI-AML3**

2. Data Interpretation

1) Human Cell Line Authentication Standardization of Short Tandem Repeat (STR) Profiling ASN-0002 Revised version 2021
2) The Pharmacopoeia of the People's Republic of China, Volume III General Requirements, Requirements for Preparation and Control of Animal Cell Substrates Used for Production of Biologics

3. Methodology

Sample DNA was extracted with genome extraction kit (Axygen) and PCR amplified with STR Multi-amplification Kit (MicroreaderTM21 ID System). PCR products were assayed with ABI 3730xl DNA Analyzer (Applied Biosystems®). Appropriate positive and negative controls were run and confirmed for each sample submitted. Data of 20 STR loci plus the Amelogenin locus were analyzed and extracted using Gene Mapper ID-X software and designated as the STR profile of the test sample.

4. Identify Result

The results of the negative and positive control match expectations.

The STR profiles of the test sample are clear, which are shown in the attached table and figure.

Test results for Multiple Alleles[3]: Number of multiple alleles: 0, Locus: None.

5. Explanation of Test Results

1) The submitted sample profile is human.
2) The submitted profile has a 100% match on 9 loci of the following human cell line(s) in DSMZ database：OCI-AML3.

Note: This report is only responsible for the samples submitted for this inspection.
Conclusion: According to ATCC ASN-0002-2021，Cell lines with ≥80% match at 13 STR loci or ≥90% match at eight STR loci are considered to be related.

Results of database comparison (the highest percent match to the query):

**Genotyping results of STR and Amelogenin loci in cells**						
Loci	STR information of submitted cells			STR information of cell bank		
	Submitted cells			Cell bank: OCI-AML3		
	Allele1	Allele2	Allele3	Allele1	Allele2	Allele3
D19S433	15	17				
D5S818	11	13		11	13	
D21S11	29	30				
D18S51	12	18				
D6S1043	11	13				
AMEL	X	X		X	X	
D3S1358	16	16				
D13S317	8	13		8	13	
D7S820	9	10		9	10	
D16S539	11	13		11	13	
CSF1PO	11	12		11	12	
PentaD	12	13				
D2S441	14	14				
vWA	17	18		17	18	
D8S1179	10	12				
TPOX	8	11		8	11	
PentaE	11	13				
TH01	8	10		8	10	
D12S391	20	21				
D2S1338	17	21				
FGA	22	22				

Remarks

1. The short tandem repeat (STR) profile is indicative only of the sample sent to Suzhou Haixing Co., Ltd at the time it was sent. Effective peaks are real PCR bands, small peaks and non-specific bands are ignored in calculation.
2. The STR genotyping profile of the submitted cell is compared with ATCC, DSMZ and ExPASy databases, in which STR genotypes of over 100,000 human cell lines from ATCC, DSMZ, JCRB, ECACC, Riken etc. were recorded. Cells that are not included in above databases could not be compared. For failed sample, it's genotyping and figure result would be shown as NA.
3. Both mixed cultures and MSI positive cell lines can show additional STR alleles in one or more STR loci. In short, two fundamentally different causes can be the reason for multiple alleles at a locus: drifted alleles by MSI or some other DNA replication error or the presence of a mixed culture containing a contaminating second human cell line.
4. The number of STR loci shared by the submitted sample and the cells in database. Amelogenin does not belong to STR locus.
5. The matching score of DSMZ and ExPASyis "Tanabe"— 2 x (number of alleles matching) / （total number of alleles in database profile and query sample） x 100%. The matching score of ATCC is (the number of shared alleles between query sample and database profile) / （total number of alleles in database profile） x 100%.
6. The interpretation is based on "ATCC ASN-0002-2021"."Two samples are considered possibly related when their STR profiles match at 80-90% of alleles across the 13 core STR loci, as calculated using the matching algorithm. At matches of 90% or greater, the cell lines have very likely originated from the same donor. Samples between 60 and 79% match tend to be unrelated, but need may further investigation to confirm that they are unrelated. Two common causes of related cell lines scoring below 80% are (1) if the cell line has extra alleles due to it being MSI unstable, and (2) misidentification of the cell line's origin. Generally, cell lines are authentic (i.e., are derived from the same original tissue donor) when they show the expected percent match (⩾ 80% at 13 STR loci or > 90% match at eight STR loci) to other results from that donor (tissue or other cell line samples within the database)."
7. The testing results were calculated and generated by each cell database ONLINE tool. In some case, there may be different profiles of same cell line in each database and possibly lead to different matching results. In addition, limited by the algorithm of the calculation model (the less the number of shared STR sites, the higher the probability of matching unrelated cells), non-cell line(s)human cells/sample(s) may also match to cell(s) in database. In these conditions, the comparison results in this report are for reference only.
8. The results are judged according to industry standards or consensus and can only be used for cell line inspection and authentication. We are NOT responsible for any purpose or interpretation other than the purpose specified.

Figure 1: Genotyping of STR and Amelogenin loci in cells

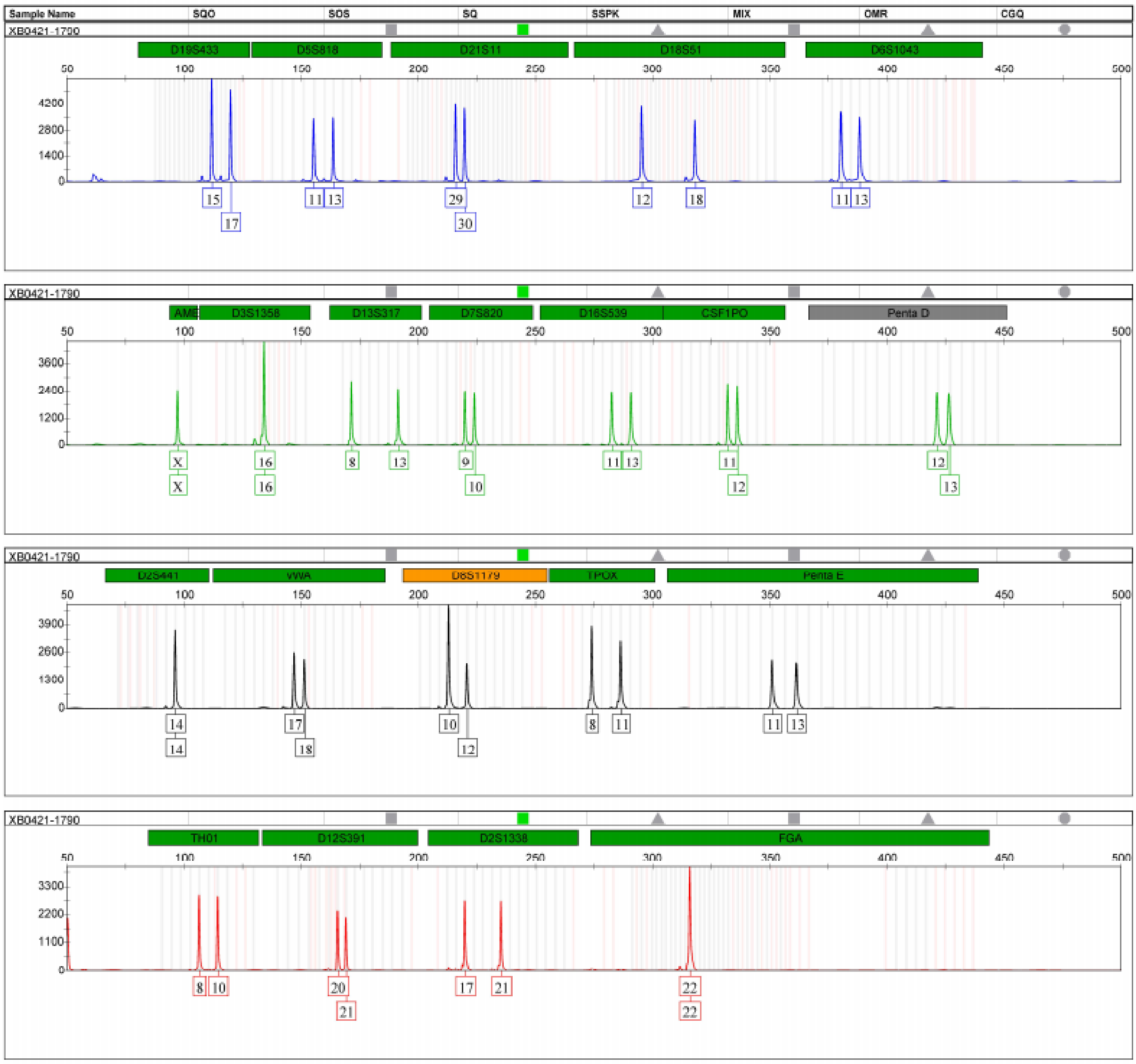

Short Tandem Repeat（STR） Report

1. Sample info.

Sample Label：**KG-1**

2. Data Interpretation

1) Human Cell Line Authentication Standardization of Short Tandem Repeat (STR) Profiling ASN-0002 Revised version 2021
2) The Pharmacopoeia of the People's Republic of China, Volume III General Requirements, Requirements for Preparation and Control of Animal Cell Substrates Used for Production of Biologics

3. Methodology

Sample DNA was extracted with genome extraction kit (Axygen) and PCR amplified with STR Multi-amplification Kit (MicroreaderTM21 ID System). PCR products were assayed with ABI 3730xl DNA Analyzer (Applied Biosystems®). Appropriate positive and negative controls were run and confirmed for each sample submitted. Data of 20 STR loci plus the Amelogenin locus were analyzed and extracted using Gene Mapper ID-X software and designated as the STR profile of the test sample.

4. Identify Result

The results of the negative and positive control match expectations.

The STR profiles of the test sample are clear, which are shown in the attached table and figure.

Test results for Multiple Alleles[3]: Number of multiple alleles: 0, Locus: None.

5. Explanation of Test Results

1) The submitted sample profile is human.
2) The submitted profile has a 100% match on 9 loci of the following human cell line(s) in DSMZ database：KG-1.

Note: This report is only responsible for the samples submitted for this inspection.

Conclusion: According to ATCC ASN-0002-2021，Cell lines with ≥80% match at 13 STR loci or ≥90% match at eight STR loci are considered to be related.

Results of database comparison (the highest percent match to the query)：

Genotyping results of STR and Amelogenin loci in cells						
Loci	STR information of submitted cells			STR information of cell bank		
	Submitted cells			Cell bank：KG-1		
	Allele1	Allele2	Allele3	Allele1	Allele2	Allele3
D5S818	13	13		13	13	
D13S317	11	12		11	12	
D7S820	8	10		8	10	
D16S539	10	11		10	11	
VWA	14	19		14	19	
TH01	7	8		7	8	
AMEL	X	Y		X	Y	
TPOX	7	9		7	9	
CSF1PO	7	7		7	7	
D12S391	20	21				
FGA	22	22				
D2S1338	20	23				
D21S11	28	29				
D18S51	10.2	18				
D8S1179	13	14				
D3S1358	15	16				
D6S1043	18	19				
PENTAE	7	13				
D19S433	11	16.2				
PENTAD	8	9				
D1S1656	14	16.3				

Remarks

1. The short tandem repeat (STR) profile is indicative only of the sample sent to Suzhou Haixing Co., Ltd at the time it was sent. Effective peaks are real PCR bands, small peaks and non-specific bands are ignored in calculation.

2. The STR genotyping profile of the submitted cell is compared with ATCC, DSMZ and ExPASy databases, in which STR genotypes of over 100,000 human cell lines from ATCC, DSMZ, JCRB, ECACC, Riken etc. were recorded. Cells that are not included in above databases could not be compared. For failed sample, it's genotyping and figure result would be shown as NA.

3. Both mixed cultures and MSI positive cell lines can show additional STR alleles in one or more STR loci. In short, two fundamentally different causes can be the reason for multiple alleles at a locus: drifted alleles by MSI or some other DNA replication error or the presence of a mixed culture containing a contaminating second human cell line.

4. The number of STR loci shared by the submitted sample and the cells in database. Amelogenin does not belong to STR locus.

5. The matching score of DSMZ and ExPASyis "Tanabe"— 2 x (number of alleles matching) / (total number of alleles in database profile and query sample) x 100%. The matching score of ATCC is (the number of shared alleles between query sample and database profile) / (total number of alleles in database profile) x 100%.

6. The interpretation is based on "ATCC ASN-0002-2021"."Two samples are considered possibly related when their STR profiles match at 80-90% of alleles across the 13 core STR loci, as calculated using the matching algorithm. At matches of 90% or greater, the cell lines have very likely originated from the same donor. Samples between 60 and 79% match tend to be unrelated, but need may further investigation to confirm that they are unrelated. Two common causes of related cell lines scoring below 80% are (1) if the cell line has extra alleles due to it being MSI unstable, and (2) misidentification of the cell line's origin. Generally, cell lines are authentic (i.e., are derived from the same original tissue donor) when they show the expected percent match (⩾ 80% at 13 STR loci or > 90% match at eight STR loci) to other results from that donor (tissue or other cell line samples within the database)."

7. The testing results were calculated and generated by each cell database ONLINE tool. In some case, there may be different profiles of same cell line in each database and possibly lead to different matching results. In addition, limited by the algorithm of the calculation model (the less the number of shared STR sites, the higher the probability of matching unrelated cells), non-cell line(s)human cells/sample(s) may also match to cell(s) in database. In these conditions, the comparison results in this report are for reference only.

8. The results are judged according to industry standards or consensus and can only be used for cell line inspection and authentication. We are NOT responsible for any purpose or interpretation other than the purpose specified.

Figure 1: Genotyping of STR and Amelogenin loci in cells

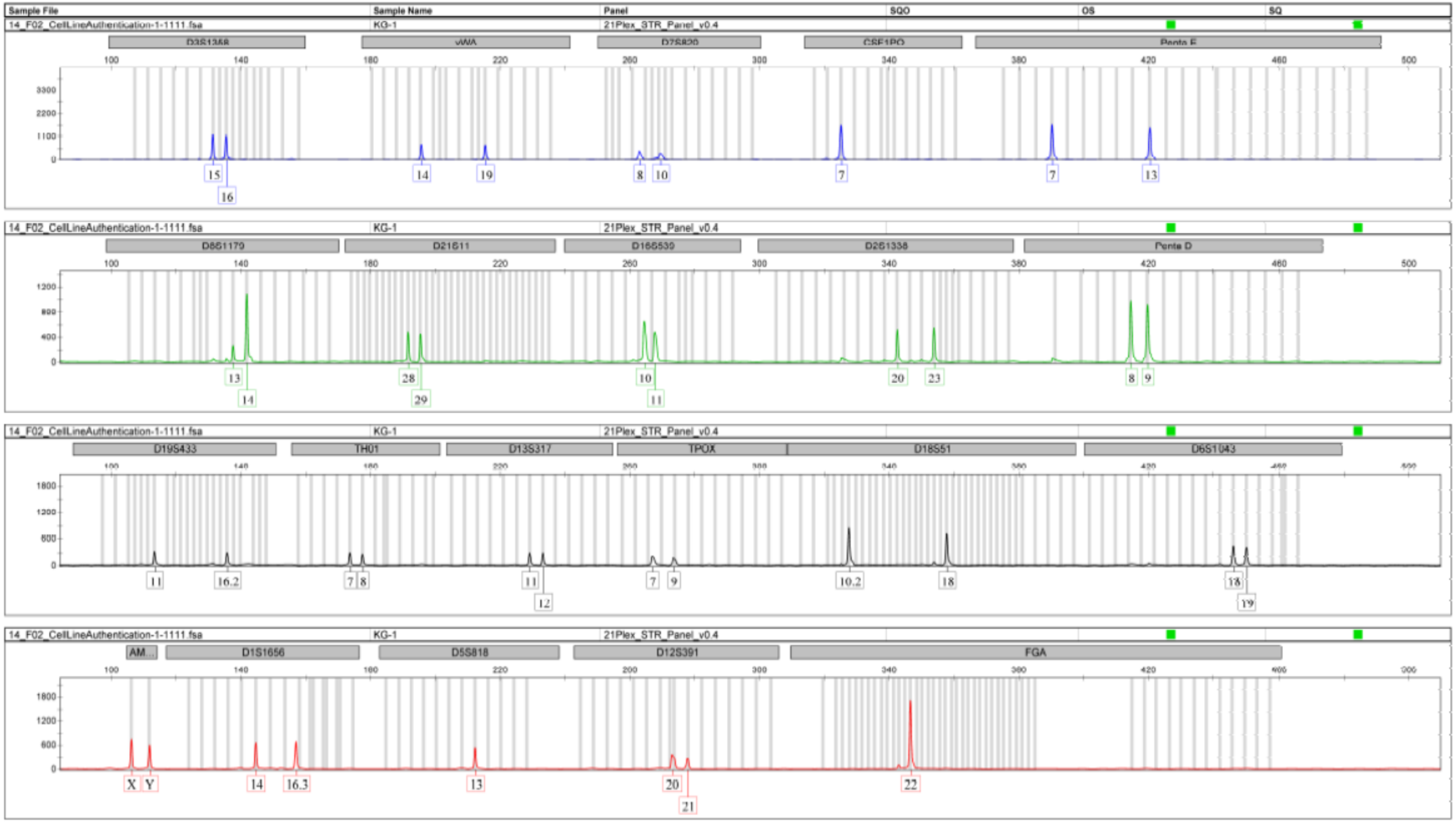

Short Tandem Repeat（STR） Report

1. Sample info.

Sample Label：**HL-60**

2. Data Interpretation

1) Human Cell Line Authentication Standardization of Short Tandem Repeat (STR) Profiling ASN-0002 Revised version 2021
2) The Pharmacopoeia of the People's Republic of China, Volume III General Requirements, Requirements for Preparation and Control of Animal Cell Substrates Used for Production of Biologics

3. Methodology

Sample DNA was extracted with genome extraction kit (Axygen) and PCR amplified with STR Multi-amplification Kit (MicroreaderTM21 ID System). PCR products were assayed with ABI 3730xl DNA Analyzer (Applied Biosystems®). Appropriate positive and negative controls were run and confirmed for each sample submitted. Data of 20 STR loci plus the Amelogenin locus were analyzed and extracted using Gene Mapper ID-X software and designated as the STR profile of the test sample.

4. Identify Result

The results of the negative and positive control match expectations.

The STR profiles of the test sample are clear, which are shown in the attached table and figure.

Test results for Multiple Alleles[3]: Number of multiple alleles: 0, Locus: None.

5. Explanation of Test Results

1) The submitted sample profile is human.
2) The submitted profile has a 100% match on 9 loci of the following human cell line(s) in DSMZ database：HL60.

Note: This report is only responsible for the samples submitted for this inspection.
Conclusion: According to ATCC ASN-0002-2021，Cell lines with ≥80% match at 13 STR loci or ≥90% match at eight STR loci are considered to be related.

Results of database comparison (the highest percent match to the query)：

Genotyping results of STR and Amelogenin loci in cells						
Loci	STR information of submitted cells			STR information of cell bank		
	Submitted cells			Cell bank：HL-60		
	Allele1	Allele2	Allele3	Allele1	Allele2	Allele3
D5S818	12	12		12	12	
D13S317	8	11		8	11	
D7S820	11	12		11	12	
D16S539	11	11		11	11	
VWA	16	16		16	16	
TH01	7	8		7	8	
AMEL	X	X		X	X	
TPOX	8	11		8	11	
CSF1PO	13	14		13	14	
D12S391	18	20				
FGA	22	24				
D2S1338	17	17				
D21S11	29	30				
D18S51	14	15				
D8S1179	12	13				
D3S1358	16	16				
D6S1043	11	12				
PENTAE	13	14				
D19S433	14	14				
PENTAD	10	12				
D1S1656	15	15				

Remarks

1. The short tandem repeat (STR) profile is indicative only of the sample sent to Suzhou Haixing Co., Ltd at the time it was sent. Effective peaks are real PCR bands, small peaks and non-specific bands are ignored in calculation.

2. The STR genotyping profile of the submitted cell is compared with ATCC, DSMZ and ExPASy databases, in which STR genotypes of over 100,000 human cell lines from ATCC, DSMZ, JCRB, ECACC, Riken etc. were recorded. Cells that are not included in above databases could not be compared. For failed sample, it's genotyping and figure result would be shown as NA.

3. Both mixed cultures and MSI positive cell lines can show additional STR alleles in one or more STR loci. In short, two fundamentally different causes can be the reason for multiple alleles at a locus: drifted alleles by MSI or some other DNA replication error or the presence of a mixed culture containing a contaminating second human cell line.

4. The number of STR loci shared by the submitted sample and the cells in database. Amelogenin does not belong to STR locus.

5. The matching score of DSMZ and ExPASyis "Tanabe"— 2 x (number of alleles matching) / (total number of alleles in database profile and query sample) x 100%. The matching score of ATCC is (the number of shared alleles between query sample and database profile) / (total number of alleles in database profile) x 100%.

6. The interpretation is based on "ATCC ASN-0002-2021"."Two samples are considered possibly related when their STR profiles match at 80-90% of alleles across the 13 core STR loci, as calculated using the matching algorithm. At matches of 90% or greater, the cell lines have very likely originated from the same donor. Samples between 60 and 79% match tend to be unrelated, but need may further investigation to confirm that they are unrelated. Two common causes of related cell lines scoring below 80% are (1) if the cell line has extra alleles due to it being MSI unstable, and (2) misidentification of the cell line's origin. Generally, cell lines are authentic (i.e., are derived from the same original tissue donor) when they show the expected percent match (⩾ 80% at 13 STR loci or > 90% match at eight STR loci) to other results from that donor (tissue or other cell line samples within the database)."

7. The testing results were calculated and generated by each cell database ONLINE tool. In some case, there may be different profiles of same cell line in each database and possibly lead to different matching results. In addition, limited by the algorithm of the calculation model (the less the number of shared STR sites, the higher the probability of matching unrelated cells), non-cell line(s)human cells/sample(s) may also match to cell(s) in database. In these conditions, the comparison results in this report are for reference only.

8. The results are judged according to industry standards or consensus and can only be used for cell line inspection and authentication. We are NOT responsible for any purpose or interpretation other than the purpose specified.

Figure 1: Genotyping of STR and Amelogenin loci in cells

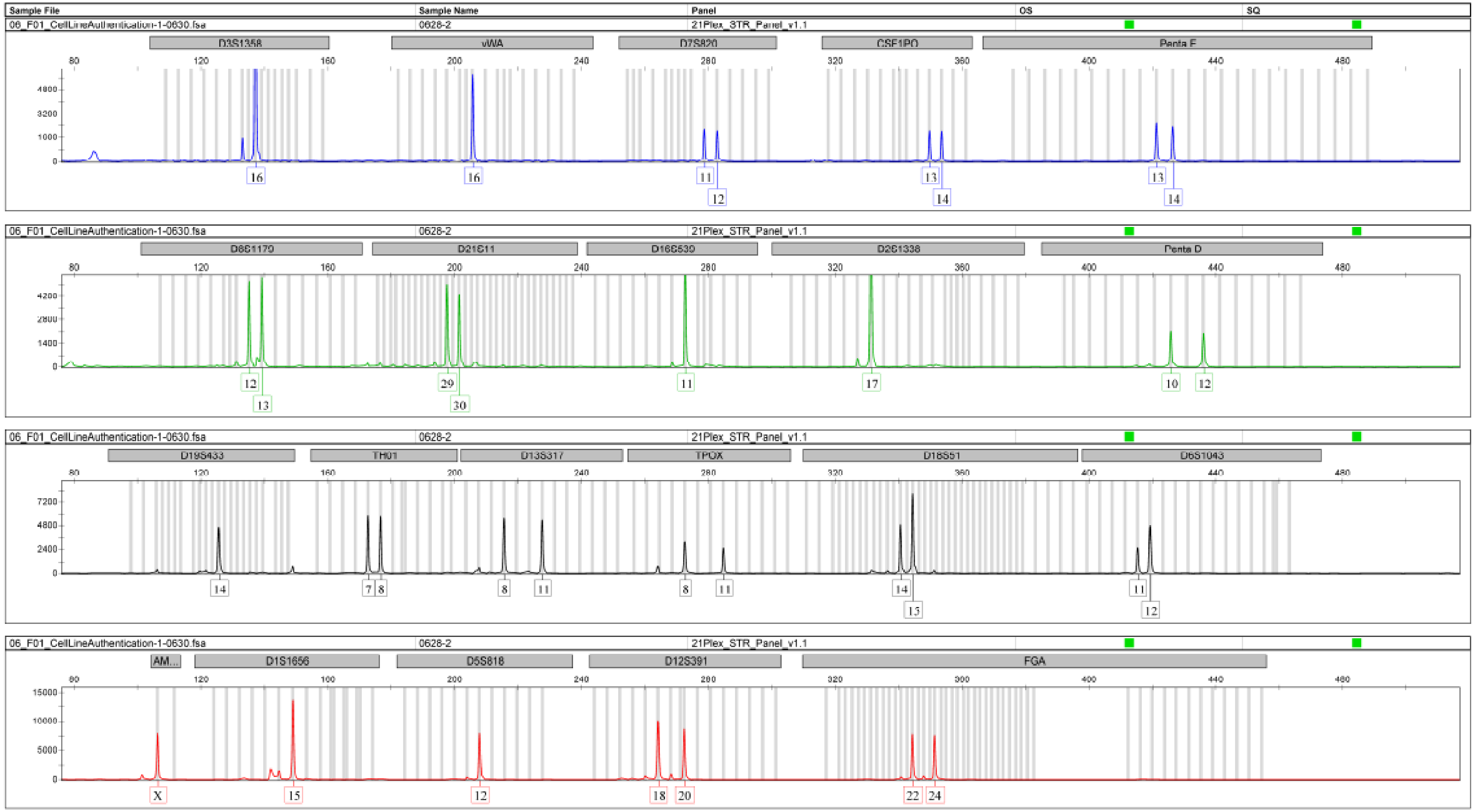

Short Tandem Repeat（STR） Report

1. Sample info.

Sample Label：**NB4**

2. Data Interpretation

1) Human Cell Line Authentication Standardization of Short Tandem Repeat (STR) Profiling ASN-0002 Revised version 2021
2) The Pharmacopoeia of the People's Republic of China, Volume III General Requirements, Requirements for Preparation and Control of Animal Cell Substrates Used for Production of Biologics

3. Methodology

Sample DNA was extracted with genome extraction kit (Axygen) and PCR amplified with STR Multi-amplification Kit (MicroreaderTM21 ID System). PCR products were assayed with ABI 3730xl DNA Analyzer (Applied Biosystems®). Appropriate positive and negative controls were run and confirmed for each sample submitted. Data of 20 STR loci plus the Amelogenin locus were analyzed and extracted using Gene Mapper ID-X software and designated as the STR profile of the test sample.

4. Identify Result

The results of the negative and positive control match expectations.

The STR profiles of the test sample are clear, which are shown in the attached table and figure.

Test results for Multiple Alleles[3]: Number of multiple alleles: 0, Locus: None.

5. Explanation of Test Results

1) The submitted sample profile is human.
2) The submitted profile has a 100% match on 9 loci of the following human cell line(s) in DSMZ database：NB4.

Note: This report is only responsible for the samples submitted for this inspection.
Conclusion: According to ATCC ASN-0002-2021，Cell lines with ≥80% match at 13 STR loci or ≥90% match at eight STR loci are considered to be related.

Results of database comparison (the highest percent match to the query)：

Genotyping results of STR and Amelogenin loci in cells						
Loci	STR information of submitted cells			STR information of cell bank		
	Submitted cells			Cell bank： NB4		
	Allele1	Allele2	Allele3	Allele1	Allele2	Allele3
D5S818	13	13		13	13	
D13S317	11	12		11	12	
D7S820	10	13		10	13	
D16S539	9	9		9	9	
VWA	16	19		16	19	
TH01	7	9.3		7	9.3	
AMEL	X	X		X	X	
TPOX	8	11		8	11	
CSF1PO	11	12		11	12	
D12S391	19	22				
FGA	21	22				
D2S1338	20	25				
D21S11	28	33.2				
D18S51	12	12				
D8S1179	10	10				
D3S1358	15	17				
D6S1043	12	12				
PENTAE	7	13				
D19S433	15	15				
PENTAD	10	13				
D1S1656	12	15				

Remarks

1. The short tandem repeat (STR) profile is indicative only of the sample sent to Suzhou Haixing Co., Ltd at the time it was sent. Effective peaks are real PCR bands, small peaks and non-specific bands are ignored in calculation.
2. The STR genotyping profile of the submitted cell is compared with ATCC, DSMZ and ExPASy databases, in which STR genotypes of over 100,000 human cell lines from ATCC, DSMZ, JCRB, ECACC, Riken etc. were recorded. Cells that are not included in above databases could not be compared. For failed sample, it's genotyping and figure result would be shown as NA.
3. Both mixed cultures and MSI positive cell lines can show additional STR alleles in one or more STR loci. In short, two fundamentally different causes can be the reason for multiple alleles at a locus: drifted alleles by MSI or some other DNA replication error or the presence of a mixed culture containing a contaminating second human cell line.
4. The number of STR loci shared by the submitted sample and the cells in database. Amelogenin does not belong to STR locus.
5. The matching score of DSMZ and ExPASyis "Tanabe"— 2 x (number of alleles matching) / （total number of alleles in database profile and query sample） x 100%. The matching score of ATCC is (the number of shared alleles between query sample and database profile) / （total number of alleles in database profile） x 100%.
6. The interpretation is based on "ATCC ASN-0002-2021"."Two samples are considered possibly related when their STR profiles match at 80-90% of alleles across the 13 core STR loci, as calculated using the matching algorithm. At matches of 90% or greater, the cell lines have very likely originated from the same donor. Samples between 60 and 79% match tend to be unrelated, but need may further investigation to confirm that they are unrelated. Two common causes of related cell lines scoring below 80% are (1) if the cell line has extra alleles due to it being MSI unstable, and (2) misidentification of the cell line's origin. Generally, cell lines are authentic (i.e., are derived from the same original tissue donor) when they show the expected percent match (⩾ 80% at 13 STR loci or > 90% match at eight STR loci) to other results from that donor (tissue or other cell line samples within the database)."
7. The testing results were calculated and generated by each cell database ONLINE tool. In some case, there may be different profiles of same cell line in each database and possibly lead to different matching results. In addition, limited by the algorithm of the calculation model (the less the number of shared STR sites, the higher the probability of matching unrelated cells), non-cell line(s)human cells/sample(s) may also match to cell(s) in database. In these conditions, the comparison results in this report are for reference only.
8. The results are judged according to industry standards or consensus and can only be used for cell line inspection and authentication. We are NOT responsible for any purpose or interpretation other than the purpose specified.

Figure 1: Genotyping of STR and Amelogenin loci in cells

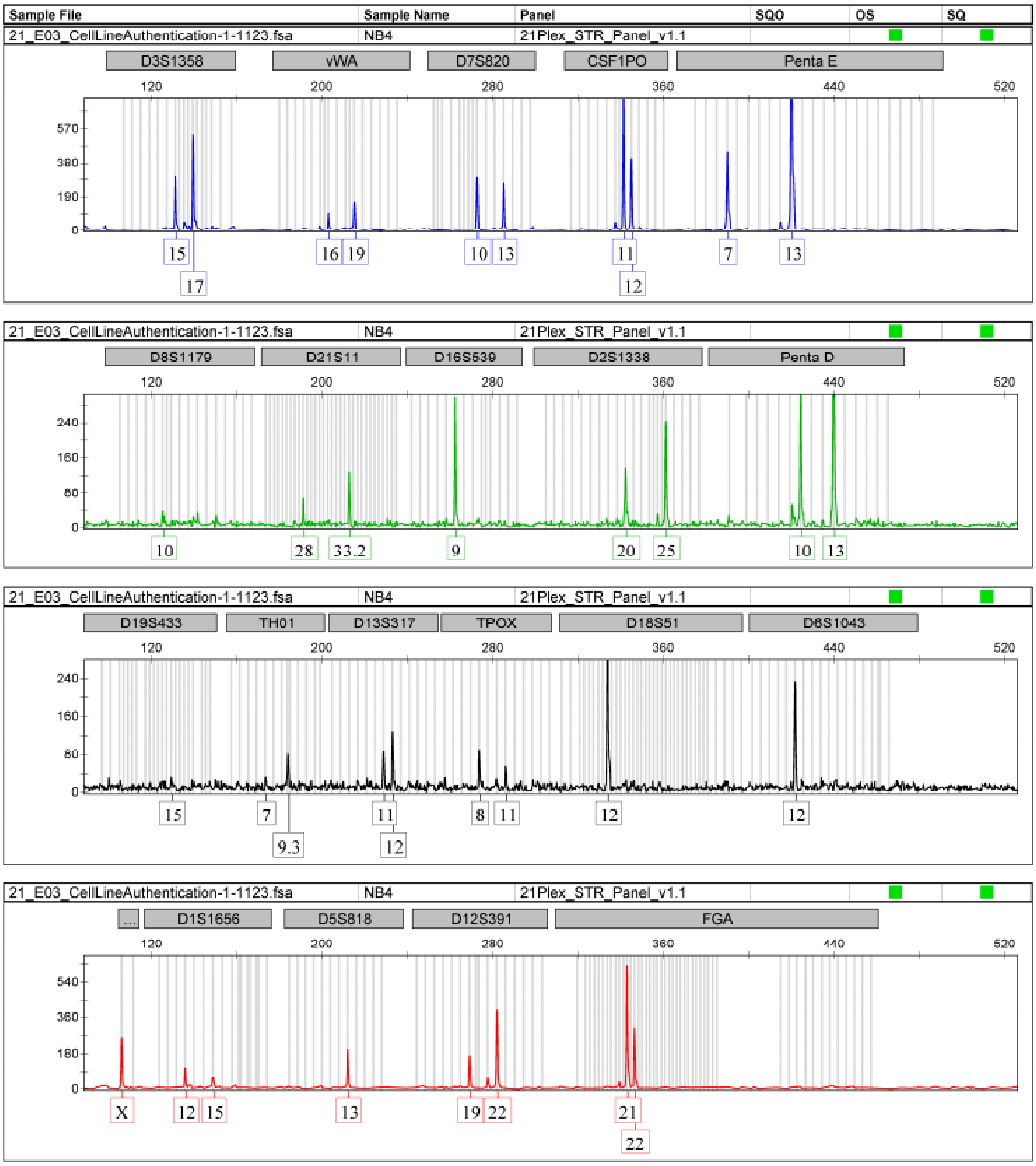

.

Short Tandem Repeat（STR） Report

1. Sample info.

Sample Label：**THP-1**

2. Data Interpretation

1) Human Cell Line Authentication Standardization of Short Tandem Repeat (STR) Profiling ASN-0002 Revised version 2021
2) The Pharmacopoeia of the People's Republic of China, Volume III General Requirements, Requirements for Preparation and Control of Animal Cell Substrates Used for Production of Biologics

3. Methodology

Sample DNA was extracted with genome extraction kit (Axygen) and PCR amplified with STR Multi-amplification Kit (MicroreaderTM21 ID System). PCR products were assayed with ABI 3730xl DNA Analyzer (Applied Biosystems®). Appropriate positive and negative controls were run and confirmed for each sample submitted. Data of 20 STR loci plus the Amelogenin locus were analyzed and extracted using Gene Mapper ID-X software and designated as the STR profile of the test sample.

4. Identify Result

The results of the negative and positive control match expectations.

The STR profiles of the test sample are clear, which are shown in the attached table and figure.

Test results for Multiple Alleles[3]: Number of multiple alleles: 0, Locus: None.

5. Explanation of Test Results

1) The submitted sample profile is human.
2) The submitted profile has a 97% match on 9 loci of the following human cell line(s) in DSMZ database：THP-1.

Note: This report is only responsible for the samples submitted for this inspection.
Conclusion: According to ATCC ASN-0002-2021，Cell lines with ≥80% match at 13 STR loci or ≥90% match at eight STR loci are considered to be related.

Results of database comparison (the highest percent match to the query)：

Genotyping results of STR and Amelogenin loci in cells						
Loci	STR information of submitted cells			STR information of cell bank		
	Submitted cells			Cell bank：THP-1		
	Allele1	Allele2	Allele3	Allele1	Allele2	Allele3
D5S818	11	12		11	12	
D13S317	13	13		13	13	
D7S820	10	10		10	10	
D16S539	11	12		11	12	
VWA	16	16		16	16	
TH01	8	9.3		8	9.3	
AMEL	X	Y		X	Y	
TPOX	8	11		8	11	
CSF1PO	11	13		11	13	
D12S391	19	19				
FGA	24	25				
D2S1338	17	18				
D21S11	30	31.2				
D18S51	13	14				
D8S1179	10	14				
D3S1358	15	17				
D6S1043	14	14				
PENTAE	11	15				
D19S433	12.2	13				
PENTAD	10	12				
D1S1656	16	17				

Remarks

1. The short tandem repeat (STR) profile is indicative only of the sample sent to Suzhou Haixing Co., Ltd at the time it was sent. Effective peaks are real PCR bands, small peaks and non-specific bands are ignored in calculation.
2. The STR genotyping profile of the submitted cell is compared with ATCC, DSMZ and ExPASy databases, in which STR genotypes of over 100,000 human cell lines from ATCC, DSMZ, JCRB, ECACC, Riken etc. were recorded. Cells that are not included in above databases could not be compared. For failed sample, it's genotyping and figure result would be shown as NA.
3. Both mixed cultures and MSI positive cell lines can show additional STR alleles in one or more STR loci. In short, two fundamentally different causes can be the reason for multiple alleles at a locus: drifted alleles by MSI or some other DNA replication error or the presence of a mixed culture containing a contaminating second human cell line.
4. The number of STR loci shared by the submitted sample and the cells in database. Amelogenin does not belong to STR locus.
5. The matching score of DSMZ and ExPASyis "Tanabe"— 2 x (number of alleles matching) / (total number of alleles in database profile and query sample） x 100%. The matching score of ATCC is (the number of shared alleles between query sample and database profile) / （total number of alleles in database profile） x 100%.
6. The interpretation is based on "ATCC ASN-0002-2021"."Two samples are considered possibly related when their STR profiles match at 80-90% of alleles across the 13 core STR loci, as calculated using the matching algorithm. At matches of 90% or greater, the cell lines have very likely originated from the same donor. Samples between 60 and 79% match tend to be unrelated, but need may further investigation to confirm that they are unrelated. Two common causes of related cell lines scoring below 80% are (1) if the cell line has extra alleles due to it being MSI unstable, and (2) misidentification of the cell line's origin. Generally, cell lines are authentic (i.e., are derived from the same original tissue donor) when they show the expected percent match (⩾ 80% at 13 STR loci or > 90% match at eight STR loci) to other results from that donor (tissue or other cell line samples within the database)."
7. The testing results were calculated and generated by each cell database ONLINE tool. In some case, there may be different profiles of same cell line in each database and possibly lead to different matching results. In addition, limited by the algorithm of the calculation model (the less the number of shared STR sites, the higher the probability of matching unrelated cells), non-cell line(s)human cells/sample(s) may also match to cell(s) in database. In these conditions, the comparison results in this report are for reference only.
8. The results are judged according to industry standards or consensus and can only be used for cell line inspection and authentication. We are NOT responsible for any purpose or interpretation other than the purpose specified.

Figure 1: Genotyping of STR and Amelogenin loci in cells

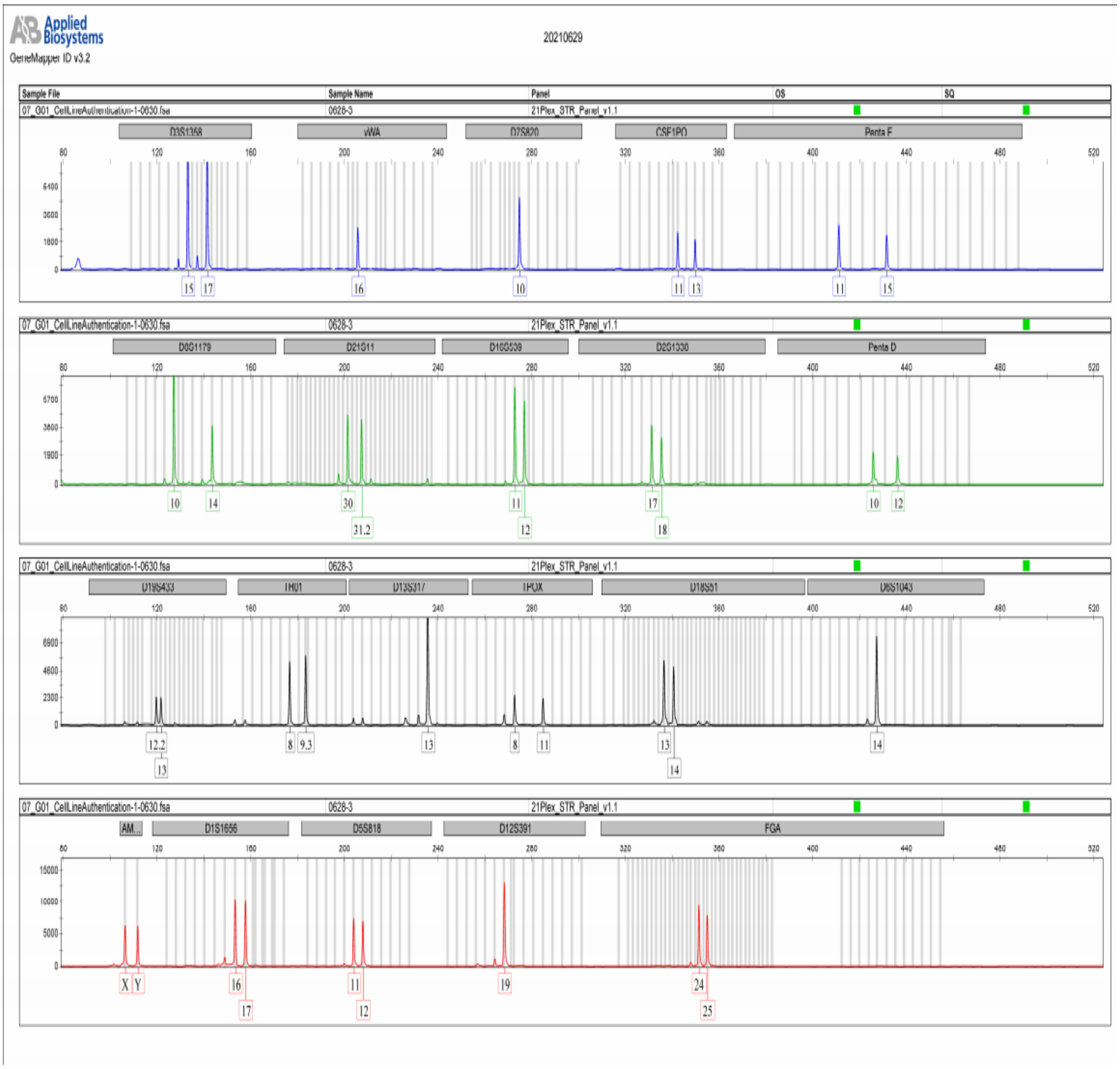

Short Tandem Repeat（STR）Report

1. Sample info.

Sample Label：**U937**

2. Data Interpretation

1) Human Cell Line Authentication Standardization of Short Tandem Repeat (STR) Profiling ASN-0002 Revised version 2021
2) The Pharmacopoeia of the People's Republic of China, Volume III General Requirements, Requirements for Preparation and Control of Animal Cell Substrates Used for Production of Biologics

3. Methodology

Sample DNA was extracted with genome extraction kit (Axygen) and PCR amplified with STR Multi-amplification Kit (MicroreaderTM21 ID System). PCR products were assayed with ABI 3730xl DNA Analyzer (Applied Biosystems®). Appropriate positive and negative controls were run and confirmed for each sample submitted. Data of 20 STR loci plus the Amelogenin locus were analyzed and extracted using Gene Mapper ID-X software and designated as the STR profile of the test sample.

4. Identify Result

The results of the negative and positive control match expectations.

The STR profiles of the test sample are clear, which are shown in the attached table and figure.

Test results for Multiple Alleles[3]: Number of multiple alleles: 0, Locus: None.

5. Explanation of Test Results

1) The submitted sample profile is human.
2) The submitted profile has a 100% match on 9 loci of the following human cell line(s) in DSMZ database：U937.

Note: This report is only responsible for the samples submitted for this inspection.
Conclusion: According to ATCC ASN-0002-2021，Cell lines with ≥80% match at 13 STR loci or ≥90% match at eight STR loci are considered to be related.

Results of database comparison (the highest percent match to the query)：

Genotyping results of STR and Amelogenin loci in cells						
Loci	STR information of submitted cells			STR information of cell bank		
	Submitted cells			Cell bank：U937		
	Allele1	Allele2	Allele3	Allele1	Allele2	Allele3
D19S433	14	16				
D5S818	12	12		12	12	
D21S11	27	29				
D18S51	13	14				
D6S1043	12	18				
AMEL	X	X		X	X	
D3S1358	16	16				
D13S317	10	12		10	12	
D7S820	9	11		9	11	
D16S539	12	12		12	12	
CSF1PO	12	12		12	12	
PentaD	12	13				
D2S441	10	10				
vWA	14	15		14	15	
D8S1179	12	13				
TPOX	8	11		8	11	
PentaE	13	13				
TH01	6	9.3		6	9.3	
D12S391	17	18				
D2S1338	17	20				
FGA	22	25				

Remarks

1. The short tandem repeat (STR) profile is indicative only of the sample sent to Suzhou Haixing Co., Ltd at the time it was sent. Effective peaks are real PCR bands, small peaks and non-specific bands are ignored in calculation.
2. The STR genotyping profile of the submitted cell is compared with ATCC, DSMZ and ExPASy databases, in which STR genotypes of over 100,000 human cell lines from ATCC, DSMZ, JCRB, ECACC, Riken etc. were recorded. Cells that are not included in above databases could not be compared. For failed sample, it's genotyping and figure result would be shown as NA.
3. Both mixed cultures and MSI positive cell lines can show additional STR alleles in one or more STR loci. In short, two fundamentally different causes can be the reason for multiple alleles at a locus: drifted alleles by MSI or some other DNA replication error or the presence of a mixed culture containing a contaminating second human cell line.
4. The number of STR loci shared by the submitted sample and the cells in database. Amelogenin does not belong to STR locus.
5. The matching score of DSMZ and ExPASyis "Tanabe"— 2 x (number of alleles matching) / (total number of alleles in database profile and query sample) x 100%. The matching score of ATCC is (the number of shared alleles between query sample and database profile) / (total number of alleles in database profile) x 100%.
6. The interpretation is based on "ATCC ASN-0002-2021"."Two samples are considered possibly related when their STR profiles match at 80-90% of alleles across the 13 core STR loci, as calculated using the matching algorithm. At matches of 90% or greater, the cell lines have very likely originated from the same donor. Samples between 60 and 79% match tend to be unrelated, but need may further investigation to confirm that they are unrelated. Two common causes of related cell lines scoring below 80% are (1) if the cell line has extra alleles due to it being MSI unstable, and (2) misidentification of the cell line's origin. Generally, cell lines are authentic (i.e., are derived from the same original tissue donor) when they show the expected percent match (⩾ 80% at 13 STR loci or > 90% match at eight STR loci) to other results from that donor (tissue or other cell line samples within the database)."
7. The testing results were calculated and generated by each cell database ONLINE tool. In some case, there may be different profiles of same cell line in each database and possibly lead to different matching results. In addition, limited by the algorithm of the calculation model (the less the number of shared STR sites, the higher the probability of matching unrelated cells), non-cell line(s)human cells/sample(s) may also match to cell(s) in database. In these conditions, the comparison results in this report are for reference only.
8. The results are judged according to industry standards or consensus and can only be used for cell line inspection and authentication. We are NOT responsible for any purpose or interpretation other than the purpose specified.

Figure 1: Genotyping of STR and Amelogenin loci in cells

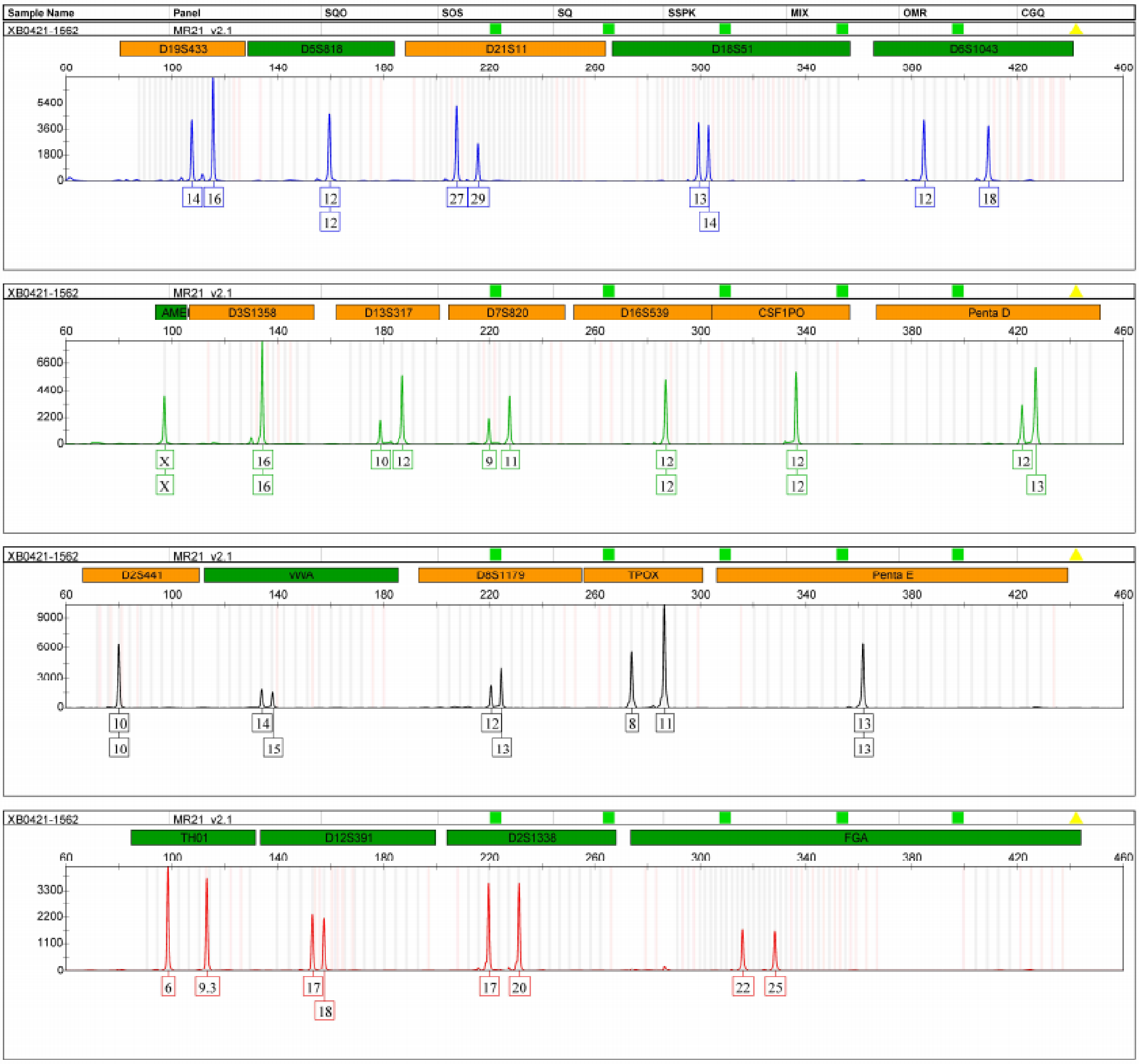

.

Cell Line Authentication Service
STR Profile Report

GENETIC TESTING BIOTECHNOLOGY

**Sample Submitted By:**	Dr. Zuokang Zheng<br>Zhejiang Meisen Cell Technology Co.,Ltd.
**Email Address:**	947638289@qq.com
**Sales Order:**	210422C
**Cell Line Designation:**	OCI-AML-2
**Date Sample Received:**	Apr 22th, 2021
**Report Date:**	Apr 23th, 2021

Methodology: Nineteen short tandem repeat (STR) loci plus the gender determining locus, Amelogenin, were amplified using the commercially available EX20 Kit from AGCU. The cell line sample was processed using the ABI Prism® 3130 XL Genetic Analyzer. Data were analyzed using GeneMapper® ID v3.2 software (Applied Biosystems). Appropriate positive and negative controls were run and confirmed for each sample submitted.

Data Interpretation: Cell lines were authenticated using Short Tandem Repeat (STR) analysis as described in 2012 in ANSI Standard (ASN-0002) by the ATCC Standards Development Organization (SDO) and in Capes-Davis et al., Match criteria for human cell line authentication: Where do we draw the line? Int J Cancer. 2013;132(11):2510-9.

GTB™ performs STR Profiling following ISO 9001:2008 and ISO/IEC 17025:2005 quality standards.
There are no warranties with respect to the services or results supplied, express or implied, including, without limitation, any implied warranty of merchantability or fitness for a particular purpose. Genetic Testing Biotechnology (GTB) is not liable for any damages or injuries resulting from receipt and/or improper, inappropriate, negligent or other wrongful use of the test results supplied, and/or from misidentification, misrepresentation, or lack of accuracy of those results. Your exclusive remedy against GTB and those supplying materials used in the services for any losses or damage of any kind whatsoever, whether in contract, tort, or otherwise, shall be, at GTB's option, refund of the fee paid for such service or repeat of the service.

NOTE: According to the recommendations of *IJC* on cell line authentication, the report is valid for 3 years since the issue date.

Technical Questions?
GTB Technical Support
+86-512-67486171
service@jsdna.org
Section 505, Yixin BLD
SIP, Suzhou, 215123
Jiangsu, P.R. China

Ordering Questions?
order@jsdna.org
GTB Corporation
+86-512-62806339
Section 303, Yixin BLD
SIP, Suzhou, 215123
Jiangsu, P.R. China

GENETIC TESTING BIOTECHNOLOGY

Cell Line Authentication Service
STR Profile Report

Sales Order: 210422C

	Test Results for Submitted Sample		DSMZ Reference Database Profile	
Loci	Query Profile: OCI-AML-2		Database Profile: OCI-AML-2	
Amelogenin	X	Y	X	Y
D3S1358	16			
D13S317	11	12	11	12
D7S820	12		12	
D16S539	12	13	12	13
Penta E	12	14		
TPOX	11		11	
TH01	6	9.3	6	9.3
D2S1338	23	25		
CSF1PO	10	12	10	12
Penta D	12			
D19S433	13	14		
vWA	14	17	14	17
D21S11	29			
D18S51	13	14		
D6S1043	17	19		
D8S1179	12	15		
D5S818	12		12	
D12S391	25	26		
FGA	19	24		

The allele match algorithm compares the 8 core loci plus amelogenin only, even though alleles from all loci will be reported when available.

Note: Loci highlighted in grey (8 core STR loci plus Amelogenin) can be made public to verify cell identity. In order to protect the identity of the donor, ***please do not publish*** *the allele calls from all the STR loci tested. The sample match is based on the reference data available at the time of comparison.*

Explanation of Test Results

Cell lines with ≥80% match are considered to be related; i.e., derived from a common ancestry. Cell lines with between a 55% to 80% match require further profiling for authentication of relatedness.

☐ The submitted sample profile is human, but not a match for any profile in the DSMZ STR database.

☑ The submitted profile is an exact match for the following human cell line(s) in the DSMZ STR database (8 core loci plus Amelogenin): OCI-AML-2

☐ The submitted profile is similar to the following DSMZ human cell line(s):

e-Signature, Technician:
Digitally signed by Xuekun Chen
DN: cn=Xuekun Chen, o=Genetic Testing Biotechnology (Suzhou), ou=DNA Typing Section, email=order@jsdna.org, c=CN
Date: 2021.04.23 11:03:22 +08'00'

GENETIC TESTING BIOTECHNOLOGY GUARANTEE CELL LINE AUTHENTICATION ★ ★ ★ ★ ★

e-Signature, Reviewer:
Digitally signed by Xiankun Zhao
DN: cn=Xiankun Zhao, o=Genetic Testing Biotechnology (Suzhou), ou=Supervision Section, email=service@jsdna.org, c=CN
Date: 2021.04.23 11:06:20 +08'00'

Addendum: Electropherogram for the customer's sample set 1 of 1

GENETIC TESTING BIOTECHNOLOGY

Cell Line Authentication Service
STR Profile Report

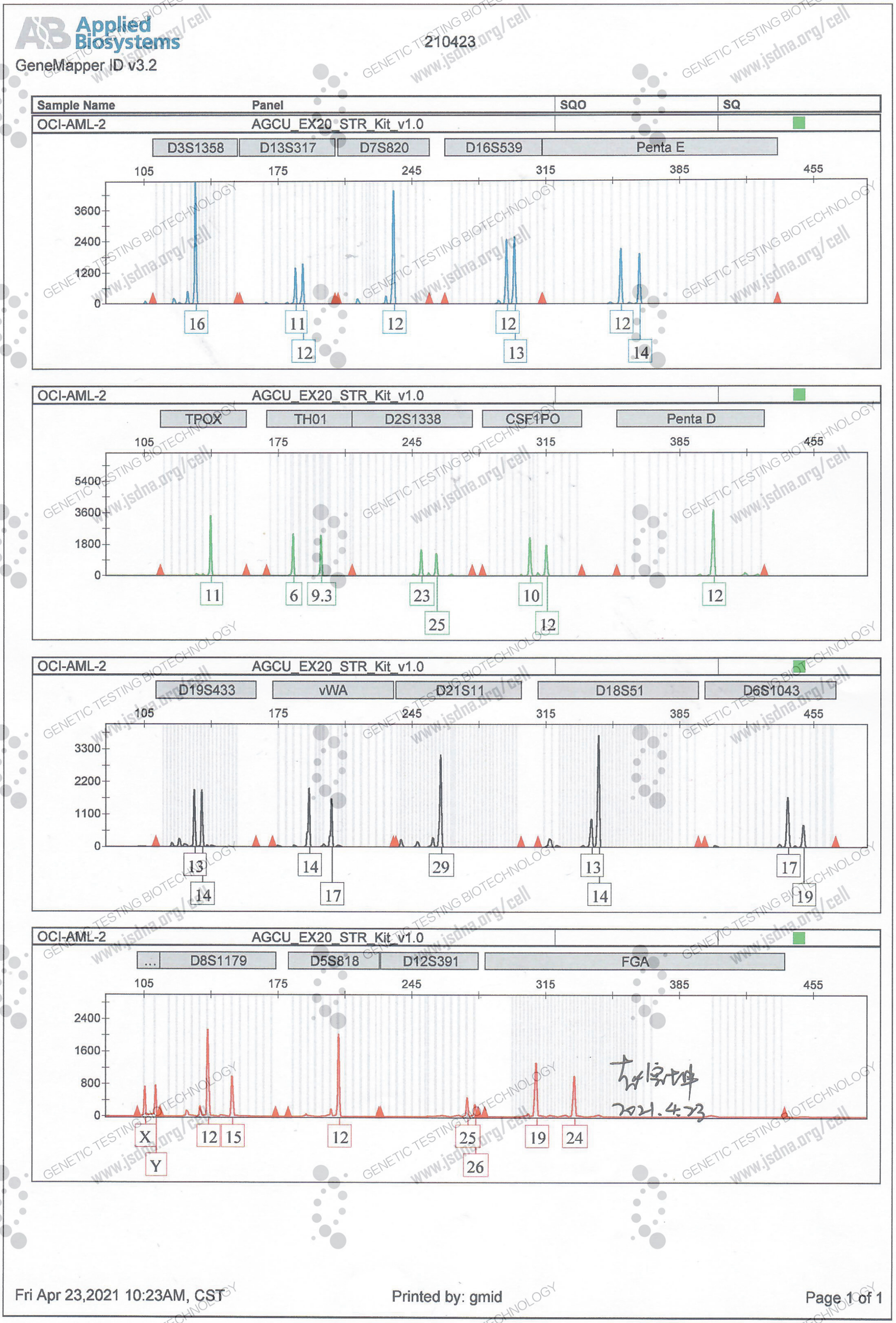

GENETIC TESTING BIOTECHNOLOGY www.jsdna.org/cell

Result of STR matching analysis by your data.

- DSMZ Profile Database -

A graphical presentation is shown at the bottom of this page.

EV	Cell No.	Cell name	Locus names									Figures
			D5S818	D13S317	D7S820	D16S539	VWA	TH01	AM	TPOX	CSF1PO	
	Query (Your Cell)		12,12	11,12	12,12	12,13	14,17	6,9.3	X,Y	11,11	10,12	
1.00(36/36)	99	OCI-AML-2	12,12	11,12	12,12	12,13	14,17	6,9.3	X,Y	11,11	10,12	-
0.78(28/36)	CRL-5829	NCI-H290	11,12	11,12	10,12	9,12	14,17	6,9.3	X,Y	11,11	10,10	-
0.72(26/36)	CRL-1473	HT-1197	12,12	11,12	11,12	12,13	16,18	6,9.3	X,Y	11,12	11,12	-
0.72(26/36)	CRL-7681	Hs 929.Sk	12,12	9,12	12,12	12,13	16,17	7,9.3	X,Y	8,11	10,11	-
0.72(26/36)	RCB1881	TL-Mor	11,12	11,12	11,12	12,13	14,17	6,9	X,Y	9,11	7,12	-
0.67(24/36)	234	A-427	12,12	11,12	8,12	11,13	17,17	9,9	X,Y	8,11	10,12	-
0.67(24/36)	303	SK-MEL-1	12,13	11,11	12,12	11,12	16,17	6,6	X,Y	11,11	12,13	-
0.67(24/36)	CRL-1498	CCD-39Lu	11,12	11,11	9,12	11,13	14,17	6,9.3	X,X	8,11	10,12	-
0.67(24/36)	CRL-1501	CCD-39Sk	11,12	11,11	9,12	11,13	14,17	6,9.3	X,X	8,11	10,12	-
0.67(24/36)	CRL-1610	GS-109-V-20	11,12	12,12	9,12	11,12	14,17	7,9.3	X,Y	8,11	10,12	-
0.67(24/36)	CRL-2097	CCD-1079Sk	11,12	11,12	12,12	9,11	17,18	6,9.3	X,Y	10,11	12,13	-
0.67(24/36)	CRL-2272	CHP-234	11,12	11,12	8,10	12,13	15,17	6,9.3	X,X	11,11	12,12	-
0.67(24/36)	CRL-2503	NL20	12,12	11,12	12,12	12,12	14,17	6,7	X,X	9,11	11,11	-
0.67(24/36)	CRL-2504	NL20-TA	12,12	11,12	12,12	12,12	14,17	6,7	X,X	9,11	11,11	-
0.67(24/36)	CRL-7023	Hs 39.T	12,13	11,12	8,9	12,13	15,19	6,9.3	X,Y	11,11	11,12	-
0.67(24/36)	CRL-7671	Hs 919.Sk	12,12	11,12	10,10	11,13	16,17	6,9.3	X,X	8,11	10,12	-
0.67(24/36)	CRL-7672	Hs 919.T	12,12	11,12	10,10	11,13	16,17	6,9.3	X,X	8,11	10,12	-
0.67(24/36)	CRL-7906	A-427	12,12	11,12	8,12	11,13	17,17	9,9	X,Y	8,11	10,12	-
0.67(24/36)	CRL-9856	KMA	12,13	11,12	11,12	12,13	17,17	6,9	X,Y	8,9	10,12	-
0.67(24/36)	HTB-53	A-427	12,12	11,12	8,12	11,13	17,17	9,9	X,Y	8,11	10,12	-
0.67(24/36)	HTB-67	SK-MEL-1	12,13	11,11	12,12	11,12	16,17	6,6	X,Y	11,11	12,13	-
0.67(24/36)	JCRB9079	Ran Nor	11,12	8,12	11,12	12,13	15,17	6,7	X,Y	8,11	10,12	-
0.67(24/36)	JCRB9112	D422T	11,12	12,12	9,12	11,12	14,17	7,9.3	X,Y	8,11	10,12	-

GENETIC TESTING BIOTECHNOLOGY

Cell Line Authentication Service
STR Profile Report

Sample Submitted By: Dr. Zuokang Zheng
Zhejiang Meisen Cell Technology Co.,Ltd.
Email Address: 947638289@qq.com
Sales Order: 210422D
Cell Line Designation: OCI-AML-5
Date Sample Received: Apr 22th, 2021
Report Date: Apr 23th, 2021

Methodology: Nineteen short tandem repeat (STR) loci plus the gender determining locus, Amelogenin, were amplified using the commercially available EX20 Kit from AGCU. The cell line sample was processed using the ABI Prism® 3130 XL Genetic Analyzer. Data were analyzed using GeneMapper® ID v3.2 software (Applied Biosystems). Appropriate positive and negative controls were run and confirmed for each sample submitted.

Data Interpretation: Cell lines were authenticated using Short Tandem Repeat (STR) analysis as described in 2012 in ANSI Standard (ASN-0002) by the ATCC Standards Development Organization (SDO) and in Capes-Davis et al., Match criteria for human cell line authentication: Where do we draw the line? Int J Cancer. 2013;132(11):2510-9.

GTB™ performs STR Profiling following ISO 9001:2008 and ISO/IEC 17025:2005 quality standards.
There are no warranties with respect to the services or results supplied, express or implied, including, without limitation, any implied warranty of merchantability or fitness for a particular purpose. Genetic Testing Biotechnology (GTB) is not liable for any damages or injuries resulting from receipt and/or improper, inappropriate, negligent or other wrongful use of the test results supplied, and/or from misidentification, misrepresentation, or lack of accuracy of those results. Your exclusive remedy against GTB and those supplying materials used in the services for any losses or damage of any kind whatsoever, whether in contract, tort, or otherwise, shall be, at GTB's option, refund of the fee paid for such service or repeat of the service.

NOTE: According to the recommendations of *IJC* on cell line authentication, the report is valid for 3 years since the issue date.

Technical Questions?
GTB Technical Support
+86-512-67486171
service@jsdna.org
Section 505, Yixin BLD
SIP, Suzhou, 215123
Jiangsu, P.R. China

Ordering Questions?
order@jsdna.org
GTB Corporation
+86-512-62806339
Section 303, Yixin BLD
SIP, Suzhou, 215123
Jiangsu, P.R. China

GENETIC TESTING BIOTECHNOLOGY

Cell Line Authentication Service
STR Profile Report

Sales Order: 210422D

	Test Results for Submitted Sample		ExPASy Reference Database Profile	
Loci	Query Profile: OCI-AML-5		Database Profile: OCI-AML-5	
Amelogenin	X	Y	X	Y
D3S1358	18			
D13S317	11	14	11	14
D7S820	11	12	11	12
D16S539	11	13	11	13
Penta E	13	18		
TPOX	8	11	8	11
TH01	9.3		9.3	
D2S1338	19	22		
CSF1PO	10	11	10	11
Penta D	9			
D19S433	12	15.2		
vWA	16	18	16	18
D21S11	28	32.2		
D18S51	15	17		
D6S1043	11	12		
D8S1179	13	14		
D5S818	12	13	12	13
D12S391	15	22		
FGA	19	24		

The allele match algorithm compares the 8 core loci plus amelogenin only, even though alleles from all loci will be reported when available.

Note: Loci highlighted in grey (8 core STR loci plus Amelogenin) can be made public to verify cell identity. In order to protect the identity of the donor, ***please do not publish*** *the allele calls from all the STR loci tested. The sample match is based on the reference data available at the time of comparison.*

Explanation of Test Results

Cell lines with ≥80% match are considered to be related; i.e., derived from a common ancestry. Cell lines with between a 55% to 80% match require further profiling for authentication of relatedness.

- [ ] The submitted sample profile is human, but not a match for any profile in the ExPASy STR database.
- [x] The submitted profile is an exact match for the following human cell line(s) in the ExPASy STR database (8 core loci plus Amelogenin): OCI-AML-5
- [ ] The submitted profile is similar to the following ExPASy human cell line(s):

e-Signature, Technician: Digitally signed by Xuekun Chen
DN: cn=Xuekun Chen, o=Genetic Testing Biotechnology (Suzhou), ou=DNA Typing Section, email=order@jsdna.org, c=CN
Date: 2021.04.23 11:05:37 +08'00'

GENETIC TESTING BIOTECHNOLOGY — GUARANTEE — CELL LINE AUTHENTICATION

e-Signature, Reviewer: Digitally signed by Xiankun Zhao
DN: cn=Xiankun Zhao, o=Genetic Testing Biotechnology (Suzhou), ou=Supervision Section, email=service@jsdna.org, c=CN
Date: 2021.04.23 11:06:50 +08'00'

Addendum: Electropherogram for the customer's sample set 1 of 1

Cell Line Authentication Service
STR Profile Report

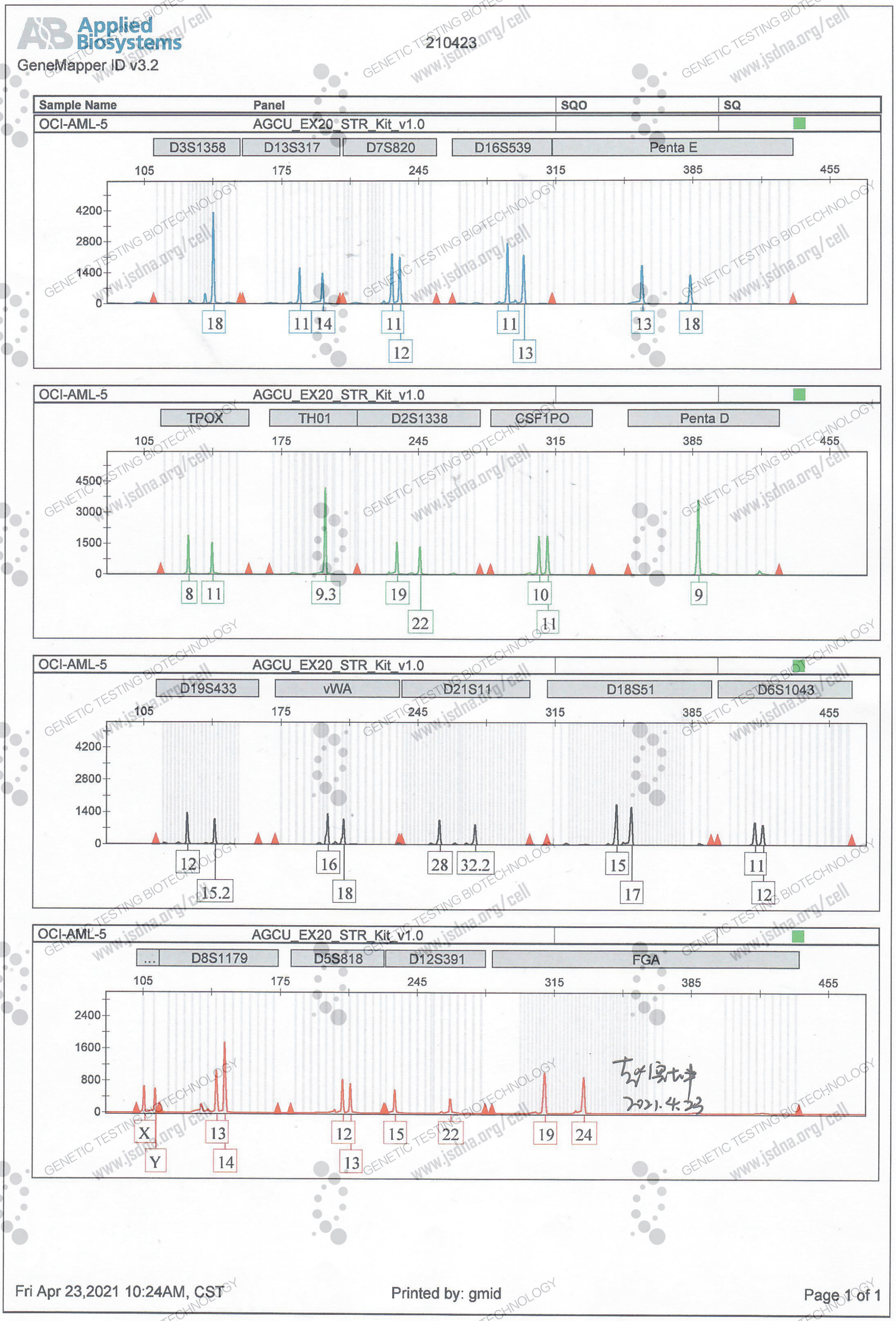

GENETIC TESTING BIOTECHNOLOGY

Cell Line Authentication Service
STR Profile Report

Accession	Name	Nº Markers	Score	Amel	CSF1PO	D2S1338	D3S1358	D5S818	D7S820	D8S1179	D13S317	D16S539	D18S51	D19S433	D21S11	FGA	Penta D	Penta E	TH01	TPOX	vWA
NA	Query	NA	NA	X,Y	10,11			12,13	11,12		11,14	11,13							9.3,9.3	8,11	16,18
CVCL_1620 *Best*	OCI-AML-5	9	100.00%	X,Y	10,11		18	12,13	11,12	13,14	11,14	11,13	15,17		28,32.2	19,24	9	13,18	9.3	8,11	16,18
CVCL_1620 *Worst*	OCI-AML-5	9	97.14%	X,Y	10,11		18	12,13	11,12	13,14	11,14	11,13	15,17		28,32.2	19,24	9	13,18	7,9.3	8,11	16,18
CVCL_N708	BT088	9	78.79%	X,Y	10,11			12,13	11,12		9	11,13							8,9	8,11	18
CVCL_RZ69	XYFMGG04D	9	78.79%	X	11,12			12,13	11,12		11	11,13							7,9.3	8,11	16,19
CVCL_6316	C5B7	9	77.42%	X,Y	9,11			12	12		11,12	11,13							9.3	8	16,18
CVCL_5134	HCC364	9	77.42%	X,Y	12	25	14,16	12,13	10,12	12,14	11	11	13	13	29	20,24	13	12,18	9.3	8,11	16,18
CVCL_3290	HIAE-65	9	77.42%	X	10,11			12	9,12		11,14	11,13							6	8	16,18
CVCL_3294	HIVE-65	9	77.42%	X	10,11			12	9,12		11,14	11,13							6	8	16,18
CVCL_0753	Hs 412.Lu	9	76.47%	X,Y	10,11			12,13	10,11		9,11	12							8,9.3	8,11	16,18
CVCL_A1VI	ICC4	9	76.47%	X,Y	10,11		15	11,13	11,12	13,14	10,11	11,12	12		27,31.2	20,21	10,11	10,11	9,9.3	8,11	18
CVCL_A9UQ	GSC#314C	9	75.00%	X	10			12,13	10,12		11,14	11							6,9.3	8,11	16,19
CVCL_A9UR	GSC#314P	9	75.00%	X	10			12,13	10,12		11,14	11							6,9.3	8,11	16,19
CVCL_RP21	MESO-3T	9	75.00%	X,Y	10,11		18	13	10,11	11,15	11	11,13	13,14		31,33.2	24	10	7,13	6,9.3	8,11	17
CVCL_1430	MSTO-211H	9	75.00%	X,Y	11,12		15	12	8,12	13	11,14	13	16,18		28,31	21	11,12	7,13	8,9.3	11	16,18
CVCL_4903	SUSM-1	9	75.00%	X,Y	10,11			12,13	10,11		11	12							8	8,11	16,18
CVCL_WP79	ESi070-A	9	74.29%	X,Y	11,12			12,13	9,11		11,12	9,11			27,28				8,9.3	8,11	16,18
CVCL_VV72	MUG-Mel1	9	73.33%	X,Y	10,11		17,18	12	8,11	13,14	11,12	11	13,14		31,31.2	21,24	9,10	5,12	9.3	8	16
CVCL_VV73	MUG-Mel1 clone C8	9	73.33%	X,Y	10,11		17,18	12	8,11	13,14	11,12	11	13,14		31,31.2	21,24	9,10	5,12	9.3	8	16
CVCL_VV74	MUG-Mel1 clone D5	9	73.33%	X,Y	10,11		17,18	12	8,11	13,14	11,12	11	13,14		31,31.2	21,24	9,10	5,12	9.3	8	16
CVCL_1668 *Best*	RPMI-8866	9	73.33%	X	11		15,16	12	11,12	12,14	10,14	11	12,15		27,29	22,23			9.3	8,11	14,16
CVCL_1668 *Worst*	RPMI-8866	9	66.67%	X	11		15,16	12	11,12	12,14	10,14	11	12,15		27,29	22,23			10	8,11	14,16
CVCL_1096 *Best*	C2BBe1	9	72.73%	X	11		14,17	12,13	11,12	12,14	11,13,14	12,13	12		30	19	9,11	7	6	9,11	16,18
