# Supplementary material for: Cytoplasmic TP53INP2 acts as an apoptosis partner in TRAIL treatment: the synergistic effect of TRAIL with venetoclax in TP53INP2-positive acute myeloid leukemia
Source: J Exp Clin Cancer Res. 2024 Jun 22;43:176. doi: 10.1186/s13046-024-03100-0 (PMC11193246; doi:10.1186/s13046-024-03100-0)

Fig. 1

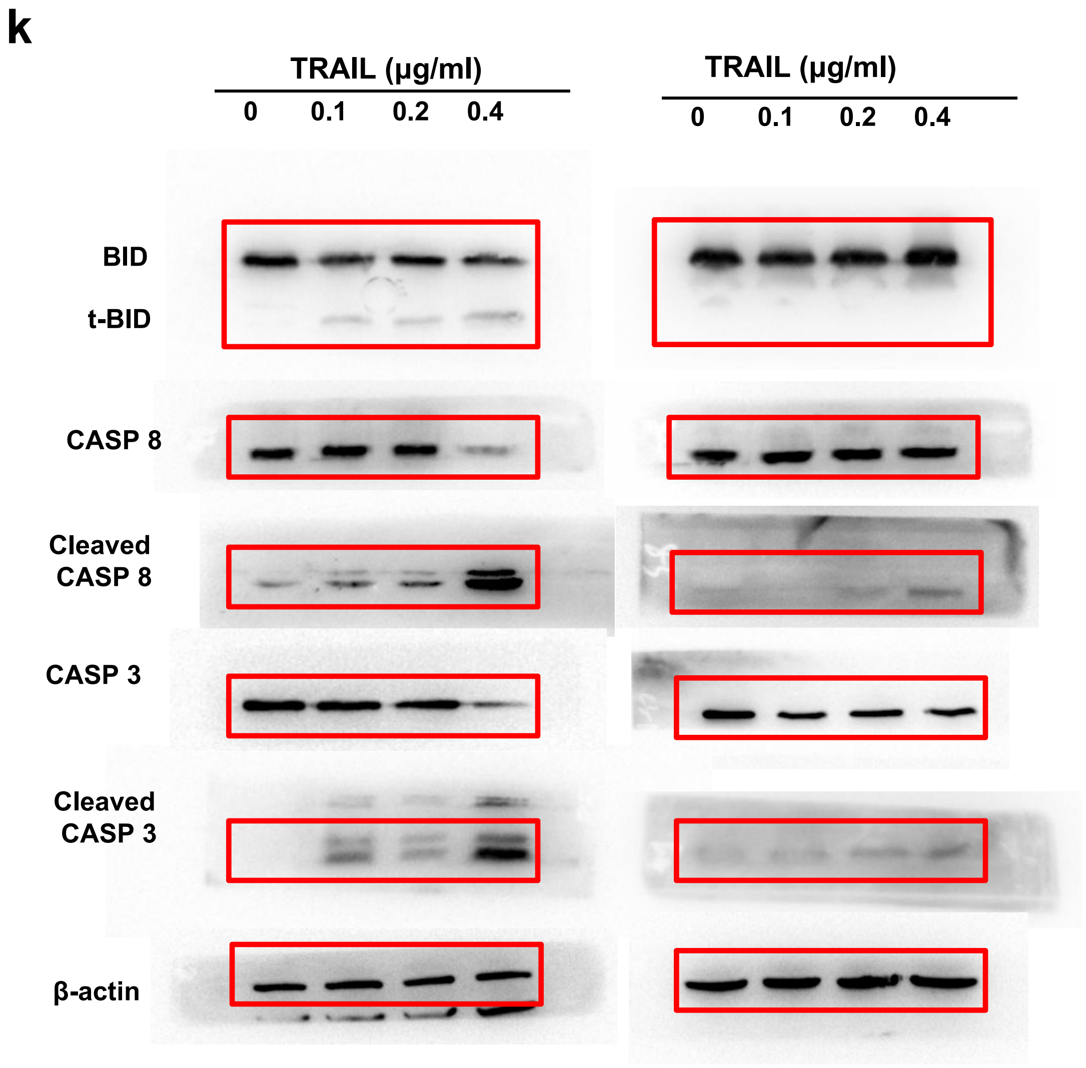
k
TRAIL (μg/ml)
0 0.1 0.2 0.4
TRAIL (μg/ml)
0 0.1 0.2 0.4
BID
t-BID
CASP 8
Cleaved CASP 8
CASP 3
Cleaved CASP 3
β-actin

Fig. 3

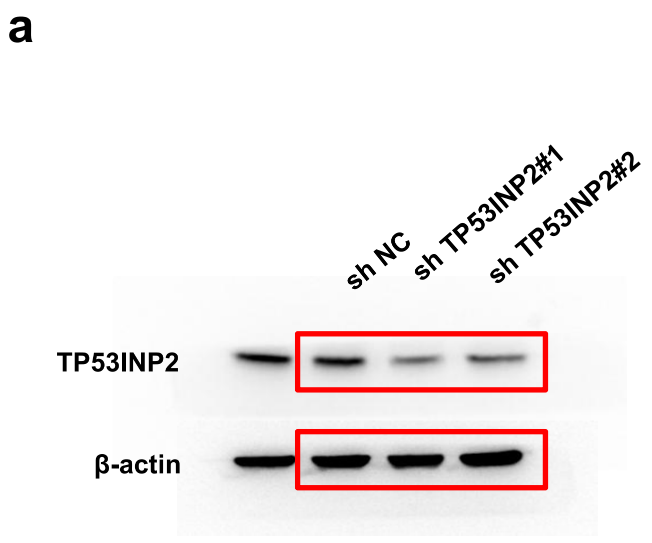
a
sh NC
sh TP53INP2#1
sh TP53INP2#2
TP53INP2
β-actin

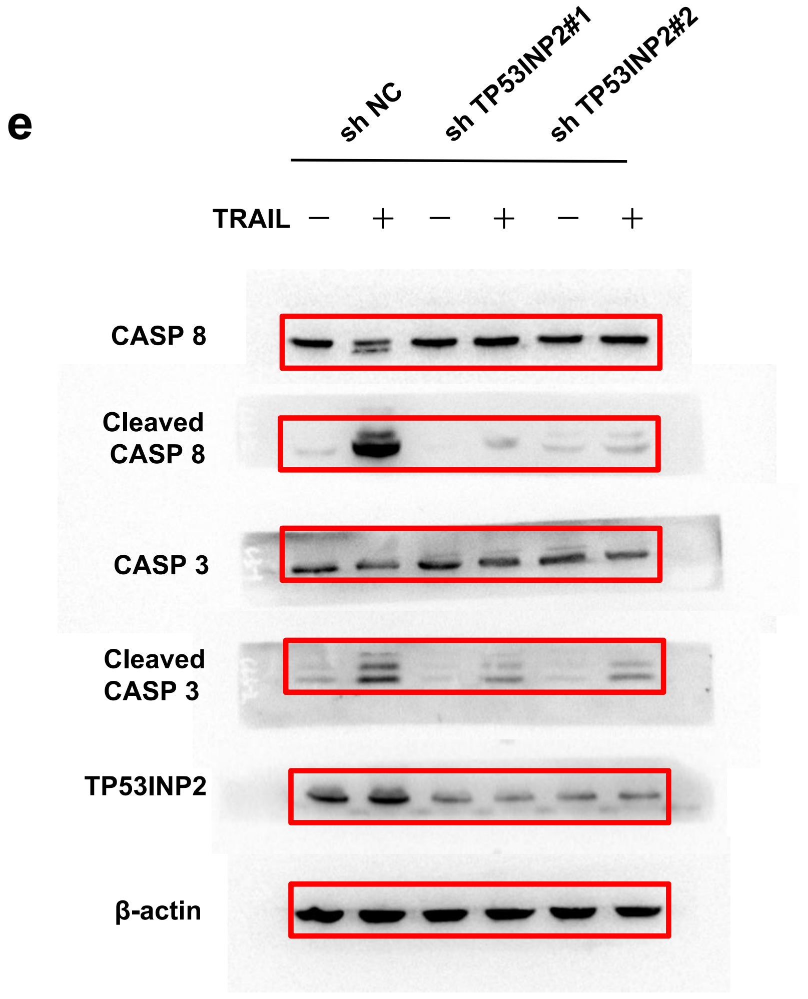
e
sh NC
sh TP53INP2#1
sh TP53INP2#2
TRAIL − + − + − +
CASP 8
Cleaved CASP 8
CASP 3
Cleaved CASP 3
TP53INP2
β-actin

Fig. 3

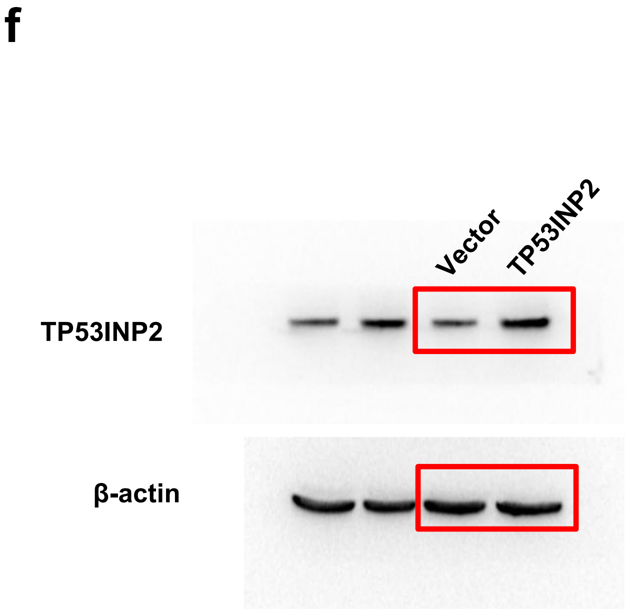
f
Vector
TP53INP2
TP53INP2
β-actin

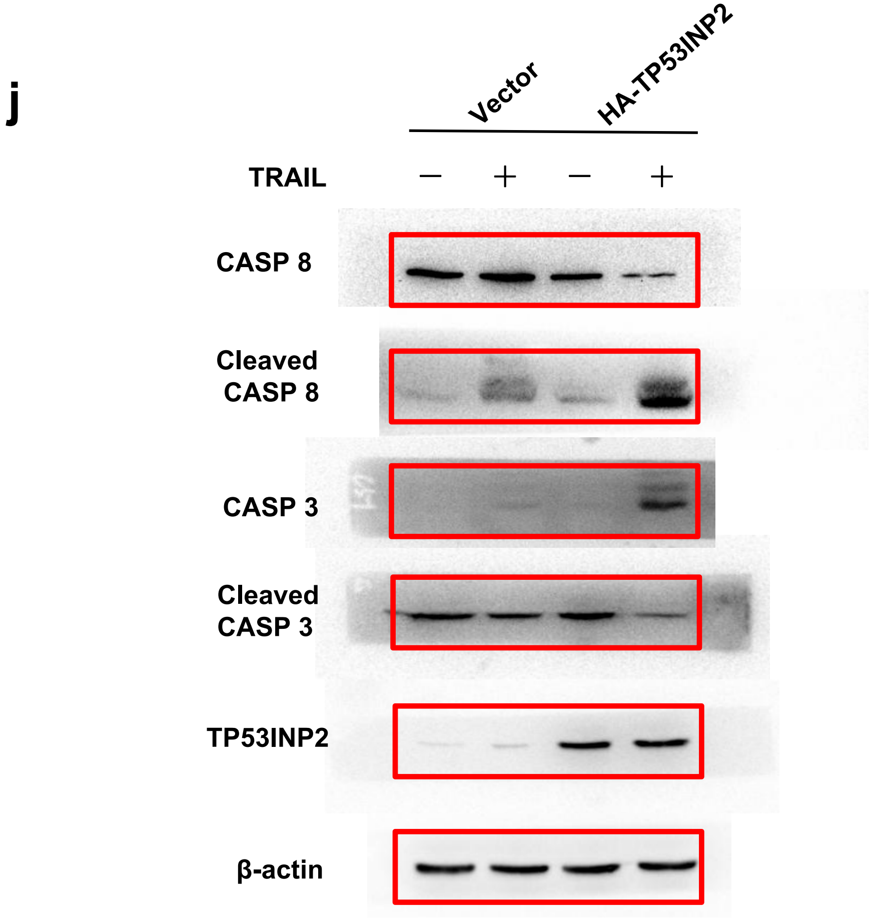
j
Vector
HA-TP53INP2
TRAIL
−
+
−
+
CASP 8
Cleaved CASP 8
CASP 3
Cleaved CASP 3
TP53INP2
β-actin

Fig. 4

a

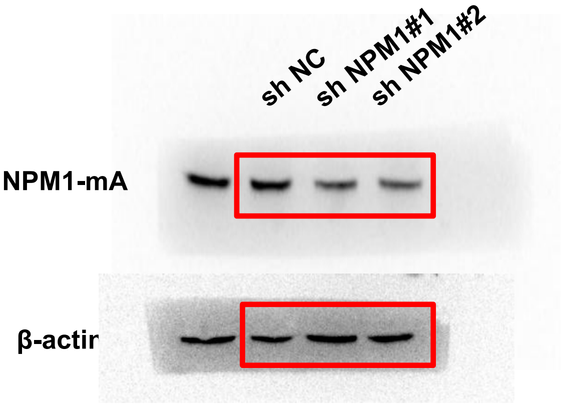

sh NC
sh NPM1#1
sh NPM1#2
NPM1-mA
β-actir

e

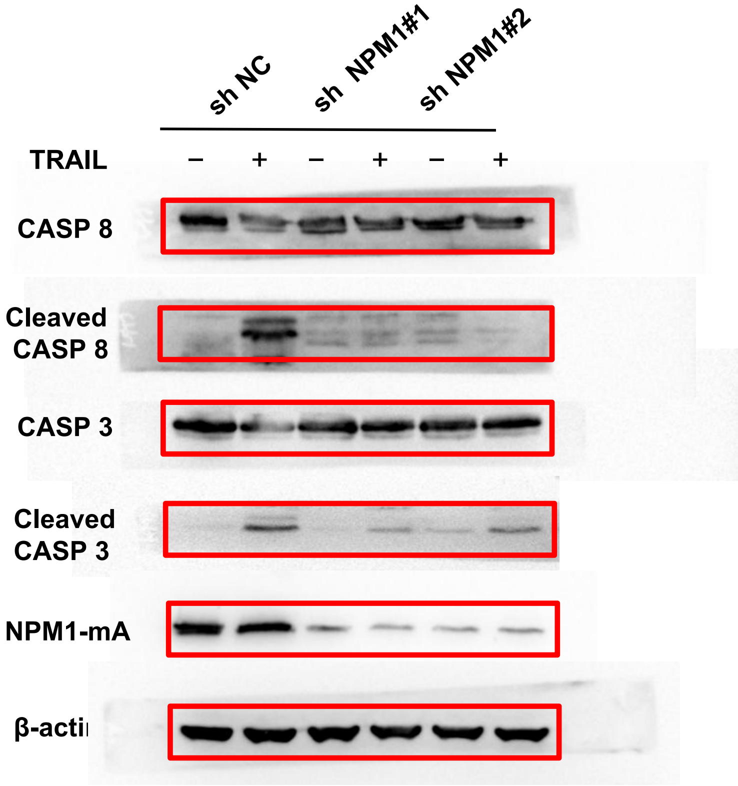

sh NC
sh NPM1#1
sh NPM1#2
TRAIL
− + − + − +
CASP 8
Cleaved CASP 8
CASP 3
Cleaved CASP 3
NPM1-mA
β-acti

Fig. 4

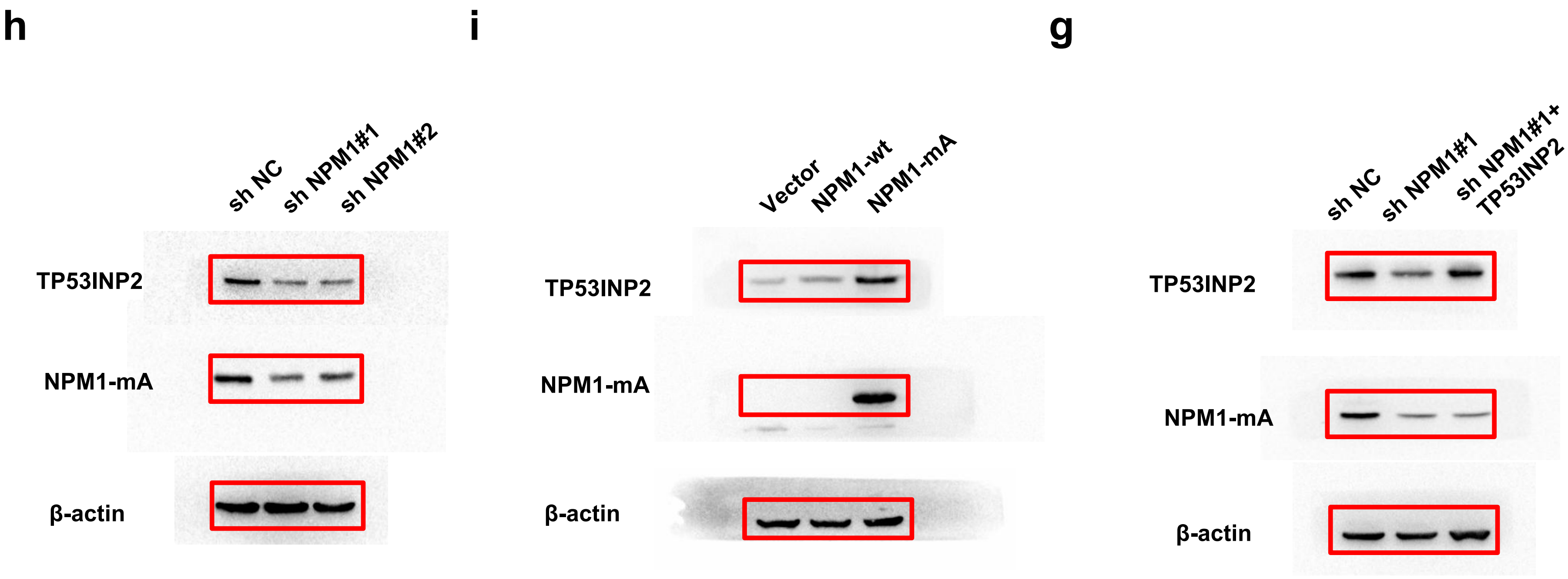
h
sh NC
sh NPM1#1
sh NPM1#2
TP53INP2
NPM1-mA
β-actin
i
Vector
NPM1-wt
NPM1-mA
TP53INP2
NPM1-mA
β-actin
g
sh NC
sh NPM1#1
sh NPM1#1+ TP53INP2
TP53INP2
NPM1-mA
β-actin

Fig. 5

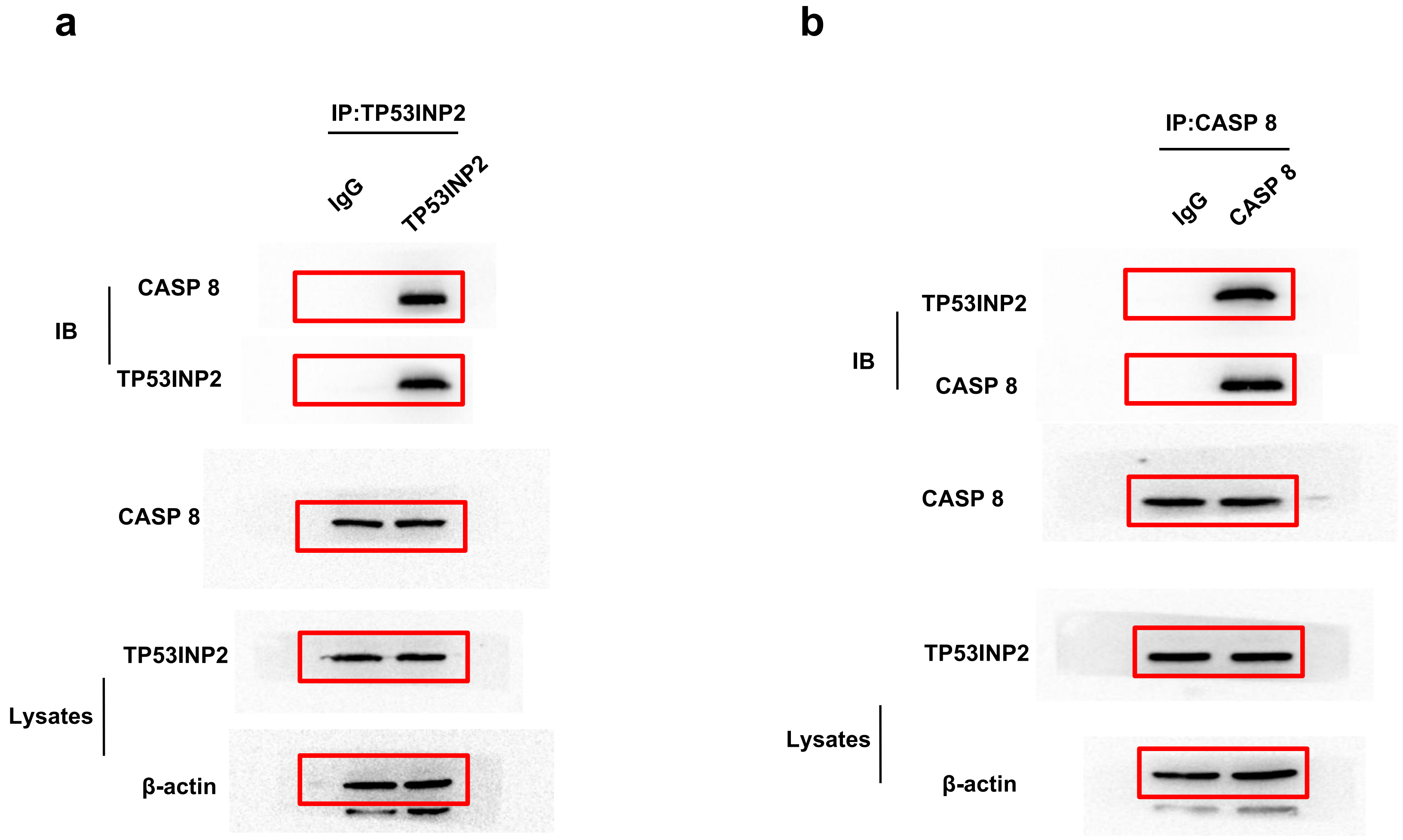
a
IP:TP53INP2
IgG
TP53INP2
IB
CASP 8
TP53INP2
CASP 8
TP53INP2
Lysates
β-actin
b
IP:CASP 8
IgG
CASP 8
IB
TP53INP2
CASP 8
CASP 8
TP53INP2
Lysates
β-actin

Fig. 5

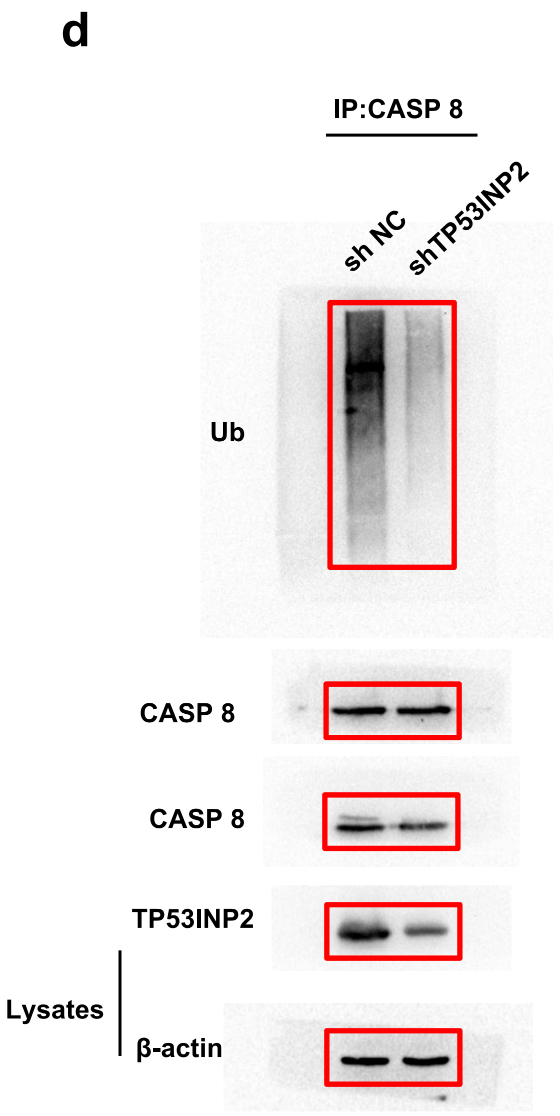

d
IP:CASP 8
sh NC
shTP53INP2
Ub
CASP 8
CASP 8
TP53INP2
Lysates
β-actin

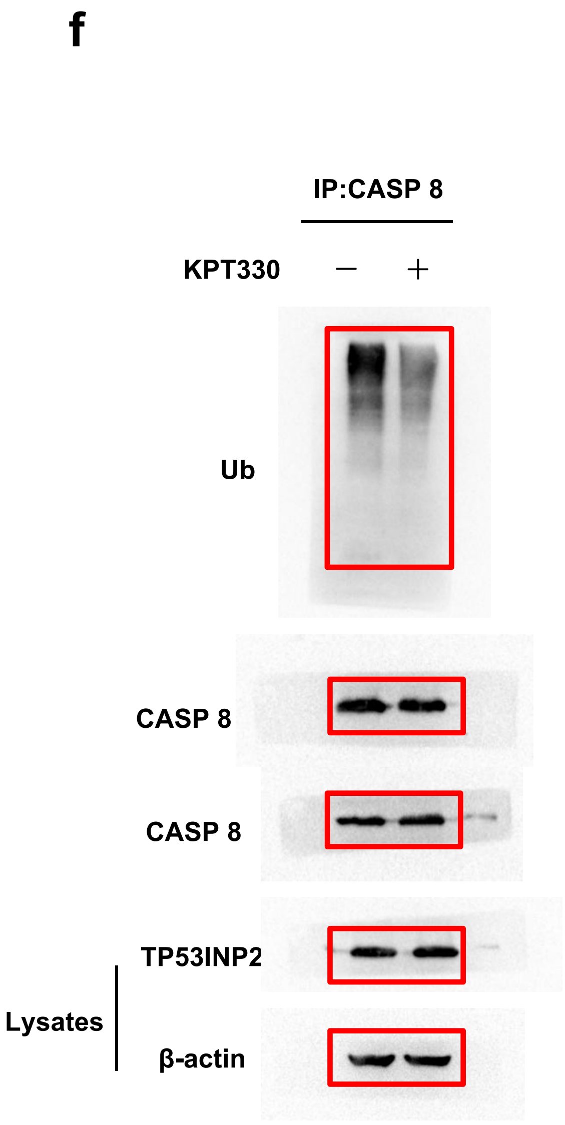

f
IP:CASP 8
KPT330
−
+
Ub
CASP 8
CASP 8
TP53INP2
Lysates
β-actin

Fig. 5

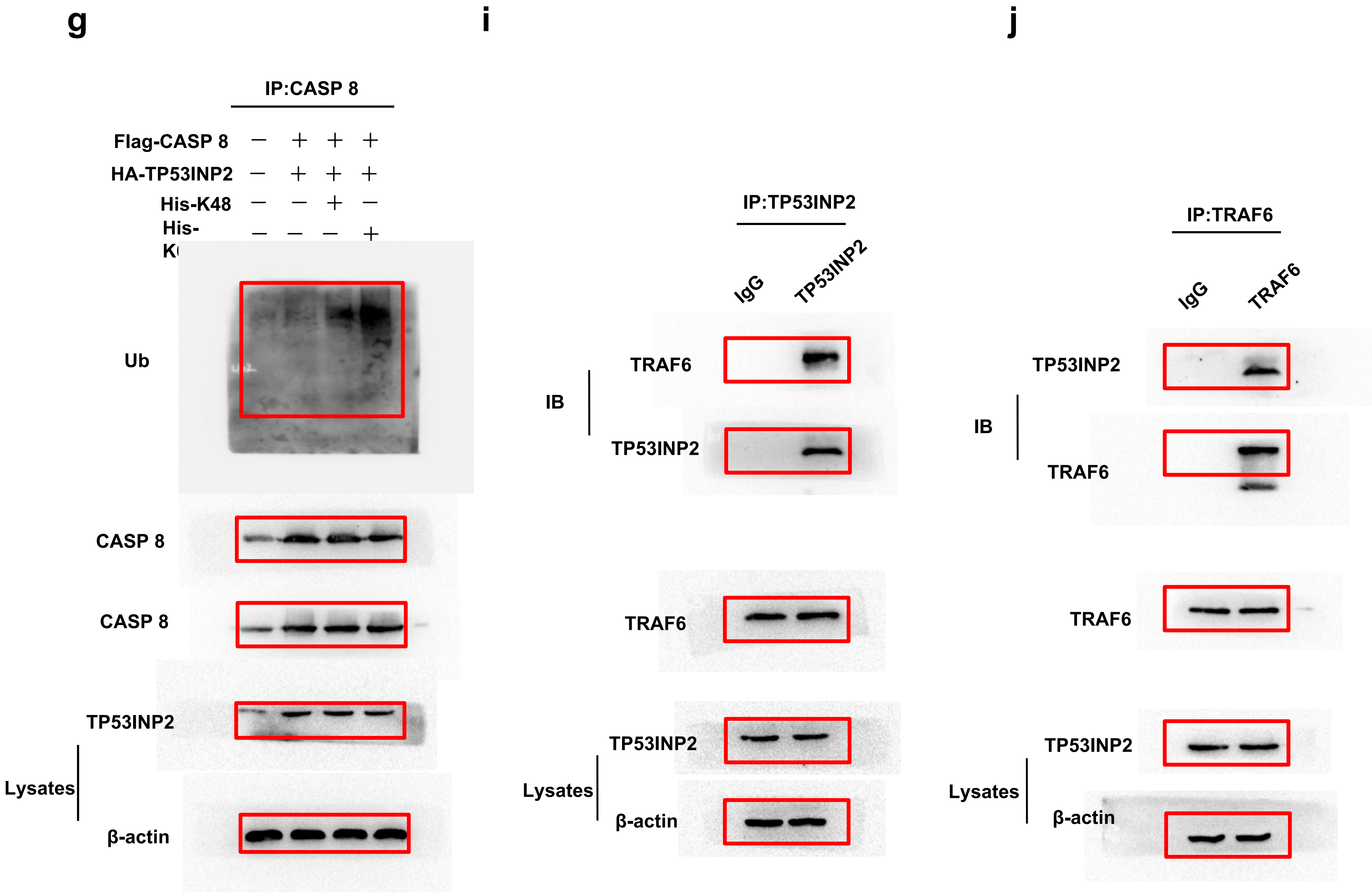
g
IP:CASP 8
Flag-CASP 8 − + + +
HA-TP53INP2 − + + +
His-K48 − − + −
His-
− − − +
Ub
CASP 8
CASP 8
TP53INP2
Lysates
β-actin
i
IP:TP53INP2
IgG
TP53INP2
IB
TRAF6
TP53INP2
TRAF6
TP53INP2
Lysates
β-actin
j
IP:TRAF6
IgG
TRAF6
IB
TP53INP2
TRAF6
TRAF6
TP53INP2
Lysates
β-actin

Fig. 5

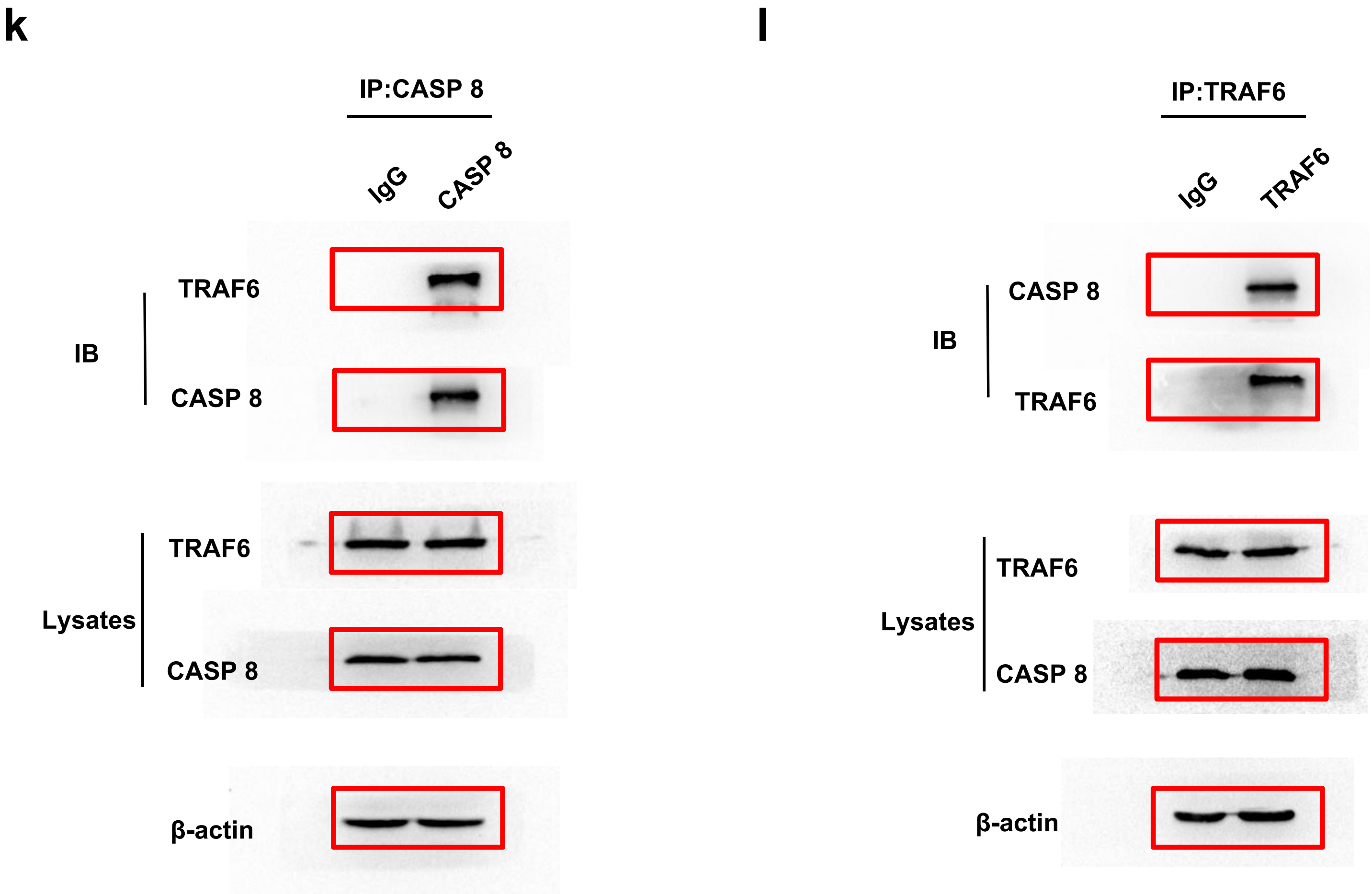

k
IP:CASP 8
IgG
CASP 8
IB
TRAF6
CASP 8
Lysates
TRAF6
CASP 8
β-actin
l
IP:TRAF6
IgG
TRAF6
IB
CASP 8
TRAF6
Lysates
TRAF6
CASP 8
β-actin

Fig. 5

m

n

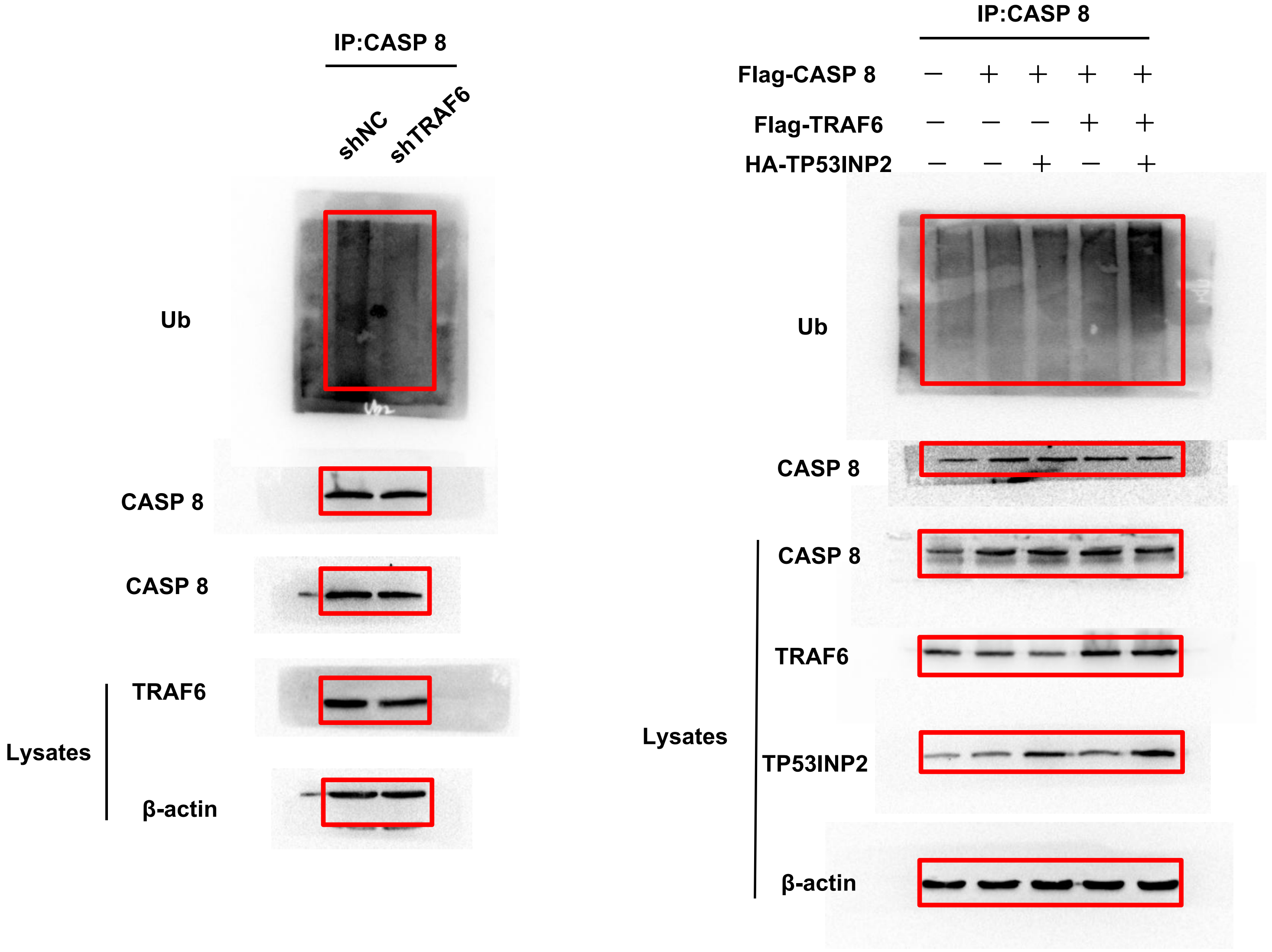
IP:CASP 8
shNC
shTRAF6
Ub
CASP 8
CASP 8
TRAF6
Lysates
β-actin
IP:CASP 8
Flag-CASP 8 − + + + +
Flag-TRAF6 − − − + +
HA-TP53INP2 − − + − +
Ub
CASP 8
CASP 8
TRAF6
Lysates
TP53INP2
β-actin

Fig. S1

a

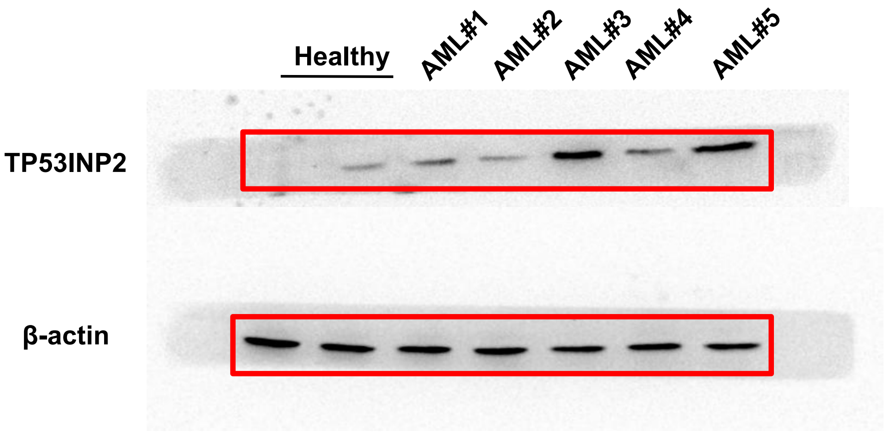
Healthy
AML#1
AML#2
AML#3
AML#4
AML#5
TP53INP2
β-actin

c

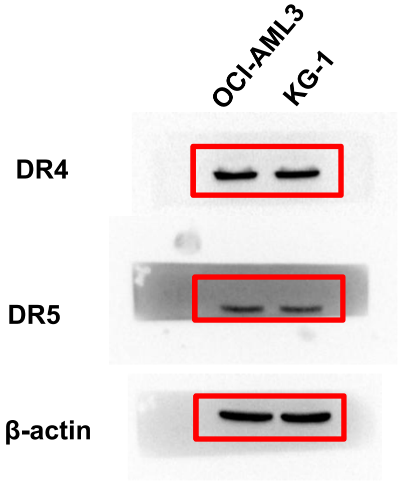
OCI-AML3
KG-1
DR4
DR5
β-actin

Fig. S2

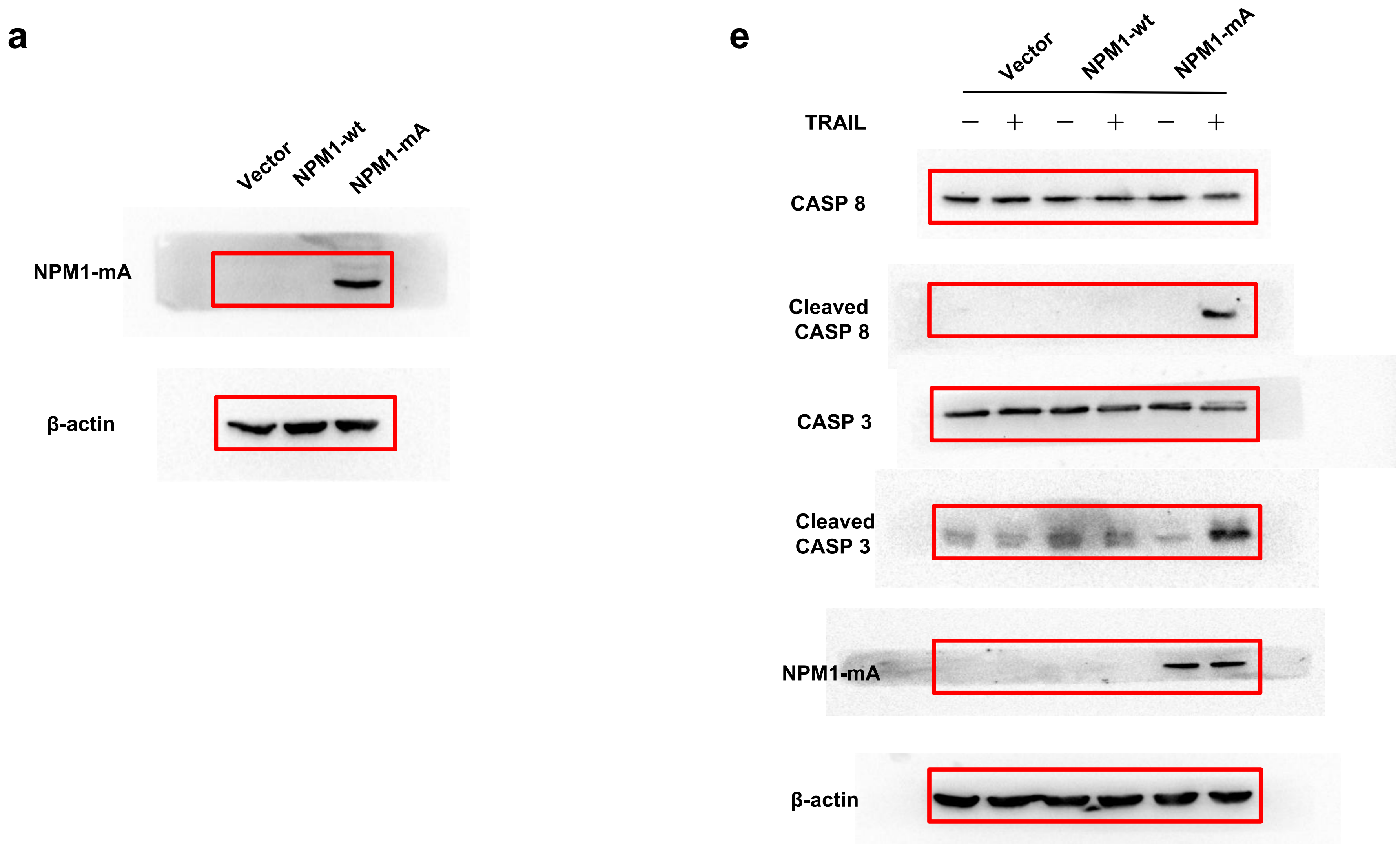
a
Vector
NPM1-wt
NPM1-mA
NPM1-mA
β-actin
e
Vector
NPM1-wt
NPM1-mA
TRAIL
− + − + − +
CASP 8
Cleaved CASP 8
CASP 3
Cleaved CASP 3
NPM1-mA
β-actin

Fig. S3

a

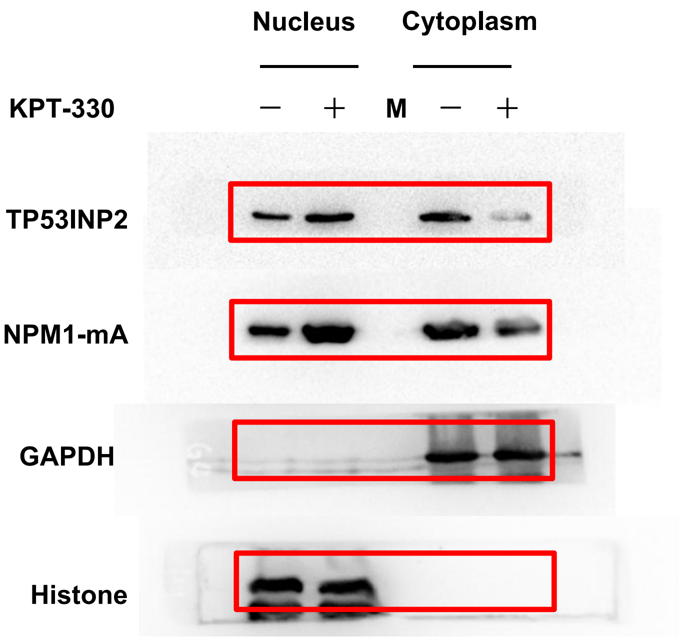

Supplement: Supplementary file 7 — Additional file 7: The full (not cropped) Western blot gel figures. [file 13046_2024_3100_MOESM7_ESM.pdf]
